# Redefining the Incidence and Profile of Fluoropyrimidine-Associated Cardiotoxicity in Cancer Patients: A Systematic Review and Meta-Analysis

**DOI:** 10.3390/ph16040510

**Published:** 2023-03-30

**Authors:** Yajie Lu, Wei Pan, Shizhou Deng, Qiongyi Dou, Xiangxu Wang, Qiang An, Xiaowen Wang, Hongchen Ji, Yue Hei, Yan Chen, Jingyue Yang, Hong-Mei Zhang

**Affiliations:** 1 Department of Clinical Oncology, Xijing Hospital, Air Force Medical University, Xi’an 710032, China; 2 The State Key Laboratory of Cancer Biology, Biotechnology Center, School of Pharmacy, Air Force Medical University, Xi’an 710032, China; 3 The Department of Biomedical Engineering, Air Force Medical University, Xi’an 710032, China

**Keywords:** fluoropyrimidine, cardiotoxicity, adverse event, incidence, meta-analysis

## Abstract

Aim: The cardiac toxicity that occurs during administration of anti-tumor agents has attracted increasing concern. Fluoropyrimidines have been used for more than half a century, but their cardiotoxicity has not been well clarified. In this study, we aimed to assess the incidence and profile of fluoropyrimidine-associated cardiotoxicity (FAC) comprehensively based on literature data. Methods: A systematic literature search was performed using PubMed, Embase, Medline, Web of Science, and Cochrane library databases and clinical trials on studies investigating FAC. The main outcome was a pooled incidence of FAC, and the secondary outcome was specific treatment-related cardiac AEs. Random or fixed effects modeling was used for pooled meta-analyses according to the heterogeneity assessment. PROSPERO registration number: (CRD42021282155). Results: A total of 211 studies involving 63,186 patients were included, covering 31 countries or regions in the world. The pooled incidence of FAC, by meta-analytic, was 5.04% for all grades and 1.5% for grade 3 or higher. A total of 0.29% of patients died due to severe cardiotoxicities. More than 38 cardiac AEs were identified, with cardiac ischemia (2.24%) and arrhythmia (1.85%) being the most frequent. We further performed the subgroup analyses and meta-regression to explore the source of heterogeneity, and compare the cardiotoxicity among different study-level characteristics, finding that the incidence of FAC varied significantly among different publication decades, country/regions, and genders. Patients with esophagus cancer had the highest risk of FAC (10.53%), while breast cancer patients had the lowest (3.66%). The treatment attribute, regimen, and dosage were significantly related to FAC. When compared with chemotherapeutic drugs or targeted agents, such a risk was remarkably increased (χ^2^ = 10.15, *p* < 0.01; χ^2^ = 10.77, *p* < 0.01). The continuous 5-FU infusion for 3–5 consecutive days with a high dosage produced the highest FAC incidence (7.3%) compared with other low-dose administration patterns. Conclusions: Our study provides comprehensive global data on the incidence and profile of FAC. Different cancer types and treatment appear to have varying cardiotoxicities. Combination therapy, high cumulative dose, addition of anthracyclines, and pre-existing heart disease potentially increase the risk of FAC.

## 1. Introduction

Fluoropyrimidines including 5-FU, its oral pro-drug capecitabine, the recent compound preparations S-1 (tegafur), and TAS 102 have been used in the treatment of solid tumors for more than half a century [1,2]. Although numerous therapeutic strategies have been introduced in recent years, such as targeted therapy [3] and immunotherapy [4], fluoropyrimidines are still the fundamental chemotherapeutic agents for treating many tumors [5], playing a significant role in disease control and prolongation of patient survival. However, serious adverse effects (AEs) associated with fluoropyrimidines may result in the modification of the prescribed treatment, or even its interruption. In particular, fluoropyrimidine-associated cardiotoxicity (FAC) is a potential threat for an effective chemotherapy, increasing the mortality for patients predicted to have long-term oncologic survival [6,7]. In recent years, cardiovascular toxicity related to cancer treatment has gained more and more clinical concerns. With the rise of the new discipline “cardio-oncology”, treatment-related cardiotoxicity, such as that of anthracyclines and immune checkpoint inhibitors (ICIs), has become a growing concern in recent decades [8,9]. Cardiotoxicity induced by fluoropyrimidine, however, has not received equal attention (Supplementary Figure S1). In fact, fluoropyrimidine seems to be one of the most common agents causing cardiotoxicity, second only to anthracycline [10,11]. The reported incidence of FAC ranges from 0 to 68% [12,13,14,15]; however, these data are imprecise and valueless for clinical reference due to the risk of bias. The described cardiac AEs of FAC encompass a broad spectrum, including ischemia, arrhythmia, angina, heart failure, cardiac arrest, enzyme change, cardiomyopathy, myocarditis, and so on [13]. Unfortunately, uncertainty remains regarding the profile of FAC within the published data.

The accurate estimation of the true incidence of FAC and profile of cardiac AEs is essential for preventing severe events and ensuring therapeutic efficacy, and is critical for scientific research. In this study, we represented a meta-analysis across mono or combined fluoropyrimidine therapies, in order to obtain the exact incidence of FAC and profile of cardiac AEs, and to explore further the potential differences in FACs among a variety of demographic characteristics, tumors, study designs, and treatment regimens.

## 2. Materials and Methods

### 2.1. Search Strategy and Selection Criteria

A systematic literature search was performed to identify studies that evaluated fluoropyrimidine-related cardiotoxicity. PubMed, Embase, Medline, Web of Science, Cochrane library, and clinical trials (https://clinicaltrials.gov/ accessed on 20 March 2023) were retrieved from the establishment of each database/website to 31 October 2022, with no language restrictions. The reference lists of the relevant articles were also reviewed to avoid the omission of eligible studies. The retrieval scheme used for each database is shown in Supplementary Table S1.

Studies eligible for inclusion met all the following criteria: (1) definitive diagnosis of solid malignances; (2) involving fluoropyrimidine-based treatment, including 5-FU, capecitabine, S-1, and/or TAS 102; (3) the required data were available; (3) treatment-related cardiotoxicity was explicitly reported; (4) prospective or retrospective clinical study. The following exclusion criteria were used: (1) phase I clinical trials; (2) the sample size was smaller than 10; (3) animal experiments or laboratory research; (4) reviews, meta-analyses, comments, or case reports. This work was performed under the guidance of the PRISMA statement [16], and the protocol was registered in PROSPERO (No. CRD42021282155) [17].

### 2.2. Methodological Quality Assessment

The quality assessment tool of the National Institutes of Health (NIH) (Supplementary Table S2) was used to assess the methodological quality of the included studies [18]. Each single-arm study was assessed according to a list of 9 items, while the controlled study was assessed against a list of 14 items. For each item, reviewers could select “YES”, “NO”, or “Cannot Determine/Not Applicable/Not Reported”. Based on their responses, each study was then graded as being of “good”, “fair”, or “poor” quality.

### 2.3. Outcomes of Interest and Data Extraction

The main outcome was the incidence of the cardiotoxicities for all grades, grade 3 or higher, and grades 1–2. The profiles of cardiac AEs were also prespecified as important secondary outcomes. The data of basic characteristics, treatment details, and clinical results of cardiotoxicity were obtained from each included study.

### 2.4. Statistical Analysis

The meta-analyses were conducted using R software (Version 4.0.6) with “meta”, “rmeta”, and “metafor” packages. Shapiro–Wilk normality tests on the raw rate and transformed data (log, logit, arcsine, and Freeman–Tukey transformation) were used to determine the most appropriate data type for the pooled analysis. The inter-study heterogeneity was detected by the Cochran’s Q test reporting I^2^ statistic and *p* values. A random-effect model was adopted in case of an indication of significant heterogeneity (I^2^ > 50% or *p* < 0.1) [19,20], otherwise, the fixed-effect model was used. Subgroup analyses were performed based on study-level moderators in order to compare the incidence of FAC among studies with different characteristics. A multilevel meta-regression analysis was conducted to detect the source of heterogeneity further and examine the influence of the moderator variables. Funnel plot and Egger’s tests were used to assess publication bias. The sensitivity analysis was performed by excluding studies one by one to determine the stability of the results of the meta-analysis. 

## 3. Results

### 3.1. Eligible Studies and Characteristics

**Figure 1:**
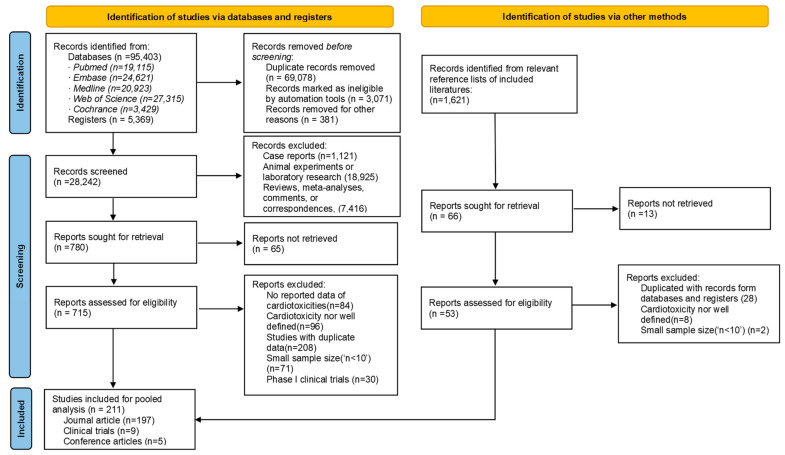
Flow diagram of the inclusion/exclusion process of the relevant literature with number of articles at each step.

**Figure 2:**
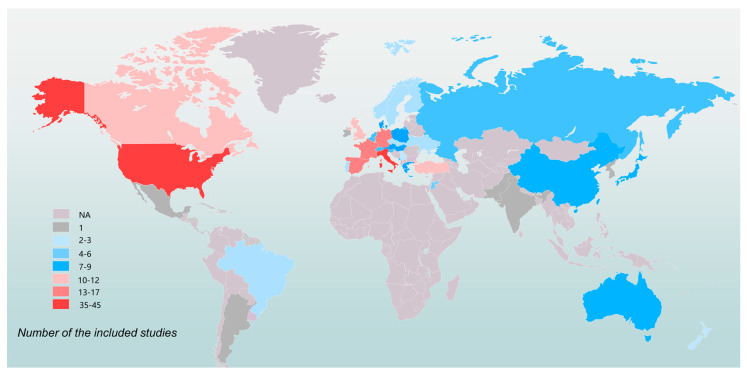
Geographical distribution of the 211 included studies.

A total of 211 eligible studies involving 63,186 patients were included [15,21,22,23,24,25,26,27,28,29,30,31,32,33,34,35,36,37,38,39,40,41,42,43,44,45,46,47,48,49,50,51,52,53,54,55,56,57,58,59,60,61,62,63,64,65,66,67,68,69,70,71,72,73,74,75,76,77,78,79,80,81,82,83,84,85,86,87,88,89,90,91,92,93,94,95,96,97,98,99,100,101,102,103,104,105,106,107,108,109,110,111,112,113,114,115,116,117,118,119,120,121,122,123,124,125,126,127,128,129,130,131,132,133,134,135,136,137,138,139,140,141,142,143,144,145,146,147,148,149,150,151,152,153,154,155,156,157,158,159,160,161,162,163,164,165,166,167,168,169,170,171,172,173,174,175,176,177,178,179,180,181,182,183,184,185,186,187,188,189,190,191,192,193,194,195,196,197,198,199,200,201,202,203,204,205,206,207,208,209,210,211,212,213,214,215,216,217,218,219,220,221,222,223,224,225,226,227,228,229,230] (Figure 1). Table 1 summarizes the characteristics of the included studies. The involving population covered 31 countries or regions in the world (Figure 2). According to the NIH quality assessment tools, 61 articles (28.9%) had a good quality score, 150 (71.1%) fair quality, and none was classified as poor (high risk of bias). The detailed information of each included study is shown in Supplementary Table S3.

### 3.2. Pooled Incidence of FAC

Our analysis generated robust data on FAC incidence. A total of 186 studies with 40,170 patients were enrolled in the pooled analysis of all-grade cardiac AEs, and 2285 (5.68%) patients experienced at least one cardiac AE. The pooled incidence of all-grade FAC was 5.04% (95% CI 4.21–5.94%) (Supplementary Figure S2). The pooled incidence of cardiac AEs grade 3 or higher was 1.5% (95% CI 1.09–1.96%), involving 127 studies with 25,273 participants (Supplementary Figure S3). A total of 718 individuals had cardiac AEs grade 1–2, with a pooled incidence of 2.33% (95% CI 1.57–3.21%) (Supplementary Figure S4).

### 3.3. Profile of the Cardiac AEs

**Figure 3:**
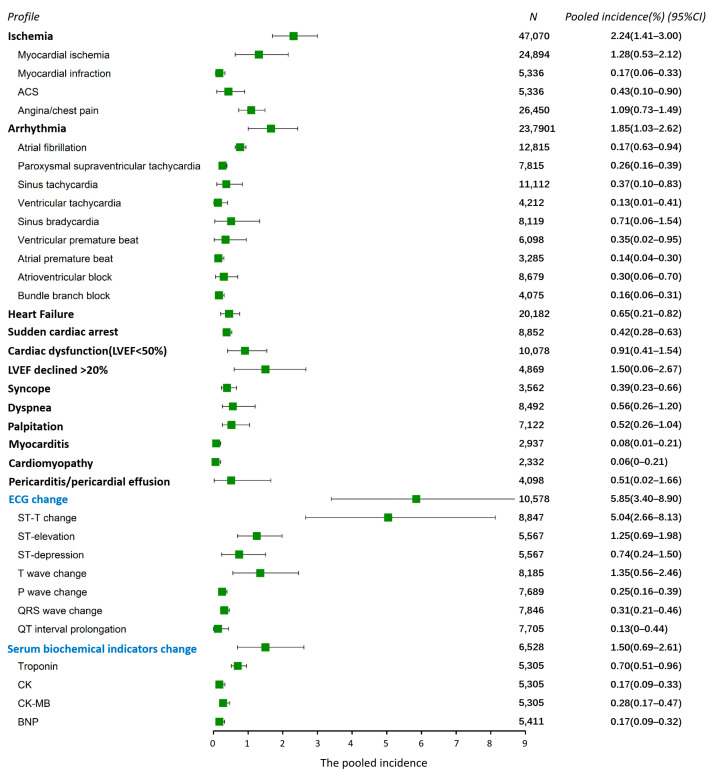
The profile of the FAC (all-grade cardiac AEs). Each bar and square represent the pooled value of incidence and 95% confidence interval for one cardiac AE. Vertical line indicates the overall incidence of all-grade adverse events (5.04%). Abbreviations: ACS, acute coronary syndrome; LVEF, left ventricular ejection fraction; CK, creatine kinase; BNP, B-type natriuretic peptide. “*N*” at the top of this figure represents the number of patients included in the evaluation for each AE.

The profile of FAC includes a variety of disease and symptoms, and, in our study, more than 38 different types of cardiac AEs were reported (Figure 3). From the results of this analysis, cardiac ischemia and arrhythmia were the two most common AEs, occurring in 2.24% (95% CI 1.41–3.00%) and 1.85% (95% CI 1.03–2.62%) of patients, respectively. Heart failure developed in 0.65% of the population (95% CI 0.21–0.82%), probably as a result of severe events following cardiac ischemia and arrhythmia. The left ventricular ejection fraction (LVEF) decreased ≥20% in 1.5% (95% CI 0.6–2.67%) of patients, and 0.91% (95% CI 0.41–1.54%) had cardiac dysfunction (LVEF < 50%). ECG alterations were reported in 37 studies, with a pooled incidence of 5.85% (95% CI 3.4–8.9%), slightly higher than the incidence of FAC (5.18%), indicating asymptomatic ECG alterations in a subset of the population. The ST-T change was the most frequently observed (5.04%, 95% CI 2.66–8.13%), which represents an indicator of cardiac ischemia. Additionally, serum biochemical changes were reported in 16 studies, with a pooled incidence of 1.5% (95% CI 0.69–2.61%).

### 3.4. FAC-Related Deaths

The pooled mortality of FAC was 0.12% (95% CI 0.08–0.15%), involving 114 cases (0.29%, 114/39, 455) from 194 studies. The most frequent causes of cardiotoxicity-related death were sudden cardiac arrest (28.95%) and myocardial infarction (27.19%). Heart failure (15.79%) and severe arrhythmias (14.04%) were also common causes of cardiac death (Table 2).

### 3.5. Factors Influencing the Occurrence of FAC-Subgroup Analysis

#### 3.5.1. Basic Demographic and Study-Level Factors

**Figure 4:**
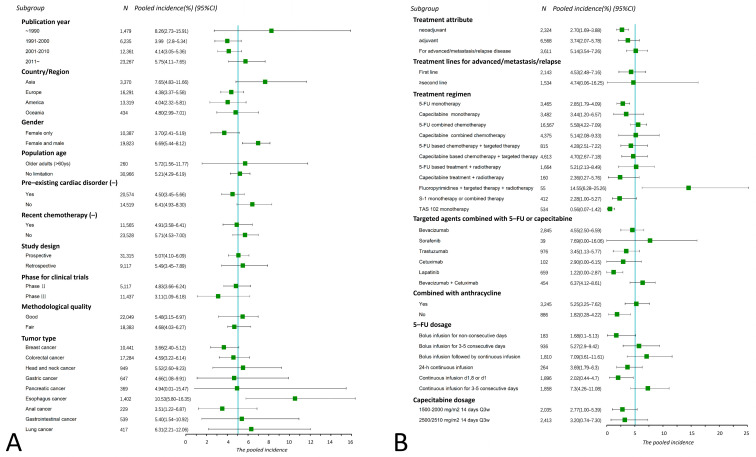
The results of subgroup analysis outcomes of all-grade FAC by basic characteristics (**A**) and treatment details (**B**) of included studies. Each bar and square represent the pooled value of incidence and 95% confidence interval for one subgroup. Vertical line indicates the pooled incidence of all-grade FAC (5.04%); “*N*” at the top of this figure represents the number of patients included in the evaluation for each subgroup.

The results of the subgroup analysis are shown in Figure 4. The incidence of FAC has significantly increased in the past three decades (χ^2^ = 7.8, *p* = 0.02). Studies conducted in Asia outlined a higher incidence than in Europe (χ^2^ = 4.44, *p* = 0.03) and America (χ^2^ = 4.45, *p* = 0.03). No significant difference was observed between their subgroups in terms of study design, trial phase, population age, and methodological quality. Sixty-three studies only included females with breast cancer, producing a lower incidence of FAC than those involving both females and males (χ^2^ = 8.75, *p* < 0.01). Studies that excluded people with pre-existing cardiac disorders had a reduced incidence of FAC (χ^2^ = 4.29, *p* = 0.04).

#### 3.5.2. FAC for Different Cancers

The incidence of FAC varies among different cancers (Figure 4A). The highest pooled incidence was observed in esophagus cancer (10.53%, 95% CI 5.8–16.35%), significantly greater than breast cancer (3.66%, χ^2^ = 8.04, *p* < 0.01) and colorectal cancer (4.59%, χ^2^ = 5.24, *p* = 0.02). The lowest incidence of FAC occurred in breast cancer (3.66%, 95% CI 2.4–5.12%), but no statistical difference was identified when compared with colorectal cancer (χ^2^ = 1.95, *p* = 0.16), head and neck cancer (5.52%, χ^2^ = 1.79, *p* = 0.18), gastric cancer (4.66%, χ^2^ = 0.650, *p* = 0.42), and pancreatic cancer (4.94%, χ^2^ = 0.08, *p* = 0.77). The lung cancer subgroup had the second highest incidence of cardiotoxicity (6.31% 95% CI 2.21–12.06%); however, these studies were from the 1990s, and fluoropyrimidines are now no longer recommended for use in lung cancer.

#### 3.5.3. FAC for Different Treatment Parameters

Significant differences in FAC incidence existed between treatment parameters. Fluoropyrimidines for advanced/metastatic/relapsed diseases had a higher incidence of FAC compared with neoadjuvant or adjuvant treatments (χ^2^ = 6.91, *p* = 0.03); however, no statistical difference was observed between different treatment lines (first-line vs. ≥ second-line, χ^2^ = 0.05, *p* = 0.82). The 5-FU induced cardiotoxicity in monotherapy was significantly lower than that in the combination therapy, either combined with other chemotherapeutic drugs (χ^2^ = 10.15, *p* < 0.01) or targeted agents (χ^2^ = 10.77, *p* < 0.01).

Anthracycline agents and anti-angiogenic drugs are known to cause cardiotoxicity, and our results showed a significantly higher incidence of cardiotoxicity when fluoropyrimidines were combined with anthracyclines (χ^2^ = 4.02, *p* = 0.04) or anti-angiogenic targeted agents (χ^2^ = 15.73, *p* < 0.01) (Figure 4B). The incidence of capecitabine-induced FAC was slightly higher than that of 5-FU (3.44% vs. 2.85%), but without statistical significance (χ^2^ = 0.01, *p* = 0.97). In addition, the compound preparations S1 and TAS 102 showed a relatively low incidence of FAC (S1 2.28%, TAS 102 0.56%).

The occurrence of cardiotoxicity was closely related to drug administration patterns (χ^2^ = 12.29, *p* = 0.03) (Supplementary Figure S5). The continuous 5-FU infusion for 3–5 consecutive days produced the highest incidence (7.3%). The second highest occurred at the dosage pattern of bolus infusion, followed by continuous infusion (7.09%). Non-consecutive infusion (1.68%), 24 h continuous infusion (3.69%), and continuous infusion on d1,8 or d1 (2.02%) resulted in a relatively low incidence. In fact, a significant positive correlation was identified between the cumulative 5-FU dose per cycle and the cardiotoxicity (χ^2^ = 8.41, *p* = 0.04) (Supplementary Figure S6). A dosage greater than 3000 mg/m^2^ resulted in a three-fold higher toxicity than the lower dose (≤1000 mg/m^2^) (χ^2^ = 7.66, *p* < 0.01).

### 3.6. The Results of Meta-Regression Analysis

A meta-regression analysis was performed to identify the factors influencing FAC incidence further. The univariable meta-regression of continuous data revealed that female proportion (negative, Q = 8.59, *p* < 0.01) and 5-FU dosage (positive, Q = 9.57, *p* < 0.01) were strongly correlated with FAC (Supplementary Figure S7A,B). Publication year and median age of population were also correlated with the cardiotoxicity, but the differences were not significant (*p* = 0.058; *p* = 0.071) (Supplementary Figure S7C,D). Results of the multilevel meta-regression analysis are shown in Table 3. As can be seen in the results, the moderators of publication year (*p* = 0.02), country/region (*p* < 0.01), pre-existing cardiac disorders (*p* < 0.01), study design (*p* = 0.024), treatment attribute (*p* < 0.001), cancer type (*p* = 0.026), regimen (*p* = 0.043), and anthracycline combination (*p* = 0.027) were significant predictors influencing the occurrence of cardiotoxicities. This multilevel meta-regression model totally explained more than half of the inter-study heterogeneity (R^2^ = 51.74%).

### 3.7. Publication Bias and Sensitivity Analysis

No obvious asymmetry was observed in the funnel plots of the main outcomes, suggesting no evidence of significant publication bias, which was confirmed by the Egger’s test (Supplementary Figure S8A–F). The results of the sensitivity analysis showed that no individual study substantially influenced the pooled results of the above main outcomes (Supplementary Figure S9A–F), indicating that the results of this meta-analysis were relatively stable.

## 4. Discussion

This study generated robust epidemiological data of FAC incidence and profile based on a single-rate meta-analysis of 211 studies and 63,186 patients, which revised the previous over- or under-estimation of FAC [31,38,231]. We further identified several factors influencing the occurrence of FAC through subgroup analysis and meta-regression analysis. Cardiotoxicity caused by fluoropyrimidines has not been widely recognized and studied in the past. However, along with the wide application of fluoropyrimidine-combined therapy and the increasing demand of long-term mediation for advanced-stage patients with improved survival, increasingly attention has been paid to the cardiotoxicity problem of fluoropyrimidines. The incidence of FAC (5.04%), by our meta-analytic, is second only or even comparable to the familiar cardiotoxicity of anthracyclines (data from a meta-analysis based on 50 thousand cases: The incidence of 6.3%) [232]. Indeed, the real prevalence of FAC might be even higher than what we reported, since not all included studies undertook the most comprehensive cardiac evaluation, which might have resulted in missed FAC detections. Therefore, FAC must be given a high level of attention in clinical practice, as capecitabine, 5-FU, and its modified agents are increasingly used in maintenance therapy for malignancies. The management of FAC in cancer patients has a tremendous impact on the type of antitumor therapies, as well as long-term morbidity and mortality [233].

The profile of fluoropyrimidine-related cardiac AEs has been clearly depicted in our results, which provided evidence for close monitoring and early identification of pertinent cardiac symptoms and signs. Cardiac ischemia is the typical and most common FAC, occurring in 2.24% of patients, and one of the most common causes of fluoropyrimidine-related death (27.19%). The typical ST-T ECG changes were observed in most patients with cardiac ischemia. However, the overall incidence of ECG alterations was even higher than the incidence of cardiac AEs (5.85% vs. 5.04%), indicating that some subjects presented asymptomatic ECG changes, which is often a warning signaling an imminent ischemic event. Several studies considered patients who underwent 24 h ECG Holter monitoring, and, as a result, ischemic ECG changes were observed in 31–68% of patients [233,234]. Therefore, continuous ECG monitoring should be strongly recommended in patients subjected to fluoropyrimidines as the simplest and most useful method for early recognition of FAC. Arrhythmia, including atrial fibrillation, tachycardia, bradycardia, and conduction disorder were also indicated as the common features of FACs (1.85%), which might be a result of cardiac apoptosis triggered by ischemic change and cardiomyocyte damage [235]. LVEF can be used as an indicator of cardiac pump function, which is closely related to heart failure [236]. Our results showed a reduction in LVEF ≥ 20% in 1.5% of patients, while 0.91% developed cardiac dysfunction. However, the evaluation of LVEF using echocardiography is limited by the intrinsic operator-dependency of this technique. Thus, multi-level detection (e.g., ECG, echocardiography, myocardial enzyme, fMRI, coronary angiography, and radionuclide ventriculography) would be helpful for improving the sensitivity required to identify FAC.

Pre-treatment FAC risk assessment should ideally be performed, and the HFA-ICOS risk assessment tools can be considered [233]. Several studies suggested that FACs varied with the population characteristics, cancer types, and treatment regimens [237]. In contrast, other studies found that these factors were insignificant [95]. Our study performed a comprehensive sub-analysis to assess the influences of these variations on FAC, which is useful for indicating the risk factors of FAC. Being female was reported to be a protective factor for myocardial infarction and ischemia [238]. As shown in our results, the incidence of FAC decreased as the proportion of females increased, and the female-only population had a significant lower incidence than the general population. The lower cardiotoxicity was probably due, to some extent, to the protective effect of female hormones. On the other hand, it could also be related to the specific cancer type and regimens, since all patients in the female-only subgroup suffered from breast cancer, and most of them underwent capecitabine-based regimens (1000 mg/m^2^, d1–14, Q3w).

Previous studies reported a higher incidence of FAC in the elderly population [42], and it was reported that patients with an age ≥ 80 years are at great risk of treatment-related cardiac problems [233]. Similarly, we found a positive correlation between median age and cardiotoxicity, but no significant difference was detected in the subgroup analysis. Moreover, only four studies were included in the elderly subgroup (≥60 years) involving only 260 patients. Thus, it was not possible in this work to demonstrate whether the elderly is at particularly risk of FAC. Further large-scale stratified analyses focusing on the elderly are required. Notably, studies performed in Asia showed a higher incidence of cardiotoxicities than those in Europe and America. This result was consistent with the previous findings of Peng et al. [44], in which the incidence of cardiotoxicity in the Chinese population was higher than that in the non-Chinese population (25% vs. 19.9%). Such discrepancies may be derived from the genetic polymorphisms characterizing different ethnicities. For example, the frequency of DPD enzymatic activity varies greatly among Asian, Caucasian, and African-American populations, and it is an indicator of a remarkable risk for cardiac damage [239]. In addition, it is not surprising that pre-existing cardiac disorder significantly increased the risk of cardiotoxicities. One potential reason is that the unrecovered disease causes damage to the cardiomyocytes, making them more sensitive to an external stimulus like 5-FU infusion. Therefore, special care should be taken when administering fluoropyrimidines to patients with underlying cardiac disorders.

Our results indicated that the incidence of FAC differed among tumors. The highest incidence of cardiotoxicities was observed in esophageal cancer (10.53%), whereas the lowest was found in breast cancer (3.66%). Meydan’s study also showed a similar result [124], in which no patient with breast cancer suffered from cardiotoxicity, while the incidence in other tumors, such as colorectal cancer, was more than 3.5%. The discrepancy in cardiotoxicity among tumors may also be derived from gender, treatment regimen, or dosage schedule. For example, the addition of radiotherapy for esophageal cancer greatly increases the treatment-related cardiotoxicity. However, further studies on the FAC among different tumors are required because of the lack of direct comparisons between tumors.

The occurrence of cardiotoxicity varied among different fluoropyrimidine regimens. Combination therapy with other chemotherapy agents or targeted drugs resulted in a higher incidence of FAC than monotherapy. The increased cardiotoxicity could be due to the additional or synergistic deleterious cardiac effects from multiple agents. For example, it is worth noting that the cardiotoxicity significantly increased when fluoropyrimidine was coupled with cardiotoxic drugs such as anthracyclines, which has been approved as a risk for the occurrence of adverse cardiac events [233]. Therefore, when chemotherapy regimens containing anthracyclines are considered, close attention should be paid. In addition, our findings observed that cardiotoxicity induced by capecitabine was not significantly higher than 5-FU, although some previous studies showed a higher toxicity in capecitabine treatment [44,95]. The incidence of FAC differs by 5-FU administration (continuous infusion or bolus infusion), and it occurs in a cumulative dose-dependent manner [147], which was confirmed by our results. In view of the prominent cardiotoxicity of fluoropyrimidines, it is important to develop safe and efficacious new fluoropyrimidine drugs. TAS-102, a novel fluorouracil agent, is a promising safe option for patients. In addition, raltitrexed (Tomudex^®^) can also be used as an alternative to conventional fluorouracil drugs, with a lower incidence of FAC [240].

This study contains some limitations. The first is the heterogeneity at the study level. Although series subgroup analyses and meta-regression were performed to explore the source of heterogeneity further, they did not explain all the heterogeneity of the pooled effects. The clinical heterogeneity of the participants in the included studies could potentially have induced heterogeneity in the results of this meta-analysis. The attributes of the included studies could also have led to unstable results. For example, prospective RCT research can produce more robust conclusions in theory. Secondly, although all the reported cardiac AEs were diagnosed and graded based on the general NCI or WHO criteria, cardiotoxicity might have been underestimated in some studies due to the lack of cardiac-specific examinations. Finally, most of the included studies, especially the clinical trials, adopted strict inclusion criteria, limiting the generalizability of our results for patients outside the inclusion criteria (e.g., patients younger than 18 years or older than 70 years). Hence, real-word studies based on a large-scale population and robust analysis should be necessary to confirm our findings.

## 5. Conclusions

In conclusion, this meta-analysis systematically and comprehensively redefined the incidence and profile of FAC. FAC is not as rare as it seems, and involves a wide variety of related cardiac AEs, with cardiac ischemia and arrhythmia being the most common. The occurrence of cardiotoxicity varies among different publication decades, country/regions, genders, cancer types, and treatment details. Combination therapy, high cumulative dose, addition of anthracyclines, and pre-existing heart disease potentially increase the risk of FAC. This global overview of cardiotoxicity in fluoropyrimidine-based treatment may be used as a clinical reference in clinical practice for the management of cancer therapy.

## Figures and Tables

**Table 1:** Characteristics of the 206 studies included in this meta-analysis.

	No. of Studies (*n*)
**Year Published**	
Before 1980	1
1981 to 1990	7
1991 to 2000	63
2001 to 2010	77
2011 to 2020	57
After 2020	6
**Study Design**	
Retrospective	34
Prospective ^a^	177
Phase ii trial	81
Phase iii trial	34
**Population Age**	
No limitation	185
Older adults (>60 years)	6
NA	20
Gender	
Female only	67
Female and male	120
NA	24
**Tumor Type**	
Breast cancer	66
Colorectal cancer	56
Gastric cancer	15
Esophagus cancer	11
Head and neck cancer	11
Gastrointestinal cancer	5
Pancreatic cancer	4
Lung cancer ^b^	4
Anal cancer	4
Solid malignancies ^c^	28
Others ^d^	7
**Treatment Regimen ^e^**	
5-FU monotherapy	23
5-FU combined chemotherapy	114
5-FU based chemotherapy + targeted therapy	15
Capecitabine monotherapy	20
Capecitabine combined chemotherapy	23
Capecitabine based chemotherapy + targeted therapy	28
S-1 monotherapy or combined therapy	5
TAS 102 monotherapy	1
Fluoropyrimidines + radiotherapy	25
Fluoropyrimidines + immunotherapy	1
Mixed fluoropyrimidines ^f^	8
Fluoropyrimidines + targeted therapy + radiotherapy	1
**Treatment Attribute**	
Neoadjuvant	24
Adjuvant	27
For advanced/metastatic/or relapsed disease	58
First line	32
≥second line	8
**Methodological Quality ^g^**	
Good	61
Fair	150
Poor	0

Notes: NA, not available; ^a^, there were 177 prospective studies, of which 115 were clearly marked as clinical trials (81 phase II trials and 34 phase III trials), and the remaining 62 were not indicated as clinical trials; ^b^, fluoropyrimidine is no longer recommended for use in lung cancer, and the included four studies were all in the 1990s; ^c^, solid malignancies: including two or more tumor types, such as breast cancer, colorectal cancer, gastric cancer, head and neck cancer, and so on; ^d^, others: including renal cell carcinoma (*n* = 1), prostate cancer (*n* = 1), ovarian cancer (*n* = 1), hepatocellular carcinoma (*n* = 1), bladder cancer (*n* = 1), biliary tract cancer (*n* = 1), and adrenal cortical carcinoma (*n* = 1); ^e^, the data were calculated based on 265 arms from 211 included studies; ^f^, mixed fluoropyrimidines: does not specifically refer to one single drug, containing two or more fluorouracil drugs; ^g^, the methodological quality was assessed by the quality assessment tool of the National Institutes of Health (NIH).

**Table 2:** Characteristics of the 114 FAC-related deaths.

Cause of Death	N	(%)
Sudden cardiac arrest	33	28.95%
Myocardial infarction	31	27.19%
Heart failure	18	15.79%
Arrhythmias	16	14.04%
Complete atrioventricular block	4	3.51%
Ventricular fibrillation	1	0.88%
Arrhythmia (specific type NA)	11	9.65%
Pericarditis/pericardial effusion	2	1.75%
Cardiomyopathy	1	0.88%
NA *	13	11.4%

Notes: NA, not available; * Thirteen studies only reported deaths due to cardiotoxicity, but did not provide details about it.

**Table 3:** The results of multilevel meta-regression analysis.

Factors	Variables	β	Z Value	*p* Value	
Intrcpt	/	−6.648	−2.432	0.015	
Publication year	Continuous data	0.003	2.326	0.020	*
Age	All range	/	/	/	
	Elderly (≥60 years)	−0.013	−0.196	0.844	
Country/region	America	/	/	/	
	Asia	0.094	2.796	0.005	**
	Europe	0.001	0.023	0.982	
	Oceania	−0.072	−1.161	0.246	
	Multi: Europe/America/Oceania	0.052	1.302	0.193	
Gender	Female and male	/	/	/	
	Female only	−0.031	−0.546	0.585	
Pre-existing cardiac disorder (-) ^a^	Yes	/	/	/	
	No	0.060	2.865	0.004	**
Recent chemotherapy (-) ^b^	Yes	/	/	/	
	No	0.010	0.488	0.625	
Methodological quality ^c^	Fair	/	/	/	
	Good	−0.006	−0.246	0.806	
Study design	Retrospective	/	/	/	
	Prospective	0.063	2.258	0.024	*
Phase for trials	Phase ii	/	/	/	
	Phase iii	−0.026	−0.844	0.399	
Treatment attribute	Neoadjuvant	/	/	/	
	Adjuvant	0.098	2.5112	0.012	*
	For advanced/metastasis/relapse	0.162	3.768	0.000	***
Treatment line ^d^	First line	/	/	/	
	≥second line	0.023	0.368	0.713	
Tumor type	Esophagus cancer	/	/	/	
	Breast cancer	−0.079	−1.055	0.291	
	Colorectal cancer	−0.082	−1.703	0.089	-
	Gastric cancer	−0.142	−2.222	0.026	*
	Head and neck cancer	−0.082	−1.595	0.111	
	Others ^e^	−0.044	−0.854	0.393	
Regimen	5-FU mono	/	/	/	
	Capecitabine mono	0.060	0.972	0.331	
	5-FU-combined chemo	0.069	1.920	0.055	-
	Capecitabine-combined chemo	0.105	1.908	0.056	-
	5-FU-based chemo/target	0.149	2.021	0.043	*
	Capecitabine-based chemo/target	0.089	1.480	0.139	
	5-FU-based chemo/radiotherapy	−0.001	−0.014	0.989	
	Capecitabine-based chemo/radiotherapy	0.030	0.405	0.686	
Targeted agent ^f^	Lapatinib	/	/	/	
	Trastuzumab	0.049	0.742	0.458	
	Bevacizumab	0.016	0.225	0.822	
	Cetuximab	0.078	0.576	0.565	
	Sorafenib	0.017	0.115	0.908	
	Bevacizumab + cetuximab	0.035	0.385	0.700	
5-FU dosage	Bolus infusion for non-consecutive days	/	/	/	
	Bolus infusion for 3–5 consecutive days	0.088	0.825	0.409	
	Bolus infusion followed by continuous infusion	0.069	0.638	0.523	
	Continuous infusion d1,8 or d1	−0.033	−0.299	0.765	
	24 h continuous infusion	0.011	0.090	0.928	
	Continuous infusion for 3–5 consecutive days	0.114	1.059	0.290	
Capecitabine dosage	1500–2000 mg/m^2^ 14 days Q3w	/	/	/	
	2500/2510 mg/m^2^ 14 days Q3w	0.086	1.684	0.092	-
Combined with anthracycline ^g^	No	/	/	/	
	Yes	0.116	2.213	0.027	*

Notes: - *p* < 0.1, * *p* < 0.05, ** *p* < 0.01, *** *p* < 0.001; NA, not available; ^a^, pre-existing cardiac disorder (-): were patients with cardiotoxicity excluded or not? ^b^, recent chemotherapy (-): were patients with recent chemotherapy excluded or not? ^c^, the methodological quality was assessed by the quality assessment tool of the National Institutes of Health (NIH); ^d^, the treatment lines were only for metastasis/advanced/relapsed disease; ^e^, “others” including gastrointestinal cancer, pancreatic cancer, lung cancer, and anal cancer. ^f^, targeted agents combination therapy in breast cancer and colorectal cancer; ^g^, combination therapy with anthracycline in breast cancer.

## Data Availability

The data presented in this study are available upon request from the corresponding author.

## Supplementary Materials

The following supporting information can be downloaded at: https://www.mdpi.com/article/10.3390/ph16040510/s1. Supplementary Table S1: The retrieval scheme used for each database; Supplementary Table S2: The National Institutes of Health (NIH) quality assessment tools; Supplementary Table S3: List of the included 206 studies. Supplementary Figure S1: Number of articles published by year. Articles on anthracycline-associated cardiotoxicity have increased dramatically in the past two decades, and the cardiotoxicity of immune checkpoint inhibitors (ICIs) has also attracted much attention in the last 5 years. However, fluoropyrimidine-associated cardiotoxicity (FAC) has not received universal attention, and there is no obvious tendency of increase in the number of published articles; Supplementary Figure S2: Forest plot of the pooled incidence of all-grade FAC using a random-effects model; Supplementary Figure S3: Forest plot for the analysis of incidence of grade 3 or higher cardiac AEs using a random-effects model; Supplementary Figure S4: Forest plot for the analysis of incidence of grade 1 or grade 2 cardiac AEs using a random-effects model; Supplementary Figure S5: Forest plot for the subgroup analysis by 5-FU administration pattern (24 h continuous infusion, bolus infusion for non-consecutive days, bolus infusion followed by continuous infusion, bolus infusion for 3–5 consecutive days, continuous infusion d1,8 or d1, and continuous infusion for 3–5 consecutive days) using a random-effects model. A significant difference was detected between different 5-FU administration patterns (χ^2^ = 12.29, *p* = 0.03); Supplementary Figure S6: Forest plot for the subgroup analysis by cumulative 5-FU dose (≤1000, 1000–2000, 2000–3000, and >3000) using a random-effects model. A significant difference was detected between different cumulative 5-FU dosages (χ^2^ = 8.41, *p* = 0.04); Supplementary Figure S7: The bubble plots showed the results of univariable meta-regression of continuous data (female proportion, 5-FU dosage schedule, publication year, and median age). A. significant negative relationship between female proportion and the incidence of cardiotoxicities (Q = 8.59, *p* < 0.01); B. significant positive relationship between 5-FU dosage schedule and the incidence of cardiotoxicities (Q = 9.57, *p* < 0.01); C. the relationship between publication year and the incidence of cardiotoxicities (Q = 3.69, *p* = 0.058); D. the relationship between median age and the incidence of cardiotoxicities (Q = 3.33, *p* = 0.071); Supplementary Figure S8: Egger’s funnel plot for publication bias test of the main outcomes, and no evidence of significant publication bias was detected. A. incidence of all-grade FACs; B. incidence of grade 3 or higher FACs; C. incidence of grade 1–2 FACs; D. incidence of cardiac ischemia; E. incidence of arrhythmia; F. incidence of ECG changes; Supplementary Figure S9: Forest plot for the sensitivity analysis of main outcomes. Removal of individual studies one at a time did not materially alter the results. A. incidence of all-grade FACs; B. incidence of grade 3 or higher FACs; C. incidence of grade 1–2 FACs; D. incidence of cardiac ischemia; E. incidence of arrhythmia; F. incidence of ECG changes.

## Abbreviations

FAC	fluoropyrimidine-associated cardiotoxicity
AEs	adverse effects
LVEF	left ventricular ejection fraction

## References

[1] Lokich J. (1998). Infusional 5-FU: Historical evolution, rationale, and clinical experience. Oncology.

[2] Pfeiffer P., Yilmaz M., Möller S., Zitnjak D., Krogh M., Petersen L.N., Poulsen L., Winther S.B., Thomsen K.G., Qvortrup C. (2020). TAS-102 with or without bevacizumab in patients with chemorefractory metastatic colorectal cancer: An investigator-initiated, open-label, randomised, phase 2 trial. Lancet Oncol..

[3] Bedard P.L., Hyman D.M., Davids M.S., Siu L.L. (2020). Small molecules, big impact: 20 years of targeted therapy in oncology. Lancet.

[4] Riley R.S., June C.H., Langer R., Mitchell M.J. (2019). Delivery technologies for cancer immunotherapy. Nat. Rev. Drug Discov..

[5] Parsons H.A., Burstein H.J. (2021). Adjuvant Capecitabine in Triple-Negative Breast Cancer: New Strategies for Tailoring Treatment Recommendations. JAMA.

[6] Deac A.L., Burz C.C., Bocsan I.C., Buzoianu A.D. (2020). Fluoropyrimidine-induced cardiotoxicity. World J. Clin. Oncol..

[7] Depetris I., Marino D., Bonzano A., Cagnazzo C., Filippi R., Aglietta M., Leone F. (2018). Fluoropyrimidine-induced cardiotoxicity. Crit. Rev. Oncol. Hematol..

[8] Koutsoukis A., Ntalianis A., Repasos E., Kastritis E., Dimopoulos M.A., Paraskevaidis I. (2018). Cardio-oncology: A Focus on Cardiotoxicity. Eur. Cardiol..

[9] Lenneman C.G., Sawyer D.B. (2016). Cardio-Oncology: An Update on Cardiotoxicity of Cancer-Related Treatment. Circ. Res..

[10] Curigliano G., Cardinale D., Dent S., Criscitiello C., Aseyev O., Lenihan D., Cipolla C.M. (2016). Cardiotoxicity of anticancer treatments: Epidemiology, detection, and management. CA Cancer J. Clin..

[11] Rosa G.M., Gigli L., Tagliasacchi M.I., Di Iorio C., Carbone F., Nencioni A., Montecucco F., Brunelli C. (2016). Update on cardiotoxicity of anti-cancer treatments. Eur. J. Clin. Investig..

[12] Lestuzzi C., Tartuferi L., Viel E., Buonadonna A., Vaccher E., Berretta M. (2020). Fluoropyrimidine-Associated Cardiotoxicity: Probably Not So Rare as It Seems. Oncologist.

[13] Polk A., Vaage-Nilsen M., Vistisen K., Nielsen D.L. (2013). Cardiotoxicity in cancer patients treated with 5-fluorouracil or capecitabine: A systematic review of incidence, manifestations and predisposing factors. Cancer Treat. Rev..

[14] Pai V.B., Nahata M.C. (2000). Cardiotoxicity of chemotherapeutic agents: Incidence, treatment and prevention. Drug Saf..

[15] Van Cutsem E., Hoff P.M., Blum J.L., Abt M., Osterwalder B. (2002). Incidence of cardiotoxicity with the oral fluoropyrimidine capecitabine is typical of that reported with 5-fluorouracil. Ann. Oncol..

[16] Liberati A., Altman D.G., Tetzlaff J., Mulrow C., Gotzsche P.C., Ioannidis J.P.A., Clarke M., Devereaux P.J., Kleijnen J., Moher D. (2009). The PRISMA statement for reporting systematic reviews and meta-analyses of studies that evaluate healthcare interventions: Explanation and elaboration. BMJ.

[17] International Prospective Register of Systematic Reviews. https://www.crd.york.ac.uk/prospero.

[18] Study Quality Assessment Tools. https://www.nhlbi.nih.gov/health-topics/study-quality-assessment-tools.

[19] Wang F., Sun G.P., Zou Y.F. (2013). Meat consumption and risk of lung cancer: Evidence from observational studies. Ann. Oncol..

[20] Zou Y., Zheng S., Deng X., Yang A., Kong Y., Kohansal M., Hu X., Xie X. (2020). Diagnostic and prognostic value of circular RNA CDR1as/ciRS-7 for solid tumours: A systematic review and meta-analysis. J. Cell Mol. Med..

[21] Kachnic L.A., Winter K.A., Myerson R.J., Goodyear M.D., Abitbol A.A., Streeter O.E., Augspurger M.E., Schefter T.E., Katz A.W., Fisher B.J. (2022). Long-Term Outcomes of NRG Oncology/RTOG 0529: A Phase 2 Evaluation of Dose-Painted Intensity Modulated Radiation Therapy in Combination With 5-Fluorouracil and Mitomycin-C for the Reduction of Acute Morbidity in Anal Canal Cancer. Int J Radiat Oncol Biol Phys..

[22] Yamaguchi K., Minashi K., Sakai D., Nishina T., Omuro Y., Tsuda M., Iwagami S., Kawakami H., Esaki T., Sugimoto N. (2022). Phase IIb study of pembrolizumab combined with S-1 + oxaliplatin or S-1 + cisplatin as first-line chemotherapy for gastric cancer. Cancer Sci..

[23] Conroy T., Bosset J.F., Etienne P.L., Rio E., Francois É., Mesgouez-Nebout N., Vendrely V., Artignan X., Bouché O., Gargot D. (2021). Unicancer Gastrointestinal Group and Partenariat de Recherche en Oncologie Digestive (PRODIGE) Group. Neoadjuvant chemotherapy with FOLFIRINOX and preoperative chemoradiotherapy for patients with locally advanced rectal cancer (UNICANCER-PRODIGE 23): A multicentre, randomised, open-label, phase 3 trial. Lancet Oncol..

[24] Hofheinz R.D., Hegewisch-Becker S., Kunzmann V., Thuss-Patience P., Fuchs M., Homann N., Graeven U., Schulte N., Merx K., Pohl M. (2021). Trastuzumab in combination with 5-fluorouracil, leucovorin, oxaliplatin and docetaxel as perioperative treatment for patients with human epidermal growth factor receptor 2-positive locally advanced esophagogastric adenocarcinoma: A phase II trial of the Arbeitsgemeinschaft Internistische Onkologie Gastric Cancer Study Group. Int. J. Cancer.

[25] Meyerhardt J.A., Shi Q., Fuchs C.S., Meyer J., Niedzwiecki D., Zemla T., Kumthekar P., Guthrie K.A., Couture F., Kuebler P. (2021). Effect of Celecoxib vs Placebo Added to Standard Adjuvant Therapy on Disease-Free Survival Among Patients with Stage III Colon Cancer: The CALGB/SWOG 80702 (Alliance) Randomized Clinical Trial. JAMA.

[26] Mayer I.A., Zhao F., Arteaga C.L., Symmans W.F., Park B.H., Burnette B.L., Tevaarwerk A.J., Garcia S.F., Smith K.L., Makower D.F. (2021). Randomized Phase III Postoperative Trial of Platinum-Based Chemotherapy Versus Capecitabine in Patients with Residual Triple-Negative Breast Cancer Following Neoadjuvant Chemotherapy: ECOG-ACRIN EA1131. J. Clin. Oncol..

[27] Chakravarthy A.B., Zhao F., Meropol N.J., Flynn P.J., Wagner L.I., Sloan J., Diasio R.B., Mitchell E.P., Catalano P., Giantonio B.J. (2020). Intergroup Randomized Phase III Study of Postoperative Oxaliplatin, 5-Fluorouracil, and Leucovorin Versus Oxaliplatin, 5-Fluorouracil, Leucovorin, and Bevacizumab for Patients with Stage II or III Rectal Cancer Receiving Preoperative Chemoradiation: A Trial of the ECOG-ACRIN Research Group (E5204). Oncologist.

[28] Dyhl-Polk A., Schou M., Vistisen K.K., Sillesen A.S., Serup-Hansen E., Faber J., Klausen T.W., Bojesen S.E., Vaage-Nilsen M., Nielsen D.L. (2021). Myocardial Ischemia Induced by5-Fluorouracil: A Prospective Electrocardiographic and Cardiac Biomarker Study. Oncologist.

[29] Aimar G., Lombardi P., Quarà V., Milanesio M.C., Crespi V., Farinea G., Fenocchio E. (2020). Predictive factor of cardiotoxicity in fluoropyrimidine-treated colorectal cancer patients: Interim analysis of the prospective observational CHECKPOINT trial. Ann. Oncol..

[30] Osterlund P.J., Kinos S., Halonen P., Soveri L.M., Kwakman J.J., Salminen T., McDermott R.S., Pfeiffer P., Heerva E., Liposits G. (2020). Feasibility of switching to S-1 upon other fluoropyrimidine-related cardiotoxicity during chemotherapy for solid tumors. J. Clin. Oncol..

[31] Rajendra A., Noronha V., Patil V.M., Joshi A., Menon N.S., Prabhash K. (2020). Incidence of 5-fluorouracil related in cardiotoxicity in patients with head and neck cancer. J. Clin. Oncol..

[32] Delaloge S., Piccart M., Rutgers E., Litière S., van’t Veer L.J., van den Berkmortel F., Brain E., Dudek-Peric A., Gil-Gil M., Gomez P. (2020). MINDACT investigators and the TRANSBIG Consortium: Standard Anthracycline Based Versus Docetaxel-Capecitabine in Early High Clinical and/or Genomic Risk Breast Cancer in the EORTC 10041/BIG 3-04 MINDACT Phase III Trial. J. Clin. Oncol..

[33] Grierson P., Teague A., Suresh R., Lim K.H., Amin M., Pedersen K., Tan B., Huffman J., Boice N., Du L. (2020). Phase Ib/II study combining tosedostat with capecitabine in patients with advanced pancreatic adenocarcinoma. J. Gastrointest. Oncol..

[34] Dyhl-Polk A., Vaage-Nilsen M., Schou M., Vistisen K.K., Lund C.M., Kümler T., Appel J.M., Nielsen D.L. (2020). Incidence and risk markers of 5-fluorouracil and capecitabine cardiotoxicity in patients with colorectal cancer. Acta Oncol..

[35] Gibson M.K., Catalano P., Kleinberg L.R., Staley C.A., Montgomery E.A., Jimeno A., Song W.F., Mulcahy M.F., Leichman L.P., Benson A.B. (2019). Phase II Study of Preoperative Chemoradiotherapy with Oxaliplatin, Infusional 5-Fluorouracil, and Cetuximab Followed by Postoperative Docetaxel and Cetuximab in Patients with Adenocarcinoma of the Esophagus: A Trial of the ECOG-ACRIN Cancer Research Group (E2205). Oncologist.

[36] Raber I., Warack S., Kanduri J., Pribish A., Godishala A., Abovich A., Orbite A., Dommaraju S., Frazer M., Peters M.L. (2019). Fluoropyrimidine-Associated Cardiotoxicity: A Retrospective Case-Control Study. Oncologist.

[37] Shanmuganathan J., Kragholm K., Tayal B.T., Poulsen L.P., El-Galaly T., Fosbol E., Gislason G.G., Kober L.K., Sogaard P.S., Torp-Pedersen C. (2019). Higher risk of myocardial infarction in the first year following 5-fluorouracil treatment. Eur. Heart J..

[38] Lombardi P., Aimar G., Depetris I., Bonzano A., Leone F. (2019). Fluoropyrimidine-induced cardiotoxicity in colorectal cancer patients: Preliminary data from the prospective observational CHECKPOINT trial (NCT02665312). Ann. Oncol..

[39] Jin X., Bai Y., Gao L., Wu S. (2019). Incidence of and risk factors for cardiotoxicity after fluorouracil-based chemotherapy in locally advanced or metastatic gastric cancer patients. Cancer Chemoth. Pharm..

[40] Rivera F., Romero C., Jimenez-Fonseca P., Izquierdo-Manuel M., Salud A., Martínez E., Jorge M., Arrazubi V., Méndez J.C., García-Alfonso P. (2019). Phase II study to evaluate the efficacy of Trastuzumab in combination with Capecitabine and Oxaliplatin in first-line treatment of HER2-positive advanced gastric cancer: HERXO trial. Cancer Chemoth. Pharm..

[41] Primrose J.N., Fox R.P., Palmer D.H., Malik H.Z., Prasad R., Mirza D., Anthony A., Corrie P., Falk S., Finch-Jones M. (2019). Capecitabine compared with observation in resected biliary tract cancer (BILCAP): A randomised, controlled, multicentre, phase 3 study. Lancet Oncol..

[42] Abdel-Rahman O. (2019). 5-Fluorouracil- related Cardiotoxicity, Findings from Five Randomized Studies of 5-Fluorouracil-based Regimens in Metastatic Colorectal Cancer. Clin. Color. Cancer.

[43] Hayashi Y., Iijima H., Isohashi F., Tsujii Y., Fujinaga T., Nagai K., Yoshii S., Sakatani A., Hiyama S., Shinzaki S. (2019). The heart’s exposure to radiation increases the risk of cardiac toxicity after chemoradiotherapy for superficial esophageal cancer: A retrospective cohort study. Bmc Cancer.

[44] Peng J., Dong C., Wang C., Li W., Yu H., Zhang M., Zhao Q., Zhu B., Zhang J., Li W. (2018). Cardiotoxicity of 5-fluorouracil and capecitabine in Chinese patients: A prospective study. Cancer Commun..

[45] Chen E.Y., Blanke C.D., Haller D.G., Benson A.B., Dragovich T., Lenz H.J., Robles C., Li H., Mori M., Mattek N. (2018). A Phase II Study of Celecoxib with Irinotecan, 5-Fluorouracil, and Leucovorin in Patients With Previously Untreated Advanced or Metastatic Colorectal Cancer. Am J. Clin. Oncol..

[46] Yang H., Sui Y., Guo X., Tan X., Li Y., Wang M. (2018). Endostar continuous intravenous infusion combined with S-1 and oxaliplatin chemotherapy could be effective in treating liver metastasis from gastric cancer. J. Cancer Res. Ther..

[47] Alderson D., Cunningham D., Nankivell M., Blazeby J.M., Griffin S.M., Crellin A., Grabsch H.I., Langer R., Pritchard S., Okines A. (2017). Neoadjuvant cisplatin and fluorouracil versus epirubicin, cisplatin, and capecitabine followed by resection in patients with oesophageal adenocarcinoma (UK MRC OE05): An open-label, randomised phase 3 trial. Lancet Oncol..

[48] Kwakman J.J., Simkens L.H., Mol L., Kok W.E., Koopman M., Punt C.J. (2017). Incidence of capecitabine-related cardiotoxicity in different treatment schedules of metastatic colorectal cancer: Incidence of capecitabine-related cardiotoxicity in different treatment schedules of metastatic colorectal cancer: A retrospective analysis of the CAIRO studies of the Dutch Colorectal Cancer Group. Eur. J. Cancer.

[49] Turan T., Agac M.T., Aykan A.C., Kul S., Akyüz A.R., Gökdeniz T., Gül İ., Cengiz E., Boyacı F., Erkan H. (2017). Usefulness of Heart-Type Fatty Acid-Binding Protein and Myocardial Performance Index for Early Detection of 5-Fluorouracil Cardiotoxicity. Angiology.

[50] Leicher L.W., de Graaf J.C., Coers W., Tascilar M., de Groot J.W. (2017). Tolerability of Capecitabine Monotherapy in Metastatic Colorectal Cancer: A Real-World Study. Drug In R&D.

[51] Zhang P., Sun T., Zhang Q., Yuan Z., Jiang Z., Wang X.J., Cui S., Teng Y., Hu X.C., Yang J. (2017). BG01-1323L study group. Utidelone plus capecitabine versus capecitabine alone for heavily pretreated metastatic breast cancer refractory to anthracyclines and taxanes: A multicentre, open-label, superiority, phase 3, randomised controlled trial. Lancet Oncol..

[52] Harbeck N., Saupe S., Jäger E., Schmidt M., Kreienberg R., Müller L., Otremba B.J., Waldenmaier D., Dorn J., Warm M. (2017). PELICAN Investigators. A randomized phase III study evaluating pegylated liposomal doxorubicin versus capecitabine as first-line therapy for metastatic breast cancer: Results of the PELICAN study. Breast Cancer Res. Tr..

[53] Kerr R.S., Love S., Segelov E., Johnstone E., Falcon B., Hewett P., Weaver A., Church D., Scudder C., Pearson S. (2016). Adjuvant capecitabine plus bevacizumab versus capecitabine alone in patients with colorectal cancer (QUASAR 2): An open-label, randomised phase 3 trial. Lancet Oncol..

[54] Lestuzzi C., Tartuferi L., Viel E. (2016). Incidence of capecitabine cardiac toxicity at rest and under effort: A prospective study. Eur. Heart J..

[55] Winther S.B., Zubcevic K., Qvortrup C., Vestermark L.W., Jensen H.A., Krogh M., Sorbye H., Pfeiffer P. (2016). Academy of Geriatric Cancer Research (AgeCare). Experience with S-1 in older Caucasian patients with metastatic colorectal cancer (mCRC): Findings from an observational chart review. Acta Oncol..

[56] Polk A., Shahmarvand N., Vistisen K., Vaage-Nilsen M., Larsen F.O., Schou M., Nielsen D.L. (2016). Incidence and risk factors for capecitabine-induced symptomatic cardiotoxicity: A retrospective study of 452 consecutive patients with metastatic breast cancer. Bmj Open.

[57] Mayer R.J., Van Cutsem E., Falcone A., Yoshino T., Garcia-Carbonero R., Mizunuma N., Yamazaki K., Shimada Y., Tabernero J., Komatsu Y. (2015). Randomized Trial of TAS-102 for Refractory Metastatic Colorectal Cancer. N. Engl. J. Med..

[58] van der Sluis P.C., Ubink I., van der Horst S., Boonstra J.J., Voest E.E., Ruurda J.P., Borel Rinkes I.H., Wiezer M.J., Schipper M.E., Siersema P.D. (2015). Safety, Efficacy, and Long-Term Follow-Up Evaluation of Perioperative Epirubicin, Cisplatin, and Capecitabine Chemotherapy in Esophageal Resection for Adenocarcinoma. Ann. Surg. Oncol..

[59] Lestuzzi C., Vaccher E., Talamini R., Lleshi A., Meneguzzo N., Viel E., Scalone S., Tartuferi L., Buonadonna A., Ejiofor L. (2014). Effort myocardial ischemia during chemotherapy with 5-fluorouracil: An underestimated risk. Ann. Oncol..

[60] Tonyali O., Benekli M., Berk V., Coskun U., Ozkan M., Yildiz R., Ucgul E., Sevinc A., Uncu D., Demirci U. (2013). Efficacy and toxicity of Trastuzumab and Paclitaxel plus Capecitabine in the first-line treatment of HER2-positive metastatic breast cancer. J. Cancer Res. Clin..

[61] Okines A.F., Langley R.E., Thompson L.C., Stenning S.P., Stevenson L., Falk S., Seymour M., Coxon F., Middleton G.W., Smith D. (2013). Bevacizumab with peri-operative epirubicin, cisplatin and capecitabine (ECX) in localised gastro-oesophageal adenocarcinoma: A safety report. Ann. Oncol..

[62] Uygun K., Bilici A., Kaya S., Oven Ustaalioglu B.B., Yildiz R., Temiz S., Seker M., Aksu G., Cabuk D., Gumus M. (2013). Xeliri Plus Bevacizumab Compared with Folfiri Plus Bevacizumab as First-Line Setting in Patients with Metastatic Colorectal Cancer: Experiences at Two-Institutions. Asian Pac. J. Cancer Prev..

[63] Mackey J.R., Martin M., Pienkowski T., Rolski J., Guastalla J.P., Sami A., Glaspy J., Juhos E., Wardley A., Fornander T. (2013). Adjuvant docetaxel, doxorubicin, and cyclophosphamide in node-positive breast cancer: 10-year follow-up of the phase 3 randomised BCIRG 001 trial. Lancet Oncol..

[64] Souglakos J., Ziras N., Kakolyris S., Boukovinas I., Kentepozidis N., Makrantonakis P., Xynogalos S., Christophyllakis C.H., Kouroussis C.H., Vamvakas L. (2012). Randomised phase-II trial of CAPIRI (capecitabine, irinotecan) plus bevacizumab vs FOLFIRI (folinic acid, 5-fluorouracil, irinotecan) plus bevacizumab as first-line treatment of patients with unresectable/metastatic colorectal cancer (mCRC). Br. J. Cancer.

[65] Cetin B., Benekli M., Oksuzoglu B., Koral L., Ulas A., Dane F., Turker I., Kaplan M.A., Koca D., Boruban C. (2012). Lapatinib plus Capecitabine for Brain Metastases in Patients with Human Epidermal Growth Factor Receptor 2-Positive Advanced Breast Cancer: A Review of the Anatolian Society of Medical Oncology (ASMO) Experience. Onkologie.

[66] Lang I., Inbar M.J., Kahán Z., Greil R., Beslija S., Stemmer S.M., Kaufman B., Zvirbule Z., Steger G.G., Messinger D. (2012). Safety results from a phase III study (TURANDOT trial by CECOG) of first-line bevacizumab in combination with capecitabine or paclitaxel for HER-2-negative locally recurrent or metastatic breast cancer. Eur. J. Cancer.

[67] Nishimura Y., Hiraoka M., Koike R., Nakamatsu K., Itasaka S., Kawamura M., Negoro Y., Araki N., Ishikawa H., Fujii T. (2012). Long-term Follow-up of a Randomized Phase II Study of Cisplatin/5-FU Concurrent Chemoradiotherapy for Esophageal Cancer (KROSG0101/JROSG021). Jpn. J. Clin. Oncol..

[68] Cen P., Liu C., Du X.L. (2012). Comparison of toxicity profiles of fluorouracil versus oxaliplatin regimens in a large population-based cohort of elderly patients with colorectal cancer. Ann. Oncol..

[69] Khan M.A., Masood N., Husain N., Ahmad B., Aziz T., Naeem A. (2012). A retrospective study of cardiotoxicities induced by 5-Fluouracil (5-FU) and 5-FU based chemotherapy regimens in Pakistani adult cancer patients at Shaukat Khanum Memorial Cancer Hospital & Research Center. J. Pak. Med. Assoc..

[70] Martín M., Makhson A., Gligorov J., Lichinitser M., Lluch A., Semiglazov V., Scotto N., Mitchell L., Tjulandin S. (2012). Phase II Study of Bevacizumab in Combination with Trastuzumab and Capecitabine as First-Line Treatment for HER-2-positive Locally Recurrent or Metastatic Breast Cancer. Oncologist.

[71] Petrini I., Lencioni M., Ricasoli M., Iannopollo M., Orlandini C., Oliveri F., Bartolozzi C., Ricci S. (2012). Phase II trial of sorafenib in combination with 5-fluorouracil infusion in advanced hepatocellular carcinoma. Cancer Chemoth. Pharm..

[72] Lopez L., Montenegro P.C. (2012). Cardiotoxicity of 5-fluorouracil in patients with gastrointestinal cancer. J. Clin. Oncol..

[73] Dipetrillo T., Pricolo V., Lagares-Garcia J., Vrees M., Klipfel A., Cataldo T., Sikov W., McNulty B., Shipley J., Anderson E. (2012). Neoadjuvant bevacizumab, oxaliplatin, 5-fluorouracil, and radiation for rectal cancer. Int. J. Radiat. Oncol..

[74] Greil R., Borštnar S., Petráková K., Marcou Y., Pikiel J., Wojtukiewicz M.Z., Koza I., Steger G.G., Linn M., Das Gupta A. (2012). Combination Therapy of Lapatinib and Capecitabine for ErbB2-Positive Metastatic or Locally Advanced Breast Cancer: Results from the Lapatinib Expanded Access Program (LEAP) in Central and Eastern Europe. Onkologie.

[75] Robert N.J., Diéras V., Glaspy J., Brufsky A.M., Bondarenko I., Lipatov O.N., Perez E.A., Yardley D.A., Chan S.Y., Zhou X. (2011). RIBBON-1: Randomized, Double-Blind, Placebo-Controlled, Phase III Trial of Chemotherapy with or Without Bevacizumab for First-Line Treatment of Human Epidermal Growth Factor Receptor 2–Negative, Locally Recurrent or Metastatic Breast Cancer. J. Clin. Oncol..

[76] Koca D., Salman T., Unek I.T., Oztop I., Ellidokuz H., Eren M., Yilmaz U. (2011). Clinical and Electrocardiography Changes in Patients Treated with Capecitabine. Chemotherapy.

[77] Jensen S.A., Hasbak P., Mortensen J., Sørensen J.B. (2010). Fluorouracil Induces Myocardial Ischemia with Increases of Plasma Brain Natriuretic Peptide and Lactic Acid but Without Dysfunction of Left Ventricle. J. Clin. Oncol..

[78] Kato K., Muro K., Minashi K., Ohtsu A., Ishikura S., Boku N., Takiuchi H., Komatsu Y., Miyata Y., Fukuda H. (2011). Phase II study of chemoradiotherapy with 5-fluorouracil and cisplatin for Stage II-III esophageal squamous cell carcinoma: JCOG trial (JCOG 9906). Int. J. Radiat. Oncol..

[79] Wildiers H., Neven P., Christiaens M.R., Squifflet P., Amant F., Weltens C., Smeets A., van Limbergen E., Debrock G., Renard V. (2011). Neoadjuvant capecitabine and docetaxel (plus trastuzumab): An effective non-anthracycline-based chemotherapy regimen for patients with locally advanced breast cancer. Ann. Oncol..

[80] Torrisi R., Cardillo A., Cancello G., Dellapasqua S., Balduzzi A., Ghisini R., Luini A., Veronesi P., Viale G., Goldhirsch A. (2010). Phase II Trial of Combination of Pegylated Liposomal Doxorubicin, Cisplatin, and Infusional 5-Fluorouracil (CCF) Plus Trastuzumab as Preoperative Treatment for Locally Advanced and Inflammatory Breast Cancer. Clin. Breast Cancer.

[81] Masi G., Loupakis F., Salvatore L., Fornaro L., Cremolini C., Cupini S., Ciarlo A., Del Monte F., Cortesi E., Amoroso D. (2010). Bevacizumab with FOLFOXIRI (irinotecan, oxaliplatin, fluorouracil, and folinate) as first-line treatment for metastatic colorectal cancer: A phase 2 trial. Lancet Oncol..

[82] Michalaki V., Fotiou S., Gennatas S., Gennatas C. (2010). Trastuzumab plus Capecitabine and Docetaxel as First-line Therapy for HER2-positive Metastatic Breast Cancer: Phase II Results. Anticancer Res..

[83] Chua Y.J., Barbachano Y., Cunningham D., Oates J.R., Brown G., Wotherspoon A., Tait D., Massey A., Tebbutt N.C., Chau I. (2010). Neoadjuvant capecitabine and oxaliplatin before chemoradiotherapy and total mesorectal excision in MRI-defined poor-risk rectal cancer: A phase 2 trial. Lancet Oncol..

[84] Wardley A.M., Pivot X., Morales-Vasquez F., Zetina L.M., de Fátima Dias Gaui M., Reyes D.O., Jassem J., Barton C., Button P., Hersberger V. (2010). Randomized Phase II Trial of First-Line Trastuzumab Plus Docetaxel and Capecitabine Compared with Trastuzumab Plus Docetaxel in HER2-Positive Metastatic Breast Cancer. J. Clin. Oncol..

[85] Baur M., Horvath M., Stättner S., Schratter-Sehn A., Horvath B., Sellner F., Hudec M., Klimpfinger M., Dittrich C., Karner J. (2010). Chemoradiotherapy with 5-fluorouracil/leucovorin, surgery and adjuvant chemotherapy for locally advanced rectal cancer. Oncol. Lett..

[86] Hu W., Shi J., Sheng Y., Li L., Su D., Wang C.K. (2010). Clinical Study of Adjuvant Capecitabine Monotherapy in Chinese Elderly Patients (Aged 55–70) with Stage IIa Breast Cancer. Onkologie.

[87] Martín M., Seguí M.A., Antón A., Ruiz A., Ramos M., Adrover E., Aranda I., Rodríguez-Lescure A., Grosse R., Calvo L. (2010). Adjuvant docetaxel for high-risk, node-negative breast cancer. N. Engl. J. Med..

[88] Joensuu H., Bono P., Kataja V., Alanko T., Kokko R., Asola R., Utriainen T., Turpeenniemi-Hujanen T., Jyrkkiö S., Möykkynen K. (2009). Fluorouracil, Epirubicin, and Cyclophosphamide with Either Docetaxel or Vinorelbine, With or Without Trastuzumab, As Adjuvant Treatments of Breast Cancer: Final Results of the FinHer Trial. J. Clin. Oncol..

[89] Osawa S., Furuta T., Sugimoto K., Kosugi T., Terai T., Yamade M., Takayanagi Y., Nishino M., Hamaya Y., Kodaira C. (2009). Prospective study of daily low-dose nedaplatin and continuous 5-fluorouracil infusion combined with radiation for the treatment of esophageal squamous cell carcinoma. Bmc Cancer.

[90] Pisano C., Morabito A., Sorio R., Breda E., Lauria R., Gebbia V., Scaltriti L., Scalone S., Zagonel V., Greggi S. (2009). A phase II study of capecitabine in the treatment of ovarian cancer resistant or refractory to platinum therapy: A multicentre Italian trial in ovarian cancer (MITO-6) trial. Cancer Chemoth. Pharm..

[91] Bathe O.F., Ernst S., Sutherland F.R., Dixon E., Butts C., Bigam D., Holland D., Porter G.A., Koppel J., Dowden S. (2019). A phase II experience with neoadjuvant irinotecan (CPT-II), 5-fluorouracil (5-FU) and leucovorin (LV) for colorectal liver metastases. Bmc Cancer.

[92] Skof E., Rebersek M., Hlebanja Z., Ocvirk J. (2009). Capecitabine plus Irinotecan (XELIRI regimen) compared to 5-FU/LV plus Irinotecan (FOLFIRI regimen) as neoadjuvant treatment for patients with unresectable liver-only metastases of metastatic colorectal cancer: A randomised prospective phase II trial. Bmc Cancer.

[93] Giuliani F., Romito S., Maiello E., Capobianco A., Carrozza F., Nugnes I., Misino A., Valerio M.R., Manzione L., Colucci G. (2008). Epirubicin, taxotere and fluorouracil modulated by folinic acid in the treatment of advanced gastric cancer: A phase II study of the Gruppo Oncologico dell’ Italia Meridionale (GOIM). EJC Suppl..

[94] Ardavanis A., Kountourakis P., Mantzaris I., Malliou S., Doufexis D., Sykoutri D., Fragos I., Rigatos G. (2008). Bevacizumab Added to the Irinotecan and Capecitabine Combination for Advanced Colorectal Cancer: A Well-tolerated, Active and Convenient Regimen. Anticancer Res..

[95] Kosmas C., Kallistratos M.S., Kopterides P., Syrios J., Skopelitis H., Mylonakis N., Karabelis A., Tsavaris N. (2008). Cardiotoxicity of fluoropyrimidines in different schedules of administration: A prospective study. J. Cancer Res. Clin..

[96] Rothenberg M.L., Cox J.V., Butts C., Navarro M., Bang Y.J., Goel R., Gollins S., Siu L.L., Laguerre S., Cunningham D. (2008). Capecitabine plus oxaliplatin (XELOX) versus 5-fluorouracil/folinic acid plus oxaliplatin (FOLFOX-4) as second-line therapy in metastatic colorectal cancer: A randomized phase III noninferiority study. Ann. Oncol..

[97] Yamamoto D., Iwase S., Kitamura K., Odagiri H., Yamamoto C., Nagumo Y. (2008). A phase II study of trastuzumab and capecitabine for patients with HER2-overexpressing metastatic breast cancer: Japan Breast Cancer Research Network (JBCRN) 00 Trial. Cancer Chemoth. Pharm..

[98] Natoli C., Cianchetti E., Tinari N., Angelucci D., Grassadonia A., Zilli M., Ficorella C., Ricevuto E., Grossi S., De Tursi M. (2007). A phase II study of dose-dense epirubicin plus cyclophosphamide followed by docetaxel plus capecitabine and pegfilgrastim support as preoperative therapy for patients with stage II, IIIA breast cancer. Ann. Oncol..

[99] Neri B., Pantaleo P., Giommoni E., Grifoni R., Paoletti C., Rotella V., Pantalone D., Taddei A., Mercatelli A., Tonelli P. (2007). Oxaliplatin, 5-fluorouracil/leucovorin and epirubicin as first-line treatment in advanced gastric carcinoma: A phase II study. Brit. J. Cancer.

[100] Machiels J.P., Sempoux C., Scalliet P., Coche J.C., Humblet Y., Van Cutsem E., Kerger J., Canon J.L., Peeters M., Aydin S. (2007). Phase I/II study of preoperative cetuximab, capecitabine, and external beam radiotherapy in patients with rectal cancer. Ann. Oncol..

[101] Sasamoto R., Sakai K., Inakoshi H., Sueyama H., Saito M., Sugita T., Tsuchida E., Ito T., Matsumoto Y., Yamanoi T. (2007). Long-term results of chemoradiotherapy for locally advanced esophageal cancer, using daily low-dose 5-fluorouracil and cis-diammine-dichloroplatinum (CDDP). Int J. Clin. Oncol..

[102] Buzdar A.U., Valero V., Ibrahim N.K., Francis D., Broglio K.R., Theriault R.L., Pusztai L., Green M.C., Singletary S.E., Hunt K.K. (2007). Neoadjuvant therapy with paclitaxel followed by 5-fluorouracil, epirubicin, and cyclophosphamide chemotherapy and concurrent trastuzumab in human epidermal growth factor receptor 2-positive operable breast cancer: An update of the initial randomized study population and data of additional patients treated with the same regimen. Clin. Cancer Res..

[103] Yilmaz U., Oztop I., Ciloglu A., Okan T., Tekin U., Yaren A., Somali I., Alacacioglu A., Kirimli O. (2007). 5-Fluorouracil increases the number and complexity of premature complexes in the heart: A prospective study using ambulatory ECG monitoring. Int. J. Clin Pract.

[104] Garg M.K., Zhao F., Sparano J.A., Palefsky J., Whittington R., Mitchell E.P., Mulcahy M.F., Armstrong K.I., Nabbout N.H., Kalnicki S. (2017). Cetuximab Plus Chemoradiotherapy in Immunocompetent Patients with Anal Carcinoma: A Phase II Eastern Cooperative Oncology Group–American College of Radiology Imaging Network Cancer Research Group Trial (E3205). J. Clin. Oncol..

[105] Rossi D., Alessandroni P., Catalano V., Giordani P., Fedeli S.L., Fedeli A., Baldelli A.M., Casadei V., Ceccolini M., Catalano G. (2007). Safety Profile and Activity of Lower Capecitabine Dose in Patients with Metastatic Breast Cancer. Clin. Breast Cancer.

[106] Emmanouilides C., Sfakiotaki G., Androulakis N., Kalbakis K., Christophylakis C., Kalykaki A., Vamvakas L., Kotsakis A., Agelaki S., Diamandidou E. (2007). Front-line Bevacizumab in combination with Oxaliplatin, Leucovorin and 5-Fluorouracil (FOLFOX) in patients with metastatic colorectal cancer: A multicenter phase II study. Bmc Cancer.

[107] Geyer C.E., Forster J., Lindquist D., Chan S., Romieu C.G., Pienkowski T., Jagiello-Gruszfeld A., Crown J., Chan A., Kaufman B. (2006). Lapatinib plus capecitabine for HER2-positive advanced breast cancer. N. Engl. J. Med..

[108] Mambrini A., Sanguinetti F., Pacetti P., Caudana R., Iacono C., Guglielmi A., Guadagni S., Del Freo A., Fiorentini G., Cantore M l. (2006). Intra-arterial infusion of 5-fluorouracil, leucovorin, epirubicin and carboplatin (FLEC regimen) in unresectable pancreatic cancer: Results of a ten-year experience. In Vivo.

[109] Thatai L.C., Vishnubhotla P., Biernat L., Flaherty L., LoRusso P., Simon M., Stephens D., Vereeke K., Abrams J., Bouwman D. (2006). A phase II study of docetaxel, doxorubicin, and infusional 5-fluorouracil in the treatment of patients with locally advanced breast cancer. Am J. Clin. Oncol.-Cancer.

[110] Koopman M., Antonini N.F., Douma J., Wals J., Honkoop A.H., Erdkamp F.L., de Jong R.S., Rodenburg C.J., Vreugdenhil G., Akkermans-Vogelaar J.M. (2006). Randomised study of sequential versus combination chemotherapy with capecitabine, irinotecan and oxaliplatin in advanced colorectal cancer, an interim safety analysis. A Dutch Colorectal Cancer Group (DCCG) phase III study. Ann. Oncol..

[111] Jensen S.A., Sorensen J.B. (2006). Risk factors and prevention of cardiotoxicity induced by 5-fluorouracil or capecitabine. Cancer Chemoth. Pharm..

[112] Velenik V., Anderluh F., Oblak I., Strojan P., Zakotnik B. (2006). Capecitabine as a radiosensitizing agent in neoadjuvant treatment of locally advanced resectable rectal cancer: Prospective phase II trial. Croat Med. J..

[113] Rapidis A.D., Trichas M., Stavrinidis E., Roupakia A., Ioannidou G., Kritselis G., Liossi P., Giannakouras G., Douzinas E.E., Katsilieris I. (2006). Induction chemotherapy followed by concurrent chemoradiation in advanced squamous cell carcinoma of the head and neck: Final results from a phase II study with docetaxel, cisplatin and 5-fluorouracil with a four-year follow-up. Oral Oncol..

[114] Yerushalmi R., Idelevich E., Dror Y., Stemmer S.M., Figer A., Sulkes A., Brenner B., Loven D., Dreznik Z., Nudelman I. (2006). Preoperative chemoradiation in rectal cancer: Retrospective comparison between capecitabine and continuous infusion of 5-fluorouracil. J. Surg. Oncol..

[115] Tsibiribi P., Descotes J., Lombard-Bohas C., Barel C., Bui-Xuan B., Belkhiria M., Tabib A., Timour Q. (2006). Cardiotoxicity of 5-fluorouracil in 1350 patients with no prior history of heart disease. Bull Cancer.

[116] Klautke G., Küchenmeister U., Foitzik T., Ludwig K., Prall F., Klar E., Fietkau R. (2006). Concurrent chemoradiation with capecitabine and weekly irinotecan as preoperative treatment for rectal cancer: Results from a phase I/II study. Brit J. Cancer.

[117] Giordano K.F., Jatoi A., Stella P.J., Foster N., Tschetter L.K., Alberts S.R., Dakhil S.R., Mailliard J.A., Flynn P.J., Nikcevich D.A. (2006). Docetaxel and capecitabine in patients with metastatic adenocarcinoma of the stomach and gastroesophageal junction: A phase II study from the North Central Cancer Treatment Group. Ann. Oncol..

[118] Giantonio B.J., Levy D.E., O’dwyer P.J., Meropol N.J., Catalano P.J., Benson A.B., Eastern Cooperative Oncology Group (2006). A phase II study of high-dose bevacizumab in combination with irinotecan, 5-fluorouracil, leucovorin, as initial therapy for advanced colorectal cancer: Results from the eastern cooperative oncology group study E2200. Ann. Oncol..

[119] Jatoi A., Murphy B.R., Foster N.R., Nikcevich D.A., Alberts S.R., Knost J.A., Fitch T.R., Rowland K.M., North Central Cancer Treatment Group (2006). Oxaliplatin and capecitabine in patients with metastatic adenocarcinoma of the esophagus, gastroesophageal junction and gastric cardia: A phase II study from the North Central Cancer Treatment Group. Ann. Oncol..

[120] Baghi M., Hambek M., Wagenblast J., May A., Gstoettner W., Knecht R. (2006). A phase II trial of docetaxel, cisplatin and 5-fluorouracil in patients with recurrent squamous cell carcinoma of the head and neck (SCCHN). Anticancer Res..

[121] Berlin J.D., Feng Y., Catalano P., Abbruzzese J.L., Philip P.A., McWilliams R.R., Lowy A.M., Benson A.B., Blackstock A.W. (2018). An Intergroup Randomized Phase II Study of Bevacizumab or Cetuximab in Combination with Gemcitabine and in Combination with Chemoradiation in Patients with Resected Pancreatic Carcinoma: A Trial of the ECOG-ACRIN Cancer Research Group (E2204). Oncology.

[122] Landry J.C., Feng Y., Prabhu R.S., Cohen S.J., Staley C.A., Whittington R., Sigurdson E.R., Nimeiri H., Verma U., Benson A.B. (2015). Phase II Trial of Preoperative Radiation With Concurrent Capecitabine, Oxaliplatin, and Bevacizumab Followed by Surgery and Postoperative 5-Fluorouracil, Leucovorin, Oxaliplatin (FOLFOX), and Bevacizumab in Patients With Locally Advanced Rectal Cancer: 5-Year Clinical Outcomes ECOG-ACRIN Cancer Research Group E3204. Oncologist.

[123] Gianni L., Baselga J., Eiermann W., Guillem Porta V., Semiglazov V., Lluch A., Zambetti M., Sabadell D., Raab G., Llombart Cussac A. (2005). Feasibility and tolerability of sequential doxorubicin/paclitaxel followed by cyclophosphamide, methotrexate, and fluorouracil and its effects on tumor response as preoperative therapy. Clin. Cancer Res..

[124] Meydan N., Kundak I., Yavuzsen T., Oztop I., Barutca S., Yilmaz U., Alakavuklar M.N. (2005). Cardiotoxicity of de Gramont’s Regimen: Incidence, Clinical Characteristics and Long-term Follow-up. Jpn. J. Clin. Oncol..

[125] Meropol N.J., Feng Y., Grem J.L., Mulcahy M.F., Catalano P.J., Kauh J.S., Hall M.J., Saltzman J.N., George T.J., Zangmeister J. (2018). Phase 2 study of treatment selection based on tumor thymidylate synthase expression in previously untreated patients with metastatic colorectal cancer: A trial of the ECOG-ACRIN Cancer Research Group (E4203). Cancer-Am. Cancer Soc..

[126] Tsavaris N., Kosmas C., Vadiaka M., Skopelitis E., Kopteridis P., Pamouki S., Efremidis M., Kasparian H., Moisakis I., Sakelariou D. (2005). 5-fluorouracil cardiotoxicity is a rare, dose and schedule-dependent adverse event: A prospective study. J. BUON.

[127] Ceyhan C., Meydan N., Barutca S., Tekten T., Onbasili A.O., Ozturk B., Unal S. (2005). Ultrasound Tissue Characterization by Integrated Backscatter for Analyzing Fluorouracil Induced Myocardial Damage. Echocardiography.

[128] Bontenbal M., Creemers G.J., Braun H.J., de Boer A.C., Janssen J.T., Leys R.B., Ruit J.B., Goey S.H., van der Velden P.C., Kerkhofs L.G. (2005). Phase II to III study comparing doxorubicin and docetaxel with fluorouracil, doxorubicin, and cyclophosphamide as first-line chemotherapy in patients with metastatic breast cancer: Results of a dutch community setting trial for the clinical trial group of the comprehensive cancer centre. J. Clin. Oncol..

[129] Levine M.N., Pritchard K.I., Bramwell V.H., Shepherd L.E., Tu D., Paul N., National Cancer Institute of Canada Clinical Trials Group (2005). Randomized trial comparing cyclophosphamide, epirubicin, and fluorouracil with cyclophosphamide, methotrexate, and fluorouracil in premenopausal women with node-positive breast cancer: Update of National Cancer Institute of Canada Clinical Trials Group Trial MA5. J. Clin. Oncol..

[130] Lordick F., Lorenzen S., Stollfuss J., Vehling-Kaiser U., Kullmann F., Hentrich M., Zumschlinge R., Dietzfelbinger H., Thoedtmann J., Hennig M. (2005). Phase II study of weekly oxaliplatin plus infusional fluorouracil and folinic acid (FUFOX regimen) as first-line treatment in metastatic gastric cancer. Brit J. Cancer.

[131] Ng M., Cunningham D., Norman A.R. (2005). The frequency and pattern of cardiotoxicity observed with capecitabine used in conjunction with oxaliplatin in patients treated for advanced colorectal cancer (CRC). Eur. J. Cancer.

[132] Smaradottir A., Siddiqi A., Ray C., Haider J., Azrin M., Hegde U. (2005). Increased incidence of cardiotoxicity after infusional 5-fluorouracil, cisplatin and docetaxel combination chemotherapy in patients with locally advanced head and neck cancer. J. Clin. Oncol..

[133] Keene K.S., Rich T.A., Penberthy D.R., Shepard R.C., Adams R., Jones R.S. (2005). Clinical experience with chronomodulated infusional 5-fluorouracil chemoradiotherapy for pancreatic adenocarcinoma. Int. J. Radiat. Oncol..

[134] Feliu J., Escudero P., Llosa F., Bolaños M., Vicent J.M., Yubero A., Sanz-Lacalle J.J., Lopez R., Lopez-Gómez L., Casado E. (2005). Capecitabine as first-line treatment for patients older than 70 years with metastatic colorectal cancer: An Oncopaz Cooperative Group Study. J. Clin. Oncol..

[135] Berruti A., Bitossi R., Gorzegno G., Bottini A., Generali D., Milani M., Katsaros D., Rigault de la Longrais I.A., Bellino R., Donadio M. (2005). Paclitaxel, vinorelbine and 5-fluorouracil in breast cancer patients pretreated with adjuvant anthracyclines. Brit J. Cancer.

[136] Erman M., Baltali E., Karaoglu A., Abali H., Engin H., Ozisik Y., Guler N., Altundag K., Tekuzman G., Atahan I.L. (2005). A phase II study on the safety and efficacy of 5-fluorouracil, epirubicin, cyclophosphamide (FEC) followed by paclitaxel in the adjuvant treatment of breast cancer. Cancer Investig..

[137] Berruti A., Bitossi R., Bottini A., Bonardi S., Donadio M., Nigro C., Bertetto O., Danese S., Bertone E., Sarobba M.G. (2005). Combination regimen of epirubicin, vinorelbine and 5-fluorouracil continuous infusion as first-line chemotherapy in anthracycline-naive metastatic breast cancer patients. Eur. J. Cancer.

[138] Li T., Guo M., Gradishar W.J., Sparano J.A., Perez E.A., Wang M., Sledge G.W. (2012). A phase II trial of capecitabine in combination with the farnesyltransferase inhibitor tipifarnib in patients with anthracycline-treated and taxane-resistant metastatic breast cancer: An Eastern Cooperative Oncology Group Study (E1103). Breast Cancer Res Tr.

[139] Oztop I., Gencer M., Okan T., Yaren A., Altekin E., Turker S., Yilmaz U. (2004). Evaluation of Cardiotoxicity of a Combined Bolus plus Infusional 5-Fluorouracil/Folinic Acid Treatment by Echocardiography, Plasma Troponin I Level, QT Interval and Dispersion in Patients with Gastrointestinal System Cancers. Jpn J. Clin. Oncol..

[140] Martin M., Villar A., Sole-Calvo A., Gonzalez R., Massuti B., Lizon J., Camps C., Carrato A., Casado A., Candel M.T. (2003). Doxorubicin in combination with fluorouracil and cyclophosphamide (i.v. FAC regimen, day 1, 21) versus methotrexate in combination with fluorouracil and cyclophosphamide (i.v. CMF regimen, day 1, 21) as adjuvant chemotherapy for operable breast cancer: A study by the GEICAM group. Ann. Oncol..

[141] Daniele B., Rosati G., Tambaro R., Ottaiano A., De Maio E., Pignata S., Iaffaioli R.V., Rossi A., Manzione L., Gallo C. (2003). First-line chemotherapy with fluorouracil and folinic acid for advanced colorectal cancer in elderly patients - A phase II study. J. Clin. Gastroenterol..

[142] Elomaa I., Joensuu H., Blomqvist C. (2003). Vinorelbine, epirubicin and fluorouracil as first-line therapy in metastatic breast cancer—A phase II trial. Acta Oncol..

[143] Wacker A., Lersch C., Scherpinski U., Reindl L., Seyfarth M. (2003). High incidence of Angina pectoris in patients treated with 5-fluorouracil-A planned surveillance study with 102 patients. Oncology.

[144] Comparison of Adjuvant Chemotherapy Regimens in Treating Stage II/III Rectal Cancer. https://clinicaltrials.gov/ct2/show/NCT00068692?term=E3201&draw=2&rank=1.

[145] Hitt R., Paz-Ares L., Brandáriz A., Castellano D., Peña C., Millán J.M., Calvo F., Ortiz de Urbina D., López E., Alvarez-Vicent J.J. (2002). Induction chemotherapy with paclitaxel, cisplatin and 5-fluorouracil for squamous cell carcinoma of the head and neck: Long-term results of a phase II trial. Ann. Oncol..

[146] Vaishampayan U.N., Ben-Josef E., Philip P.A., Vaitkevicius V.K., Du W., Levin K.J., Shields A.F. (2002). A single-institution experience with concurrent capecitabine and radiation therapy in gastrointestinal malignancies. Int. J. Radiat. Oncol..

[147] Tsavaris N., Kosmas C., Vadiaka M., Efremidis M., Zinelis A., Beldecos D., Sakelariou D., Koufos C., Stamatelos G. (2002). Cardiotoxicity following different doses and schedules of 5-fluorouracil administration for malignancy-a survey of 427 patients. Med. Sci. Monit..

[148] Giantonio B.J., Catalano P.J., Meropol N.J., O’Dwyer P.J., Mitchell E.P., Alberts S.R., Schwartz M.A., Benson A.B., Eastern Cooperative Oncology Group Study E3200 (2007). Bevacizumab in Combination with Oxaliplatin, Fluorouracil, and Leucovorin (FOLFOX4) for Previously Treated Metastatic Colorectal Cancer: Results From the Eastern Cooperative Oncology Group Study E3200. J. Clin. Oncol..

[149] Hartung G., Hofheinz R.D., Wein A., Riedel C., Rost A., Fritze D., Kreuser E.D., Drees M., Kühnel J., Hehlmann R. (2001). Phase II study of a weekly 24-hour infusion with 5-fluorouracil and simultaneous sodium-folinic acid in the first-line treatment of metastatic colorectal cancer. Onkologie.

[150] Dencausse Y., Sturm J., Hartung G., Dietzler P., Edler L., Bambach M., Wojatschek C., Lindemann H., Qeisser W. (2001). Adjuvant radio-chemotherapy in stage II-III rectal cancer with 24-hour infusion of high-dose 5-fluorouracil and folinic acid: Evaluation of feasibility. Onkologie.

[151] Recchia F., De Filippis S., Rosselli M., Saggio G., Pompili P., Piccinini M., Rea S. (2001). Combined 5-fluorouracil infusion with fractionated epirubicin and cyclophosphamide in advanced breast cancer. Am J. Clin. Oncol.-Canc.

[152] Riccardi A., Pugliese P., Danova M., Brugnatelli S., Grasso D., Giordano M., Bernardo G., Giardina G., Fava S., Montanari G. (2001). A phase II study of sequential 5-fluorouracil, epirubicin and cyclophosphamide (FEC) and paclitaxel in advanced breast cancer (Protocol PVBC 97/01). Brit J. Cancer.

[153] Piccart M.J., Di Leo A., Beauduin M., Vindevoghel A., Michel J., Focan C., Tagnon A., Ries F., Gobert P., Finet C. (2001). Phase III trial comparing two dose levels of epirubicin combined with cyclophosphamide with cyclophosphamide, methotrexate, and fluorouracil in node-positive breast cancer. J. Clin. Oncol..

[154] Jassem J., Pieńkowski T., Płuzańska A., Jelic S., Gorbunova V., Mrsic-Krmpotic Z., Berzins J., Nagykalnai T., Wigler N., Renard J. (2001). Central & Eastern Europe and Israel Pacitaxel Breast Cancer Study Group: Doxorubicin and paclitaxel versus fluorouracil, doxorubicin, and cyclophosphamide as first-line therapy for women with metastatic breast cancer. Final results of a randomized phase III multicenter trial. J. Clin. Oncol..

[155] Peiffert D., Giovannini M., Ducreux M., Michel P., François E., Lemanski C., Mirabel X., Cvitkovic F., Luporsi E., Conroy T. (2001). Digestive Tumours Group of the French ’Fédération Nationale des Centres de Lutte Contre le Cancer’. High-dose radiation therapy and neoadjuvant plus concomitant chemotherapy with 5-fluorouracil and cisplatin in patients with locally advanced squamous-cell anal canal cancer: Final results of a phase II study. Ann. Oncol..

[156] Ackland S.P., Anton A., Breitbach G.P., Colajori E., Tursi J.M., Delfino C., Efremidis A., Ezzat A., Fittipaldo A., Kolaric K. (2001). HEPI 013 study group. Dose-intensive epirubicin-based chemotherapy is superior to an intensive intravenous cyclophosphamide, methotrexate, and fluorouracil regimen in metastatic breast cancer: A randomized multinational study. J. Clin. Oncol..

[157] Pienkowski T., Jagiello-Gruszfeld A. (2001). Five-day infusion of fluorouracil and vinorelbine for advanced breast cancer patients treated previously with anthracyclines. Int. J. Clin. Pharmacol. Res..

[158] Blum J.L., Dieras V., Lo Russo P.M., Horton J., Rutman O., Buzdar A., Osterwalder B. (2001). Multicenter, Phase II Study of Capecitabine in Taxane-Pretreated Metastatic Breast Carcinoma Patients. Cancer.

[159] Hoff P.M., Ansari R., Batist G., Cox J., Kocha W., Kuperminc M., Maroun J., Walde D., Weaver C., Harrison E. (2001). Comparison of Oral Capecitabine Versus Intravenous Fluorouracil Plus Leucovorin as First-Line Treatment in 605 Patients with Metastatic Colorectal Cancer: Results of a Randomized Phase III Study. J. Clin. Oncol..

[160] Lin J.K., Wang W.S., Hsieh R.K., Hsu T.C., Chiou T.J., Liu J.H., Fan F.S., Yen C.C., Lin T.C., Jiang J.K. (2000). Phase II study of oral tegafur-uracil and folinic acid as first-line therapy for metastatic colorectal cancer: Taiwan experience. Jpn. J. Clin. Oncol..

[161] Zambelli A., Robustelli della Cuna F.S., Ponchio L., Ucci G., Da Prada G.A., Robustelli della Cuna G. (2000). Four-day infusion of fluorouracil plus vinorelbine as salvage treatment of heavily pretreated metastatic breast cancer. Breast Cancer Res. Tr..

[162] Balloni L., Porta C., Rossi S., Gola A., Pugliese P., Ferrari S., Bovio A., Danova M., Riccardi A. (2000). Left ventricular function in colon cancer patients receiving adjuvant fluoro-folate chemotherapy: An echocardiographic study. Oncol. Rep..

[163] Susnjar S., Vasović S., Nesković-Konstantinović Z., Stamatović L., Lukić V., Colaković S., Mitrovic L., Jelić S., Radulović S. (1999). Mitoxantrone, 5-fluorouracil and low-dose leucovorin in doxorubicin-resistant advanced breast cancer patients: A phase II study. Tumori.

[164] Valero V., Buzdar A.U., Theriault R.L., Azarnia N., Fonseca G.A., Willey J., Ewer M., Walters R.S., Mackay B., Podoloff D. (1999). Phase II trial of liposome-encapsulated doxorubicin, cyclophosphamide, and fluorouracil as first-line therapy in patients with metastatic breast cancer. J. Clin. Oncol..

[165] Hasbini A., Mahjoubi R., Fandi A., Chouaki N., Taamma A., Lianes P., Cortès-Funes H., Alonso S., Armand J.P., Cvitkovic E. (1999). Phase II trial combining mitomycin with 5-fluorouracil, epirubicin, and cisplatin in recurrent and metastatic undifferentiated carcinoma of nasopharyngeal type. Ann. Oncol..

[166] Blum J.L., Jones S.E., Buzdar A.U., LoRusso P.M., Kuter I., Vogel C., Osterwalder B., Burger H.U., Brown C.S., Griffin T. (1999). Multicenter Phase II Study of Capecitabine in Paclitaxel-Refractory Metastatic Breast Cancer. J. Clin. Oncol..

[167] Blajman C., Balbiani L., Block J., Coppola F., Chacon R., Fein L., Bonicatto S., Alvarez A., Schmilovich A., Delgado F.M. (1999). A prospective, randomized phase III trial comparing combination chemotherapy with cyclophosphamide, doxorubicin, and 5-fluorouracil with vinorelbine plus doxorubicin in the treatment of advanced breast carcinoma. Cancer-Am. Cancer Soc..

[168] Birkenhake S., Leykamm S., Martus P., Sauer R. (1999). Concomitant radiochemotherapy with 5-FU and cisplatin for invasive bladder cancer - Acute toxicity and first results. Strahlenther. Onkol..

[169] Milano G., Etienne M.C., Pierrefite V., Barberi-Heyob M., Deporte-Fety R., Renée N. (1999). Dihydropyrimidine dehydrogenase deficiency and fluorouracil-related toxicity. Brit J. Cancer.

[170] Warner E., Jensen J.L., Cripps C., Khoo K.E., Goel R., Kerr I.A., Bjarnason G.A., Fields A.L., Hrincu A. (1999). Outpatient 5-fluorouracil, folinic acid and cisplatin in patients with advanced esophageal carcinoma. Acta Oncol..

[171] Andersson M., Madsen E.L., Overgaard M., Rose C., Dombernowsky P., Mouridsen H.T. (1999). Doxorubicin versus methotrexate both combined with cyclophosphamide, 5-fluorouracil and tamoxifen in postmenopausal patients with advanced breast cancer - a randomised study with more than 10 years follow-up from the Danish Breast Cancer Cooperative Group. Eur. J. Cancer.

[172] Mantovani G., Gebbia V., Airoldi M., Bumma C., Contu P., Bianchi A., Ghiani M., Dessì D., Massa E., Curreli L. (1998). Neo-adjuvant chemo-(immuno-)therapy of advanced squamous-cell head and neck carcinoma: A multicenter, phase III, randomized study comparing cisplatin plus 5-fluorouracil with cisplatin plus 5-FU plus recombinant interleukin 2. Cancer Immunol. Immun..

[173] Papadimitriou C.A., Dimopoulos M.A., Ampela C., Louvrou-Fertaki A., Anagnostopoulos A., Athanassiades P., Stamatelopoulos S., Keramopoulos A. (1998). Sequential administration of doxorubicin and paclitaxel followed by cyclophosphamide, methotrexate and 5-fluorouracil combination (CMF) in women with metastatic breast cancer. Oncology.

[174] Katona C., Kralovánszky J., Rosta A., Pandi E., Fónyad G., Tóth K., Jeney A. (1998). Putative role of dihydropyrimidine dehydrogenase in the toxic side effect of 5-fluorouracil in colorectal cancer patients. Oncology.

[175] Levine M.N., Bramwell V.H., Pritchard K.I., Norris B.D., Shepherd L.E., Abu-Zahra H., Findlay B., Warr D., Bowman D., Myles J. (1998). Randomized trial of intensive cyclophosphamide, epirubicin, and fluorouracil chemotherapy compared with cyclophosphamide, methotrexate, and fluorouracil in premenopausal women with node-positive breast cancer. J. Clin. Oncol..

[176] Fetting J.H., Gray R., Fairclough D.L., Smith T.J., Margolin K.A., Citron M.L., Grove-Conrad M., Cella D., Pandya K., Robert N. (1998). Sixteen-week multidrug regimen versus cyclophosphamide, doxorubicin, and fluorouracil as adjuvant therapy for node-positive, receptor-negative breast cancer: An intergroup study. J. Clin. Oncol..

[177] Seitz J.F., Perrier H., Giovannini M., Capodano G., Bernardini D., Bardou V. (1998). 5-Fluorouracil, high-dose folinic acid and mitomycin C combination chemotherapy in previously treated patients with advanced colorectal carcinoma. J. Chemother..

[178] Tominaga T., Nomura Y., Uchino J., Hirata K., Kimura M., Yoshida M., Aoyama H., Kinoshita H., Koyama H., Monden Y. (1998). Cyclophosphamide, adriamycin, 5-fluorouracil and high-dose toremifene for patients with advanced/recurrent breast cancer. Jpn J. Clin. Oncol..

[179] Wang W.S., Chen P.M., Chiou T.J., Liu J.H., Lin J.K., Lin T.C., Chen W.S., Jiang J.K., Yen C.C., Fan F.S. (1998). Weekly 24-hour infusion of high-dose 5-fluorouracil and leucovorin in patients with advanced colorectal cancer: Taiwan experience. Jpn J. Clin. Oncol..

[180] Taylor S.G., Murthy A.K., Griem K.L., Recine D.C., Kiel K., Blendowski C., Hurst P.B., Showel J.T., Hutchinson J.C., Campanella R.S. (1997). Concomitant cisplatin/5-FU infusion and radiotherapy in advanced head and neck cancer: 8-year analysis of results. Head Neck-J. Sci. Spec..

[181] Yen C.C., Tung S.L., Hsieh R.K., Chiou T.J., Liu J.H., Wang W.S., Chen P.M. (1997). Treatment of previously treated metastatic breast cancer by mitoxantrone and 48-hour continuous infusion of high-dose 5-FU and leucovorin (MFL): Low palliative benefit and high treatment-related toxicity. Jpn J. Clin. Oncol..

[182] Meyer C.C., Calis K.A., Burke L.B., Walawander C.A., Grasela T.H. (1997). Symptomatic cardiotoxicity associated with 5-fluorouracil. Pharmacotherapy.

[183] Bascioni R., Giorgi F., Silva R.R., Acito L., Giustini L., De Signoribus G., Giuliodori L., Testa E. (1997). Mitoxantrone, fluorouracil, and L-folinic acid in anthracycline-pretreated metastatic breast cancer patients. Breast Cancer Res. Tr..

[184] Kroning K.C., Pernice L.M., Pantalone D., Neri B. Long-term epidoxorubicin and high dose leucovorin plus 5-fluorouracil therapy in advanced gastric carcinoma. Proceedings of the 2nd International Gastric Cancer Congress.

[185] Doci R., Zucali R., La Monica G., Meroni E., Kenda R., Eboli M., Lozza L. (1996). Primary chemoradiation therapy with fluorouracil and cisplatin for cancer of the anus: Results in 35 consecutive patients. J. Clin. Oncol..

[186] Ychou M., Astre C., Rouanet P., Fabre J.M., Saint-Aubert B., Domergue J., Ribard D., Ciurana A.J., Janbon C., Pujol H. (1996). A phase II study of 5-fluorouracil, leucovorin and cisplatin (FLP) for metastatic gastric cancer. Eur. J. Cancer.

[187] Kok T.C., van der Gaast A., Splinter T.A. (1996). 5-Fluorouracil and folinic acid in advanced adenocarcinoma of the esophagus or esophago-gastric junction area. Ann. Oncol..

[188] Gebbia V., Testa A., Majello E., Cannata G., Tirrito M.L., Mastrandrea G., Feo M., Bajardi G., Colucci G., Gebbia N. (1996). Subcutaneous low-dose interleukin-2 and intravenous 5-fluorouracil plus high-dose levofolinic acid as salvage treatment for metastatic colorectal carcinoma. Anti-Cancer Drug.

[189] Hainsworth J.D., Jones S.E., Mennel R.G., Blum J.L., Greco F.A. (1996). Paclitaxel with mitoxantrone, fluorouracil, and high-dose lencovorin in the treatment of metastatic breast cancer: A phase II trial. J. Clin. Oncol..

[190] Hartung G., Queisser W., Diezler P., Hagmüller E., Edler L., Jacob I., Wojatschek C., Seifert A., Weiss H., Weh H.-J. (1996). Adjuvant chemotherapy with 5-fluorouracil and folinic acid in colorectal cancer: Evaluation of toxicity. Onkologie.

[191] Colozza M., Gori S., Mosconi A.M., Anastasi P., Basurto C., Ludovini V., De Angelis V., Giansanti M., Tonato M. (1996). Salvage chemotherapy in metastatic breast cancer: An experience with the combination of mitoxantrone, 5-fluorouracil, and L-leucovorin. Breast Cancer Res. Tr..

[192] Mammoliti S., Merlini L., Caroti C., Gallo L. (1996). Phase II study of mitoxantrone, 5-fluorouracil, and levo-leucovorin (MLF) in elderly advanced breast cancer patients. Breast Cancer Res. Tr..

[193] Bécouarn Y.H., Brunet R.C., Rouhier M.L., Bussières E.J., Avril A.R., Richaud P.M., Dilhuydy J.M. (1995). High dose folinic acid and 5-fluorouracil bolus and continuous infusion for patients with advanced colorectal cancer. Cancer.

[194] Blijham G.H. (1996). The EORTC GI group experience with high-dose infusional 5-FU in colorectal cancer. J. Infus Chemother..

[195] Macdonald J.S., Fleming T.R., Peterson R.F., Berenberg J.L., McClure S., Chapman R.A., Eyre H.J., Solanki D., Cruz A.B., Gagliano R. (1995). Adjuvant chemotherapy with 5-FU, adriamycin, and mitomycin-C (FAM) versus surgery alone for patients with locally advanced gastric adenocarcinoma: A Southwest Oncology Group study. Ann. Surg. Oncol..

[196] Weidmann B., Jansen W., Heider A., Niederle N. (1995). 5-Fluorouracil cardiotoxicity with left ventricular dysfunction under different dosing regimens. Am. J. Cardiol..

[197] Kolaric K., Bradamante V., Cervek J., Cieslinska A., Cisarz-Filipcak E., Denisov L.E., Donat D., Drosik K., Gershanovic M., Hudziec P. (1995). A phase II trial of cardioprotection with Cardioxane (ICRF-187) in patients with advanced breast cancer receiving 5-fluorouracil, doxorubicin and cyclophosphamide. Oncology.

[198] Alonso M.C., Tabernero J.M., Ojeda B., Llanos M., Solà C., Climent M.A., Seguí M.A., López J.J. (1995). A phase III randomized trial of cyclophosphamide, mitoxantrone, and 5-fluorouracil (CNF) versus cyclophosphamide, adriamycin, and 5-fluorouracil (CAF) in patients with metastatic breast cancer. Breast Cancer Res. Tr..

[199] Leonardi V., Meli M., Palmeri S., Russo A., Rini G.B., Peralta S., Rausa L. (1995). A phase II trial of mitoxantrone plus cyclophosphamide and 5-fluorouracil in modulation with levo-folinate for advanced breast cancer patients. J. Chemother..

[200] Carmo-Pereira J., Henriques E., Costa F.O. (1995). 5-fluorouracil, epirubicin and cyclophosphamide, as first-line cytotoxic chemotherapy for disseminated breast-carcinoma - a phase-ii study. Breast.

[201] Haas N.B., Schilder R.J., Nash S., Weiner L.M., Catalano R.C., Ozols R.F., O’Dwyer P.J. (1995). A Phase II trial of weekly infusional 5-fluorouracil in combination with low-dose leucovorin in patients with advanced colorectal cancer. Investig. New Drug.

[202] Klastersky J., Sculier J.P., Ries F., Dabouis G., Libert P., Schmerber J., Thiriaux J., Berchier M.C., Bureau G., Van Cutsem O. (1994). A four-drug combination chemotherapy with cisplatin, mitomycin, vindesine and 5-fluorouracil. A regimen associated with major toxicity in patients with advanced non-small lung cancer. Ann. Oncol..

[203] Morere J.F., Duran A., Tcherakian F., Boaziz C., Valeyre D., Battesti J.P., Breu J.L., Israel L. (1994). Cisplatin-5-fluorouracil in small cell lung cancer. A phase II study in 109 patients. Lung Cancer..

[204] Villar-Grimalt A., Aranda E., Massutí B., Belón J., Antón A., Jimeno J.M., Candel M.T., García de Paredes M.L., Colajori E. (1994). Phase II Study with Iododoxorubicin in Measurable Advanced Colorectal Adenocarcinoma. Effective Rescue Using Weekly High-Dose 5-Fluorouracil (WFU). Tumori.

[205] Haarstad H., Jacobsen A.B., Schjølseth S.A., Risberg T., Fossa S.D. (1994). Interferon-alpha, 5-FU and prednisone in metastatic renal cell carcinoma: A phase II study. Ann. Oncol..

[206] Akhtar S.S., Salim K.P., Bano Z.A. (1993). Symptomatic cardiotoxicity with high-dose 5-fluorouracil infusion: A prospective study. Oncology.

[207] Langer C.J., Curran W.J., Keller S.M., Catalano R., Fowler W., Blankstein K., Litwin S., Bagchi P., Nash S., Comis R. (1993). Report of phase II trial of concurrent chemoradiotherapy with radical thoracic irradiation (60 Gy), infusional fluorouracil, bolus cisplatin and etoposide for clinical stage IIIB and bulky IIIA non-small cell lung cancer. Int. J. Radiat Oncol..

[208] Depondt J., Gehanno P., Martin M., Lelievre G., Guerrier B., Peytral C., Schott H., Pellae-Cosset B. (1993). Neoadjuvant chemotherapy with carboplatin/5-fluorouracil in head and neck cancer. Oncology.

[209] Carmopereira J., Costa F.O., Henriques E. (1993). Mitoxantrone, folinic acid, 5-fluorouracil and prednisone as first-line chemotherapy for advanced breast carcinoma. A phase II study. Eur. J. Cancer.

[210] Neri B., Gemelli M.T., Pantalone D., Andreoli F., Bruno S., Fabbroni S., Leone V., Valeri A., Borrelli D. (1993). Epidoxorubicin and high dose leucovorin plus 5-fluorouracil in advanced gastric cancer: A phase II study. Anti-Cancer Drug.

[211] Jassem J., Gyergyay F., Kerpel-Fronius S., Nagykálnai T., Baumöhl J., Verweij J., Vuletic L., Mechl Z., Drozd-Lula M., Jelic S. (1993). Combination of daily 4-h infusion of 5-fluorouracil and cisplatin in the treatment of advanced head and neck squamous-cell carcinoma: A South-East European Oncology Group study. Cancer Chemoth. Pharm..

[212] Keefe D.L., Roistacher N., Pierri M.K. (1993). Clinical cardiotoxicity of 5-fluorouracil. J. Clin. Pharmacol..

[213] Schöber C., Papageorgiou E., Harstrick A., Bokemeyer C., Mügge A., Stahl M., Wilke H., Poliwoda H., Hiddemann W., Köhne-Wömpner C.H. (1993). Cardiotoxicity of 5-fluorouracil in combination with folinic acid in patients with gastrointestinal cancer. Cancer.

[214] Levine M.N., Bramwell V., Pritchard K., Perrault D., Findlay B., Abu-Zahra H., Warr D., Arnold A., Skillings J. (1993). A pilot study of intensive cyclophosphamide, epirubicin and fluorouracil in patients with axillary node positive or locally advanced breast cancer. Eur. J. Cancer.

[215] Kuzel T.M., Tallman M.S., Shevrin D., Braud E., Kilton L., Johnson P., Kozlowski J., Vogelzang N.J., Blough R., Benson A.B. (1993). A phase II study of continuous infusion 5-fluorouracil in advanced hormone refractory prostate cancer. An Illinois Cancer Center Study, Y. Cancer.

[216] Citron M.L., Modeas C., Propert K., Goutsou M., Green M.R. (1992). Phase II trial of high-dose 24-hour continuous intravenous 5-fluorouracil for advanced non-small cell lung cancer: A Cancer and Leukemia Group B study. Cancer Investig..

[217] de Forni M., Malet-Martino M.C., Jaillais P., Shubinski R.E., Bachaud J.M., Lemaire L., Canal P., Chevreau C., Carrié D., Soulié P. (1992). Cardiotoxicity of high-dose continuous infusion fluorouracil: A prospective clinical study. J. Clin. Oncol..

[218] Zaniboni A., Simoncini E., Marpicati P., Meriggi F., Arcangeli G., Garattini P., Raffaglio E., Ferragni A., Marini G. (1991). Mitomycin-C, adriamycin, 5-fluorouracil and leucovorin (L-FAM2) in the treatment of advanced gastric cancer: A phase II study. Tumori.

[219] Gradishar W., Vokes E., Schilsky R., Weichselbaum R., Panje W. (1991). Vascular events in patients receiving high-dose infusional 5-fluorouracil-based chemotherapy: The University of Chicago experience. Med. Pediatr. Oncol..

[220] Zamagni C., Martoni A., Ercolino L., Baroni M., Tanneberger S., Pannuti F. (1991). 5-Fluorouracil, epirubicin and cyclophosphamide (FEC combination) in advanced breast cancer. J. Chemother..

[221] Periti P., Pannuti F., Della Cuna G.R., Mazzei T., Mini E., Martoni A., Preti P., Ercolino L., Pavesi L., Ribecco A. (1991). Combination chemotherapy with cyclophosphamide, fluorouracil, and either epirubicin or mitoxantrone: A comparative randomized multicenter study in metastatic breast carcinoma. Cancer Investig..

[222] Schlumberger M., Brugieres L., Gicquel C., Travagli J.P., Droz J.P., Parmentier C. (1991). 5-Fluorouracil, doxorubicin, and cisplatin as treatment for adrenal cortical carcinoma. Cancer.

[223] Samonigg H., Stöger H., Bauernhofer T., Schmid M., Kasparek A.K., Kuss I., Kaul M., Heberle U., Vieder L., Dusleag J. (1990). Combination therapy of 4’-O-tetrahydropyranyl-doxorubicin, 5-fluorouracil, and high-dose folinic acid in patients with advanced breast cancer: A phase I-II study (preliminary results). Am J. Clin. Oncol..

[224] Jeremic B., Jevremovic S., Djuric L., Mijatovic L. (1990). Cardiotoxicity during chemotherapy treatment with 5-fluorouracil and cisplatin. J. Chemother..

[225] Eskilsson J., Albertsson M. (1990). Failure of preventing 5-fluorouracil cardiotoxicity by prophylactic treatment with verapamil. Acta Oncol..

[226] Havsteen H., Brynjolf I., Svahn T., Dombernowsky P., Godtfredsen J., Munck O. (1989). Prospective evaluation of chronic cardiotoxicity due to high-dose epirubicin or combination chemotherapy with cyclophosphamide, methotrexate, and 5-fluorouracil. Cancer Chemother Pharmacol..

[227] Rezkalla S., Kloner R.A., Ensley J., al-Sarraf M., Revels S., Olivenstein A., Bhasin S., Kerpel-Fronious S., Turi Z.G. (1989). Continuous ambulatory ECG monitoring during fluorouracil therapy: A prospective study: Continuous ambulatory ECG monitoring during fluorouracil therapy: A prospective study. J. Clin. Oncol..

[228] Eskilsson J., Albertsson M., Mercke C. (1988). Adverse cardiac effects during induction chemotherapy treatment with cis-platin and Mluorouracil. Radiother. Oncol..

[229] Labianca R., Beretta G., Clerici M., Fraschini P., Luporini G. (1982). Cardiac toxicity of 5-fluorouracil: A study on 1083 patients. Tumori.

[230] Pottage A., Holt S. (1978). Ludgate S Fluorouracil cardiotoxicity. Br. Med. J..

[231] Kanduri J., More L.A., Godishala A., Asnani A. (2019). Fluoropyrimidine-Associated Cardiotoxicity. Cardiol. Clin..

[232] Lotrionte M., Biondi-Zoccai G., Abbate A., Lanzetta G., D’Ascenzo F., Malavasi V., Peruzzi M., Frati G., Palazzoni G. (2013). Review and meta-analysis of incidence and clinical predictors of anthracycline cardiotoxicity. Am. J. Cardiol..

[233] Lyon A.R., López-Fernández T., Couch L.S., Asteggiano R., Aznar M.C., Bergler-Klein J., Boriani G., Cardinale D., Cordoba R., Cosyns B. (2022). **2022** ESC Guidelines on cardio-oncology developed in collaboration with the European Hematology Association (EHA), the European Society for Therapeutic Radiology and Oncology (ESTRO) and the International Cardio-Oncology Society (IC-OS). Eur. Heart J..

[234] Francis N. (2014). The need for routine monitoring of cardiac function in patients receiving 5-fluorouracil infusion. Clin. J. Oncol. Nurs..

[235] Upshaw J.N., O’Neill A., Carver J.R., Dimond E.P., Denlinger C.S., Kircher S.M., Wagner L.I., Ky B., Brell J.M. (2019). Fluoropyrimidine Cardiotoxicity: Time for a Contemporaneous Appraisal. Clin. Color. Cancer.

[236] Murphy S.P., Ibrahim N.E., Januzzi J.L. (2020). Heart Failure with Reduced Ejection Fraction: A Review. JAMA.

[237] Li C., Ngorsuraches S., Chou C., Chen L., Qian J. (2021). Risk Factors of Fluoropyrimidine Induced Cardiotoxicity among Cancer Patients: A Systematic Review and Meta-analysis. Crit. Rev. Oncol. Hematol..

[238] Albrektsen G., Heuch I., Løchen M.-L., Thelle D.S., Wilsgaard T., Njølstad I., Bønaa K.H. (2017). Risk of incident myocardial infarction by gender: Interactions with serum lipids, blood pressure and smoking. Atherosclerosis.

[239] Pallet N., Hamdane S., Garinet S., Blons H., Zaanan A., Paillaud E., Taieb J., Laprevote O., Loriot M.-A., Narjoz C. (2020). A comprehensive population-based study comparing the phenotype and genotype in a pretherapeutic screen of dihydropyrimidine dehydrogenase deficiency. Br. J. Cancer.

[240] Kelly C., Bhuva N., Harrison M., Buckley A., Saunders M. (2013). Use of raltitrexed as an alternative to 5-fluorouracil and capecitabine in cancer patients with cardiac history. Eur. J. Cancer.

