# Supplementary material for: Redefining the Incidence and Profile of Fluoropyrimidine-Associated Cardiotoxicity in Cancer Patients: A Systematic Review and Meta-Analysis
Source: Pharmaceuticals (Basel). 2023 Mar 30;16(4):510. doi: 10.3390/ph16040510 (PMC10146083; doi:10.3390/ph16040510)

Study	Events	Total	Proportion	95%−CI	Weight
Abdel−Rahman O 2019	255	3223	0.0791	[0.0700; 0.0890]	0.6%
Ackland SP 2001−A1	15	218	0.0688	[0.0390; 0.1109]	0.6%
Ackland SP 2001−A2	1	236	0.0042	[0.0001; 0.0234]	0.6%
Aimar G 2020	20	135	0.1481	[0.0929; 0.2195]	0.5%
Akhtar SS 1993	8	100	0.0800	[0.0352; 0.1516]	0.5%
Alderson D 2017−A1	15	446	0.0336	[0.0189; 0.0549]	0.6%
Alderson D 2017−A2	20	441	0.0454	[0.0279; 0.0692]	0.6%
Alonso MC 1995−A1	3	50	0.0600	[0.0125; 0.1655]	0.5%
Alonso MC 1995−A2	6	50	0.1200	[0.0453; 0.2431]	0.5%
Andersson M 1999−A1	1	161	0.0062	[0.0002; 0.0341]	0.5%
Andersson M 1999−A2	0	181	0.0000	[0.0000; 0.0202]	0.6%
Ardavanis A 2008	2	34	0.0588	[0.0072; 0.1968]	0.4%
Baghi M 2006	1	24	0.0417	[0.0011; 0.2112]	0.4%
Balloni L 2000	0	25	0.0000	[0.0000; 0.1372]	0.4%
Bascioni R 1997	2	46	0.0435	[0.0053; 0.1484]	0.4%
Bathe OF 2009	1	35	0.0286	[0.0007; 0.1492]	0.4%
Baur M 2010	4	71	0.0563	[0.0156; 0.1380]	0.5%
Becouarn YH 1996	4	86	0.0465	[0.0128; 0.1148]	0.5%
Berruti A 2005	2	53	0.0377	[0.0046; 0.1298]	0.5%
Berruti A 2005	5	61	0.0820	[0.0272; 0.1810]	0.5%
Birkenhake S 1999	0	25	0.0000	[0.0000; 0.1372]	0.4%
Blajman C 1999	7	85	0.0824	[0.0338; 0.1623]	0.5%
Blijham GH 1996	3	312	0.0096	[0.0020; 0.0278]	0.6%
Blum JL 1999	0	162	0.0000	[0.0000; 0.0225]	0.5%
Blum JL 2001	0	74	0.0000	[0.0000; 0.0486]	0.5%
Buzdar AU 2007−A1	2	19	0.1053	[0.0130; 0.3314]	0.3%
Buzdar AU 2007−A2	0	45	0.0000	[0.0000; 0.0787]	0.4%
Carmo−Pereira J 1993	0	36	0.0000	[0.0000; 0.0974]	0.4%
Carmo−Pereira J 1995	0	38	0.0000	[0.0000; 0.0925]	0.4%
Cetin B 2012	0	203	0.0000	[0.0000; 0.0180]	0.6%
Ceyhan C 2005	2	37	0.0541	[0.0066; 0.1819]	0.4%
Chen EY 2018	8	47	0.1702	[0.0765; 0.3081]	0.4%
Chua YJ 2010	7	105	0.0667	[0.0272; 0.1325]	0.5%
Citron ML 1992	4	86	0.0465	[0.0128; 0.1148]	0.5%
Colozza M 1996	0	28	0.0000	[0.0000; 0.1234]	0.4%
Conroy T 2021	1	163	0.0061	[0.0002; 0.0337]	0.5%
Daniele B 2003	2	34	0.0588	[0.0072; 0.1968]	0.4%
de Forni M 1992	28	367	0.0763	[0.0513; 0.1084]	0.6%
Delaloge S 2020	7	628	0.0111	[0.0045; 0.0228]	0.6%
Dencausse Y 2001	1	21	0.0476	[0.0012; 0.2382]	0.3%
Depondt J 1993	2	150	0.0133	[0.0016; 0.0473]	0.5%
Dipetrillo T 2012	1	26	0.0385	[0.0010; 0.1964]	0.4%
Doci R 1996	1	35	0.0286	[0.0007; 0.1492]	0.4%
Dyhl−Polk A 2020(1)	28	108	0.2593	[0.1797; 0.3525]	0.5%
Dyhl−Polk A 2020(2)−A1	52	995	0.0523	[0.0393; 0.0680]	0.6%
Dyhl−Polk A 2020(2)−A2	51	1241	0.0411	[0.0307; 0.0537]	0.6%
ECOG−E1103/ Li TH 2004/2012	11	68	0.1618	[0.0836; 0.2710]	0.5%
ECOG−E2200/Giantonio BJ 2006	22	87	0.2529	[0.1658; 0.3575]	0.5%
ECOG−E2204/ Berlin DJ 2006/2018	18	130	0.1385	[0.0842; 0.2100]	0.5%
ECOG−E3204/ Landry JC 2006/2015	8	55	0.1455	[0.0650; 0.2666]	0.5%
ECOG−E3205/ Garg MK 2007/2017	2	62	0.0323	[0.0039; 0.1117]	0.5%
ECOG−E4203/ Meropol NJ 2005/2018	13	205	0.0634	[0.0342; 0.1060]	0.6%
Elomaa I 2003	2	40	0.0500	[0.0061; 0.1692]	0.4%
Emmanouilides C 2006	2	53	0.0377	[0.0046; 0.1298]	0.5%
Erman M 2005	7	88	0.0795	[0.0326; 0.1570]	0.5%
Eskilsson J 1988	14	76	0.1842	[0.1045; 0.2897]	0.5%
Eskilsson J 1990	8	58	0.1379	[0.0615; 0.2538]	0.5%
Feliu J 2005	1	51	0.0196	[0.0005; 0.1045]	0.5%
Fetting JH 1998−A1	15	307	0.0489	[0.0276; 0.0793]	0.6%
Fetting JH 1998−A2	31	309	0.1003	[0.0692; 0.1394]	0.6%
Gebbia V 1996	2	33	0.0606	[0.0074; 0.2023]	0.4%
Geyer CE 2006−A1	4	163	0.0245	[0.0067; 0.0616]	0.5%
Geyer CE 2006−A2	0	161	0.0000	[0.0000; 0.0227]	0.5%
Gianni L 2005	151	1233	0.1225	[0.1047; 0.1421]	0.6%
Giordano KF 2006	8	44	0.1818	[0.0819; 0.3271]	0.4%
Giuliani F 2008	1	41	0.0244	[0.0006; 0.1286]	0.4%
Gradishar W 1991	15	244	0.0615	[0.0348; 0.0994]	0.6%
Greil R 2011	6	293	0.0205	[0.0076; 0.0440]	0.6%
Haarstad H 1994	3	30	0.1000	[0.0211; 0.2653]	0.4%
Haas NB 1995	2	38	0.0526	[0.0064; 0.1775]	0.4%
Hainsworth JD 1996	2	46	0.0435	[0.0053; 0.1484]	0.4%
Harbeck N 2017	37	102	0.3627	[0.2698; 0.4639]	0.5%
Hartung G 1996−A1	0	16	0.0000	[0.0000; 0.2059]	0.3%
Hartung G 1996−A2	1	29	0.0345	[0.0009; 0.1776]	0.4%
Hartung G 2001	2	51	0.0392	[0.0048; 0.1346]	0.5%
Hasbini A 1999	2	44	0.0455	[0.0056; 0.1547]	0.4%
Havsteen H 1989	1	11	0.0909	[0.0023; 0.4128]	0.2%
Hayashi Y 2019	13	80	0.1625	[0.0895; 0.2618]	0.5%
Hitt R 2002	4	70	0.0571	[0.0158; 0.1399]	0.5%
Hofheinz RD 2021	1	56	0.0179	[0.0005; 0.0955]	0.5%
Hu W 2010−A1	2	34	0.0588	[0.0072; 0.1968]	0.4%
Hu W 2010−A2	9	37	0.2432	[0.1177; 0.4120]	0.4%
Jassem J 1993	20	189	0.1058	[0.0658; 0.1587]	0.6%
Jassem J 2001	16	123	0.1301	[0.0762; 0.2026]	0.5%
Jensen SA 2006	29	668	0.0434	[0.0293; 0.0618]	0.6%
Jensen SA 2011	9	106	0.0849	[0.0396; 0.1551]	0.5%
Jeremic B 1999	12	80	0.1500	[0.0800; 0.2474]	0.5%
Jin X 2019	38	129	0.2946	[0.2176; 0.3812]	0.5%
Joensuu H 2009−A1	8	115	0.0696	[0.0305; 0.1325]	0.5%
Joensuu H 2009−A2	12	116	0.1034	[0.0546; 0.1737]	0.5%
Kachnic LA 2022	2	52	0.0385	[0.0047; 0.1321]	0.5%
Kato K 2010	23	76	0.3026	[0.2025; 0.4187]	0.5%
Katona C 1998	4	96	0.0417	[0.0115; 0.1033]	0.5%
Keene KS 2005	1	28	0.0357	[0.0009; 0.1835]	0.4%
Kerr RS 2016−A1	73	973	0.0750	[0.0593; 0.0934]	0.6%
Kerr RS 2016−A2	75	968	0.0775	[0.0614; 0.0962]	0.6%
Khan MA 2012	60	301	0.1993	[0.1557; 0.2490]	0.6%
Klastersky J 1994	25	182	0.1374	[0.0909; 0.1961]	0.6%
Klautke G 2006	1	28	0.0357	[0.0009; 0.1835]	0.4%
Koca D 2011	18	52	0.3462	[0.2197; 0.4909]	0.5%
Kok TC 1996	1	29	0.0345	[0.0009; 0.1776]	0.4%
Kolaric K 1995	11	237	0.0464	[0.0234; 0.0815]	0.6%
Kosmas C 2008	26	644	0.0404	[0.0265; 0.0586]	0.6%
Kroning KC 1997	0	27	0.0000	[0.0000; 0.1277]	0.4%
Kuzel TM 1993	2	22	0.0909	[0.0112; 0.2916]	0.3%
Kwakman JJM 2017	120	1973	0.0608	[0.0507; 0.0723]	0.6%
Labianca R 1982	17	1083	0.0157	[0.0092; 0.0250]	0.6%
Langer CJ 1993	2	40	0.0500	[0.0061; 0.1692]	0.4%
Leicher LW 2017	5	86	0.0581	[0.0191; 0.1305]	0.5%
Leonardi V 1995	2	34	0.0588	[0.0072; 0.1968]	0.4%
Lestuzzi C 2014	37	358	0.1034	[0.0738; 0.1396]	0.6%
Lestuzzi C 2016	32	192	0.1667	[0.1169; 0.2271]	0.6%
Levine MN 2005−A1	1	359	0.0028	[0.0001; 0.0154]	0.6%
Levine MN 2005−A2	4	351	0.0114	[0.0031; 0.0289]	0.6%
Levine MN 1993	2	288	0.0069	[0.0008; 0.0249]	0.6%
Lin JK 2000	0	40	0.0000	[0.0000; 0.0881]	0.4%
Lombardi P 2019	19	101	0.1881	[0.1172; 0.2781]	0.5%
Lopez L 2012	8	240	0.0333	[0.0145; 0.0646]	0.6%
Lordick F 2005	1	48	0.0208	[0.0005; 0.1107]	0.4%
Macdonald JS 1995	1	90	0.0111	[0.0003; 0.0604]	0.5%
Machiels JP 2007	1	40	0.0250	[0.0006; 0.1316]	0.4%
Mambrini A 2006	2	211	0.0095	[0.0011; 0.0338]	0.6%
Mammoliti S 1996	1	24	0.0417	[0.0011; 0.2112]	0.4%
Mantovani G 1998−A1	2	16	0.1250	[0.0155; 0.3835]	0.3%
Mantovani G 1998−A2	0	17	0.0000	[0.0000; 0.1951]	0.3%
Martin M 2003−A1	1	505	0.0020	[0.0001; 0.0110]	0.6%
Martin M 2003−A2	10	480	0.0208	[0.0100; 0.0380]	0.6%
Martin M 2012	5	88	0.0568	[0.0187; 0.1276]	0.5%
Mayer IA 2021	2	198	0.0101	[0.0012; 0.0360]	0.6%
Mayer RJ 2015	3	534	0.0056	[0.0012; 0.0163]	0.6%
Meydan N 2005	9	231	0.0390	[0.0180; 0.0727]	0.6%
Meyer CC 1997	9	483	0.0186	[0.0086; 0.0351]	0.6%
Meyerhardt JA 2021	20	2278	0.0088	[0.0054; 0.0135]	0.6%
Michalaki V 2010	0	29	0.0000	[0.0000; 0.1194]	0.4%
Milano G 1999	1	19	0.0526	[0.0013; 0.2603]	0.3%
Moertel CG 1998	3	109	0.0275	[0.0057; 0.0783]	0.5%
Natoli C 2007	0	44	0.0000	[0.0000; 0.0804]	0.4%
Neri B 1993	5	35	0.1429	[0.0481; 0.3026]	0.4%
Neri B 2007	0	36	0.0000	[0.0000; 0.0974]	0.4%
Ng M 2005	10	153	0.0654	[0.0318; 0.1169]	0.5%
Nishimura Y 2012−A1	5	42	0.1190	[0.0398; 0.2563]	0.4%
Nishimura Y 2012−A2	5	45	0.1111	[0.0371; 0.2405]	0.4%
Osawa S 2009	9	33	0.2727	[0.1330; 0.4552]	0.4%
Osterlund P 2020	5	141	0.0355	[0.0116; 0.0808]	0.5%
Oztop I 2004	0	22	0.0000	[0.0000; 0.1544]	0.3%
Papadimitriou CA 1998	1	24	0.0417	[0.0011; 0.2112]	0.4%
Peiffert D 2001	0	80	0.0000	[0.0000; 0.0451]	0.5%
Peng J 2018	161	527	0.3055	[0.2664; 0.3468]	0.6%
Perit P 1991−A1	7	31	0.2258	[0.0959; 0.4110]	0.4%
Perit P 1991−A2	6	29	0.2069	[0.0799; 0.3972]	0.4%
Petrini L 2012	3	39	0.0769	[0.0162; 0.2087]	0.4%
Pisano C 2009	2	36	0.0556	[0.0068; 0.1866]	0.4%
Polk A 2016	22	452	0.0487	[0.0308; 0.0728]	0.6%
Pottage A 1978	4	140	0.0286	[0.0078; 0.0715]	0.5%
Raber I 2019	8	177	0.0452	[0.0197; 0.0871]	0.6%
Rajendra A 2020	16	246	0.0650	[0.0376; 0.1035]	0.6%
Rapidis AD 2006	1	42	0.0238	[0.0006; 0.1257]	0.4%
Recchia F 2001	3	28	0.1071	[0.0227; 0.2823]	0.4%
Rezkalla S 1989	5	25	0.2000	[0.0683; 0.4070]	0.4%
Riccardi A 2001	3	69	0.0435	[0.0091; 0.1218]	0.5%
Rivera F 2019	3	45	0.0667	[0.0140; 0.1827]	0.4%
Rossi D 2007	1	37	0.0270	[0.0007; 0.1416]	0.4%
Samonigg H 1990	0	21	0.0000	[0.0000; 0.1611]	0.3%
Sasamoto R 2007−A1	5	68	0.0735	[0.0243; 0.1633]	0.5%
Sasamoto R 2007−A2	1	68	0.0147	[0.0004; 0.0792]	0.5%
Schlumberger M 1991	3	14	0.2143	[0.0466; 0.5080]	0.3%
Schober C 1993	13	390	0.0333	[0.0179; 0.0563]	0.6%
Seitz JF 1998	0	24	0.0000	[0.0000; 0.1425]	0.4%
Skof E 2009−A1	1	41	0.0244	[0.0006; 0.1286]	0.4%
Skof E 2009−A2	2	46	0.0435	[0.0053; 0.1484]	0.4%
Smaradottir A 2005	5	12	0.4167	[0.1517; 0.7233]	0.3%
Souglakos J 2012−A1	7	166	0.0422	[0.0171; 0.0850]	0.6%
Souglakos J 2012−A2	6	167	0.0359	[0.0133; 0.0766]	0.6%
Susvnjar S 1999	1	19	0.0526	[0.0013; 0.2603]	0.3%
Taylor SG 1997	1	78	0.0128	[0.0003; 0.0694]	0.5%
Thatai LC 2006	0	39	0.0000	[0.0000; 0.0903]	0.4%
Tominaga T 1998	1	20	0.0500	[0.0013; 0.2487]	0.3%
Tonyali O 2013	1	37	0.0270	[0.0007; 0.1416]	0.4%
Torrisi R 2010	2	32	0.0625	[0.0077; 0.2081]	0.4%
Tsavaris N 2002	17	427	0.0398	[0.0234; 0.0630]	0.6%
Tsavaris N 2005	20	522	0.0383	[0.0236; 0.0586]	0.6%
Tsibiribi P 2006	16	1350	0.0119	[0.0068; 0.0192]	0.6%
Turan T 2017	4	32	0.1250	[0.0351; 0.2899]	0.4%
Vaishampayan UN 2002	1	32	0.0312	[0.0008; 0.1622]	0.4%
Valero V 1999	3	41	0.0732	[0.0154; 0.1992]	0.4%
Van Cutsem E 2002−A1	20	593	0.0337	[0.0207; 0.0516]	0.6%
Van Cutsem E 2002−A2	19	596	0.0319	[0.0193; 0.0493]	0.6%
Van Cutsem E 2002−A3	7	236	0.0297	[0.0120; 0.0602]	0.6%
Velenik V 2006	1	57	0.0175	[0.0004; 0.0939]	0.5%
Villar−Grimalt A 1994	3	18	0.1667	[0.0358; 0.4142]	0.3%
Wang WS 1998	1	41	0.0244	[0.0006; 0.1286]	0.4%
Wardley AM 2010	13	112	0.1161	[0.0633; 0.1903]	0.5%
Warner E 1999	4	30	0.1333	[0.0376; 0.3072]	0.4%
Weidmann B 1995	6	231	0.0260	[0.0096; 0.0557]	0.6%
Wildiers H 2010−A1	0	53	0.0000	[0.0000; 0.0672]	0.5%
Wildiers H 2010−A2	0	50	0.0000	[0.0000; 0.0711]	0.5%
Winther SB 2016	2	71	0.0282	[0.0034; 0.0981]	0.5%
Yamaguchi K 2022	1	100	0.0100	[0.0003; 0.0545]	0.5%
Yamamoto D 2007	1	59	0.0169	[0.0004; 0.0909]	0.5%
Yang H 2018−A1	3	30	0.1000	[0.0211; 0.2653]	0.4%
Yang H 2018−A2	2	30	0.0667	[0.0082; 0.2207]	0.4%
Ychou M 1996	1	28	0.0357	[0.0009; 0.1835]	0.4%
Yen CC 1997	9	13	0.6923	[0.3857; 0.9091]	0.3%
Yerushalmi R 2006−A1	1	43	0.0233	[0.0006; 0.1229]	0.4%
Yerushalmi R 2006−A2	0	46	0.0000	[0.0000; 0.0771]	0.4%
Yilmaz U 2007	8	27	0.2963	[0.1375; 0.5018]	0.4%
Zamagni C 1991	3	30	0.1000	[0.0211; 0.2653]	0.4%
Zambelli A 2000	0	48	0.0000	[0.0000; 0.0740]	0.4%
Zaniboni A 1991	1	30	0.0333	[0.0008; 0.1722]	0.4%
Zhang P 2017−A1	4	267	0.0150	[0.0041; 0.0379]	0.6%
Zhang P 2017−A2	2	130	0.0154	[0.0019; 0.0545]	0.5%
**Random effects model**		**40170**	**0.0504**	**[0.0421; 0.0594]**	**100.0%**

Heterogeneity: $I^2 = 90\%$, $\tau^2 = 0.0141$, $p < 0.01$

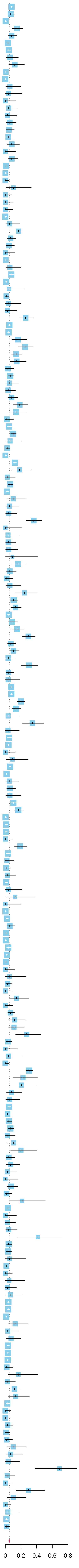

Supplement: Supplementary file 1 [file pharmaceuticals-16-00510-s001.zip › Figure S2 Forest plot of the all grade.pdf]
