# Supplementary material for: Redefining the Incidence and Profile of Fluoropyrimidine-Associated Cardiotoxicity in Cancer Patients: A Systematic Review and Meta-Analysis
Source: Pharmaceuticals (Basel). 2023 Mar 30;16(4):510. doi: 10.3390/ph16040510 (PMC10146083; doi:10.3390/ph16040510)

Study	Events	Total	Proportion	95%−CI	Weight
Mayer IA 2021	1	198	0.0051	[0.0001; 0.0278]	0.9%
Chakravarthy AB 2020−A1	1	176	0.0057	[0.0001; 0.0312]	0.9%
Chakravarthy AB 2020−A2	3	179	0.0168	[0.0035; 0.0482]	0.9%
Dyhl−Polk A 2020(1)	11	108	0.1019	[0.0520; 0.1749]	0.8%
Osterlund P 2020	0	141	0.0000	[0.0000; 0.0258]	0.8%
Delaloge S 2020	3	628	0.0048	[0.0010; 0.0139]	1.1%
Dyhl−Polk A 2020(2)−A1	20	995	0.0201	[0.0123; 0.0309]	1.1%
Dyhl−Polk A 2020(2)−A2	20	1241	0.0161	[0.0099; 0.0248]	1.1%
Gibson MK 2019	6	22	0.2727	[0.1073; 0.5022]	0.4%
Jin X 2019	12	129	0.0930	[0.0490; 0.1569]	0.8%
Rivera F 2019	1	45	0.0222	[0.0006; 0.1177]	0.5%
Primrose JN 2019	5	213	0.0235	[0.0077; 0.0539]	0.9%
Chen EY 2018	6	47	0.1277	[0.0483; 0.2574]	0.6%
Yang H 2018−A1	0	30	0.0000	[0.0000; 0.1157]	0.4%
Yang H 2018−A2	0	30	0.0000	[0.0000; 0.1157]	0.4%
Alderson D 2017−A1	2	446	0.0045	[0.0005; 0.0161]	1.0%
Alderson D 2017−A2	3	441	0.0068	[0.0014; 0.0198]	1.0%
Kwakman JJM 2017	47	1973	0.0238	[0.0176; 0.0316]	1.1%
Harbeck N 2017	7	102	0.0686	[0.0280; 0.1363]	0.8%
Kerr RS 2016−A1	33	973	0.0339	[0.0235; 0.0473]	1.1%
Kerr RS 2016−A2	27	968	0.0279	[0.0185; 0.0403]	1.1%
Winther SB 2016	2	71	0.0282	[0.0034; 0.0981]	0.7%
Mayer RJ 2015	1	534	0.0019	[0.0000; 0.0104]	1.1%
van der Sluis PC 2015−A1	7	93	0.0753	[0.0308; 0.1490]	0.7%
van der Sluis PC 2015−A2	0	38	0.0000	[0.0000; 0.0925]	0.5%
Tonyali O 2013	1	37	0.0270	[0.0007; 0.1416]	0.5%
Okines AFC 2013−A1	1	101	0.0099	[0.0003; 0.0539]	0.8%
Okines AFC 2013−A2	4	99	0.0404	[0.0111; 0.1002]	0.8%
Uygun K 2013	0	132	0.0000	[0.0000; 0.0276]	0.8%
Mackey JR 2013	17	736	0.0231	[0.0135; 0.0367]	1.1%
Cetin B 2012	0	203	0.0000	[0.0000; 0.0180]	0.9%
Lang I 2012	0	277	0.0000	[0.0000; 0.0132]	1.0%
Nishimura Y 2012−A1	5	42	0.1190	[0.0398; 0.2563]	0.5%
Nishimura Y 2012−A2	3	45	0.0667	[0.0140; 0.1827]	0.5%
Martin M 2012	2	88	0.0227	[0.0028; 0.0797]	0.7%
Petrini L 2012	3	39	0.0769	[0.0162; 0.2087]	0.5%
Dipetrillo T 2012	1	26	0.0385	[0.0010; 0.1964]	0.4%
Greil R 2011	3	293	0.0102	[0.0021; 0.0296]	1.0%
Robert NJ 2011−A1	1	201	0.0050	[0.0001; 0.0274]	0.9%
Robert NJ 2011−A2	6	404	0.0149	[0.0055; 0.0320]	1.0%
Kato K 2010	12	76	0.1579	[0.0843; 0.2596]	0.7%
Wildiers H 2010−A1	0	53	0.0000	[0.0000; 0.0672]	0.6%
Wildiers H 2010−A2	0	50	0.0000	[0.0000; 0.0711]	0.6%
Masi G 2010	2	57	0.0351	[0.0043; 0.1211]	0.6%
Michalaki V 2010	0	29	0.0000	[0.0000; 0.1194]	0.4%
Baur M 2010	1	71	0.0141	[0.0004; 0.0760]	0.7%
Hu W 2010−A1	0	34	0.0000	[0.0000; 0.1028]	0.5%
Hu W 2010−A2	1	37	0.0270	[0.0007; 0.1416]	0.5%
Osawa S 2009	2	33	0.0606	[0.0074; 0.2023]	0.5%
Pisano C 2009	2	36	0.0556	[0.0068; 0.1866]	0.5%
Bathe OF 2009	1	35	0.0286	[0.0007; 0.1492]	0.5%
Skof E 2009−A1	1	41	0.0244	[0.0006; 0.1286]	0.5%
Skof E 2009−A2	3	46	0.0652	[0.0137; 0.1790]	0.6%
Giuliani F 2008	1	41	0.0244	[0.0006; 0.1286]	0.5%
Ardavanis A 2008	1	34	0.0294	[0.0007; 0.1533]	0.5%
Rothenberg ML 2008−A1	2	311	0.0064	[0.0008; 0.0230]	1.0%
Rothenberg ML 2008−A2	2	308	0.0065	[0.0008; 0.0233]	1.0%
Yamamoto D 2007	0	59	0.0000	[0.0000; 0.0606]	0.6%
Natoli C 2007	0	44	0.0000	[0.0000; 0.0804]	0.5%
Neri B 2007	0	36	0.0000	[0.0000; 0.0974]	0.5%
Machiels JP 2007	1	40	0.0250	[0.0006; 0.1316]	0.5%
Sasamoto R 2007−A1	4	68	0.0588	[0.0163; 0.1438]	0.7%
Sasamoto R 2007−A2	1	68	0.0147	[0.0004; 0.0792]	0.7%
ECOG−E3205/ Garg MK 2007/2017	2	62	0.0323	[0.0039; 0.1117]	0.6%
Emmanouilides C 2006	2	53	0.0377	[0.0046; 0.1298]	0.6%
Thatai LC 2006	0	39	0.0000	[0.0000; 0.0903]	0.5%
Jensen SA 2006−A1	4	214	0.0187	[0.0051; 0.0472]	0.9%
Jensen SA 2006−A2	3	81	0.0370	[0.0077; 0.1044]	0.7%
Jensen SA 2006−A3	17	352	0.0483	[0.0284; 0.0762]	1.0%
Jensen SA 2006−A4	1	10	0.1000	[0.0025; 0.4450]	0.2%
Jensen SA 2006−A5	2	11	0.1818	[0.0228; 0.5178]	0.2%
Velenik V 2006	1	57	0.0175	[0.0004; 0.0939]	0.6%
Rapidis AD 2006	1	42	0.0238	[0.0006; 0.1257]	0.5%
Klautke G 2006	1	28	0.0357	[0.0009; 0.1835]	0.4%
Giordano KF 2006	7	44	0.1591	[0.0664; 0.3007]	0.5%
Jatoi A 2006	4	46	0.0870	[0.0242; 0.2079]	0.6%
Baghi M 2006	1	24	0.0417	[0.0011; 0.2112]	0.4%
Gianni L 2005	6	1233	0.0049	[0.0018; 0.0106]	1.1%
Bontenbal M 2005	6	107	0.0561	[0.0209; 0.1181]	0.8%
Lordick F 2005	1	48	0.0208	[0.0005; 0.1107]	0.6%
Berruti A 2005	1	61	0.0164	[0.0004; 0.0880]	0.6%
Erman M 2005	0	88	0.0000	[0.0000; 0.0411]	0.7%
Berruti A 2005	1	53	0.0189	[0.0005; 0.1007]	0.6%
ECOG−E1103/ Li TH 2004/2012	1	68	0.0147	[0.0004; 0.0792]	0.7%
Oztop I 2004	0	22	0.0000	[0.0000; 0.1544]	0.4%
Martin M 2003−A1	0	505	0.0000	[0.0000; 0.0073]	1.1%
Martin M 2003−A2	3	480	0.0063	[0.0013; 0.0182]	1.0%
Daniele B 2003	2	34	0.0588	[0.0072; 0.1968]	0.5%
Elomaa I 2003	2	40	0.0500	[0.0061; 0.1692]	0.5%
ECOG−E3201 2003/2018−A1	2	59	0.0339	[0.0041; 0.1171]	0.6%
ECOG−E3201 2003/2018−A2	0	58	0.0000	[0.0000; 0.0616]	0.6%
ECOG−E3201 2003/2018−A3	1	62	0.0161	[0.0004; 0.0866]	0.6%
Hitt R 2002	1	70	0.0143	[0.0004; 0.0770]	0.7%
Vaishampayan UN 2002	1	32	0.0312	[0.0008; 0.1622]	0.4%
Van Cutsem E 2002−A1	4	593	0.0067	[0.0018; 0.0172]	1.1%
Van Cutsem E 2002−A2	5	596	0.0084	[0.0027; 0.0195]	1.1%
Van Cutsem E 2002−A3	0	236	0.0000	[0.0000; 0.0155]	1.0%
ECOG−E3200/ Giantonio BJ 2001/2007	22	808	0.0272	[0.0171; 0.0409]	1.1%
Hartung G 2001	2	51	0.0392	[0.0048; 0.1346]	0.6%
Dencausse Y 2001	1	21	0.0476	[0.0012; 0.2382]	0.3%
Recchia F 2001	0	28	0.0000	[0.0000; 0.1234]	0.4%
Riccardi A 2001	1	69	0.0145	[0.0004; 0.0781]	0.7%
Piccart MJ 2001	0	255	0.0000	[0.0000; 0.0144]	1.0%
Peiffert D 2001	2	80	0.0250	[0.0030; 0.0874]	0.7%
Pienkowski T 2001	3	65	0.0462	[0.0096; 0.1290]	0.6%
Blum JL 2001	0	74	0.0000	[0.0000; 0.0486]	0.7%
Hoff PM 2001−A1	2	302	0.0066	[0.0008; 0.0237]	1.0%
Hoff PM 2001−A2	3	303	0.0099	[0.0020; 0.0287]	1.0%
Lin JK 2000	0	40	0.0000	[0.0000; 0.0881]	0.5%
Zambelli A 2000	0	48	0.0000	[0.0000; 0.0740]	0.6%
Balloni L 2000	0	25	0.0000	[0.0000; 0.1372]	0.4%
Susvnjar S 1999	0	19	0.0000	[0.0000; 0.1765]	0.3%
Valero V 1999	1	41	0.0244	[0.0006; 0.1286]	0.5%
Hasbini A 1999	1	44	0.0227	[0.0006; 0.1202]	0.5%
Blum JL 1999	0	162	0.0000	[0.0000; 0.0225]	0.9%
Blajman C 1999	0	85	0.0000	[0.0000; 0.0425]	0.7%
Birkenhake S 1999	0	25	0.0000	[0.0000; 0.1372]	0.4%
Milano G 1999	1	19	0.0526	[0.0013; 0.2603]	0.3%
Mantovani G 1998−A1	1	16	0.0625	[0.0016; 0.3023]	0.3%
Mantovani G 1998−A2	0	17	0.0000	[0.0000; 0.1951]	0.3%
Papadimitriou CA 1998	1	24	0.0417	[0.0011; 0.2112]	0.4%
Fetting JH 1998−A1	1	307	0.0033	[0.0001; 0.0180]	1.0%
Fetting JH 1998−A2	5	309	0.0162	[0.0053; 0.0374]	1.0%
Seitz JF 1998	0	24	0.0000	[0.0000; 0.1425]	0.4%
Wang WS 1998	1	41	0.0244	[0.0006; 0.1286]	0.5%
Taylor SG 1997	1	78	0.0128	[0.0003; 0.0694]	0.7%
Yen CC 1997	4	13	0.3077	[0.0909; 0.6143]	0.2%
Kroning KC 1997	0	27	0.0000	[0.0000; 0.1277]	0.4%
Ychou M 1996	1	28	0.0357	[0.0009; 0.1835]	0.4%
Kok TC 1996	0	29	0.0000	[0.0000; 0.1194]	0.4%
Gebbia V 1996	0	33	0.0000	[0.0000; 0.1058]	0.5%
Hainsworth JD 1996	2	46	0.0435	[0.0053; 0.1484]	0.6%
Colozza M 1996	0	28	0.0000	[0.0000; 0.1234]	0.4%
Mammoliti S 1996	1	24	0.0417	[0.0011; 0.2112]	0.4%
Becouarn YH 1996	3	86	0.0349	[0.0073; 0.0986]	0.7%
Macdonald JS 1995	1	90	0.0111	[0.0003; 0.0604]	0.7%
Leonardi V 1995	2	34	0.0588	[0.0072; 0.1968]	0.5%
Carmo−Pereira J 1995	0	38	0.0000	[0.0000; 0.0925]	0.5%
Haas NB 1995	2	38	0.0526	[0.0064; 0.1775]	0.5%
Klastersky J 1994	20	182	0.1099	[0.0684; 0.1646]	0.9%
Morere JF 1994	0	109	0.0000	[0.0000; 0.0333]	0.8%
Langer CJ 1993	2	40	0.0500	[0.0061; 0.1692]	0.5%
Carmo−Pereira J 1993	0	36	0.0000	[0.0000; 0.0974]	0.5%
Neri B 1993	2	35	0.0571	[0.0070; 0.1916]	0.5%
Jassem J 1993	7	189	0.0370	[0.0150; 0.0748]	0.9%
Keefe DL 1993	5	910	0.0055	[0.0018; 0.0128]	1.1%
Kuzel TM 1993	2	22	0.0909	[0.0112; 0.2916]	0.4%
Citron ML 1992	3	86	0.0349	[0.0073; 0.0986]	0.7%
Perit P 1991−A1	0	31	0.0000	[0.0000; 0.1122]	0.4%
Perit P 1991−A2	0	29	0.0000	[0.0000; 0.1194]	0.4%
Zamagni C 1991	0	30	0.0000	[0.0000; 0.1157]	0.4%
Samonigg H 1990	0	21	0.0000	[0.0000; 0.1611]	0.3%
**Random effects model**		**25273**	**0.0150**	**[0.0109; 0.0196]**	**100.0%**

Heterogeneity: I^2 = 70%, τ^2 = 0.0049, p < 0.01

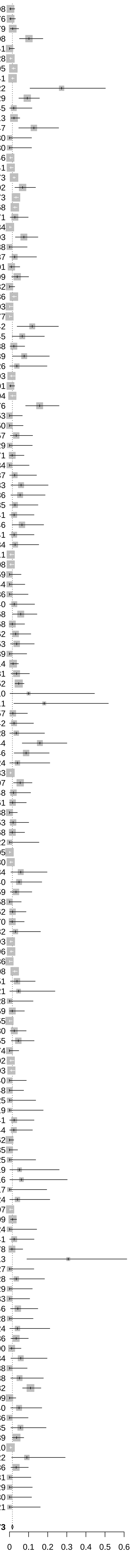

Supplement: Supplementary file 1 [file pharmaceuticals-16-00510-s001.zip › Figure S3 Forest plot of grade 3 or higher.pdf]
