# Supplementary material for: Redefining the Incidence and Profile of Fluoropyrimidine-Associated Cardiotoxicity in Cancer Patients: A Systematic Review and Meta-Analysis
Source: Pharmaceuticals (Basel). 2023 Mar 30;16(4):510. doi: 10.3390/ph16040510 (PMC10146083; doi:10.3390/ph16040510)

Study	Events	Total	Proportion	95%−CI	Weight
Mayer IA 2021	1	198	0.0051	[0.0001; 0.0278]	1.2%
Dyhl−Polk A 2020(1)	17	108	0.1574	[0.0945; 0.2400]	1.1%
Osterlund P 2020	5	141	0.0355	[0.0116; 0.0808]	1.1%
Delaloge S 2020	4	628	0.0064	[0.0017; 0.0162]	1.3%
Dyhl−Polk A 2020(2)−A1	32	995	0.0322	[0.0221; 0.0451]	1.3%
Dyhl−Polk A 2020(2)−A2	31	1241	0.0250	[0.0170; 0.0353]	1.3%
Jin X 2019	26	129	0.2016	[0.1361; 0.2812]	1.1%
Rivera F 2019	2	45	0.0444	[0.0054; 0.1515]	0.9%
Chen EY 2018	2	47	0.0426	[0.0052; 0.1454]	0.9%
Yang H 2018−A1	3	30	0.1000	[0.0211; 0.2653]	0.7%
Yang H 2018−A2	2	30	0.0667	[0.0082; 0.2207]	0.7%
Alderson D 2017−A1	13	446	0.0291	[0.0156; 0.0493]	1.2%
Alderson D 2017−A2	17	441	0.0385	[0.0226; 0.0610]	1.2%
Kwakman JJM 2017	73	1973	0.0370	[0.0291; 0.0463]	1.3%
Harbeck N 2017	30	102	0.2941	[0.2080; 0.3925]	1.1%
Kerr RS 2016−A1	40	973	0.0411	[0.0295; 0.0556]	1.3%
Kerr RS 2016−A2	48	968	0.0496	[0.0368; 0.0652]	1.3%
Mayer RJ 2015	2	534	0.0037	[0.0005; 0.0135]	1.3%
Cetin B 2012	0	203	0.0000	[0.0000; 0.0180]	1.2%
Nishimura Y 2012−A1	0	42	0.0000	[0.0000; 0.0841]	0.8%
Nishimura Y 2012−A2	2	45	0.0444	[0.0054; 0.1515]	0.9%
Martin M 2012	3	88	0.0341	[0.0071; 0.0964]	1.0%
Petrini L 2012	0	39	0.0000	[0.0000; 0.0903]	0.8%
Greil R 2011	3	293	0.0102	[0.0021; 0.0296]	1.2%
Kato K 2010	11	76	0.1447	[0.0745; 0.2442]	1.0%
Wildiers H 2010−A1	0	53	0.0000	[0.0000; 0.0672]	0.9%
Wildiers H 2010−A2	0	50	0.0000	[0.0000; 0.0711]	0.9%
Michalaki V 2010	0	29	0.0000	[0.0000; 0.1194]	0.7%
Baur M 2010	3	71	0.0423	[0.0088; 0.1186]	1.0%
Hu W 2010−A1	2	34	0.0588	[0.0072; 0.1968]	0.8%
Hu W 2010−A2	8	37	0.2162	[0.0983; 0.3821]	0.8%
Osawa S 2009	7	33	0.2121	[0.0898; 0.3891]	0.8%
Pisano C 2009	0	36	0.0000	[0.0000; 0.0974]	0.8%
Bathe OF 2009	0	35	0.0000	[0.0000; 0.1000]	0.8%
Giuliani F 2008	0	41	0.0000	[0.0000; 0.0860]	0.8%
Ardavanis A 2008	1	34	0.0294	[0.0007; 0.1533]	0.8%
Yamamoto D 2007	1	59	0.0169	[0.0004; 0.0909]	0.9%
Natoli C 2007	0	44	0.0000	[0.0000; 0.0804]	0.9%
Neri B 2007	0	36	0.0000	[0.0000; 0.0974]	0.8%
Machiels JP 2007	0	40	0.0000	[0.0000; 0.0881]	0.8%
Jensen SA 2006	2	668	0.0030	[0.0004; 0.0108]	1.3%
Rapidis AD 2006	0	42	0.0000	[0.0000; 0.0841]	0.8%
Giordano KF 2006	1	44	0.0227	[0.0006; 0.1202]	0.9%
Gianni L 2005	145	1233	0.1176	[0.1001; 0.1369]	1.3%
Lordick F 2005	0	48	0.0000	[0.0000; 0.0740]	0.9%
Berruti A 2005	4	61	0.0656	[0.0182; 0.1595]	1.0%
Erman M 2005	7	88	0.0795	[0.0326; 0.1570]	1.0%
Berruti A 2005	1	53	0.0189	[0.0005; 0.1007]	0.9%
ECOG−E1103/ Li TH 2004/2012	10	68	0.1471	[0.0728; 0.2539]	1.0%
Oztop I 2004	0	22	0.0000	[0.0000; 0.1544]	0.6%
Martin M 2003−A1	1	505	0.0020	[0.0001; 0.0110]	1.3%
Martin M 2003−A2	7	480	0.0146	[0.0059; 0.0298]	1.2%
Daniele B 2003	0	34	0.0000	[0.0000; 0.1028]	0.8%
Elomaa I 2003	0	40	0.0000	[0.0000; 0.0881]	0.8%
Hitt R 2002	3	70	0.0429	[0.0089; 0.1202]	1.0%
Vaishampayan UN 2002	0	32	0.0000	[0.0000; 0.1089]	0.8%
Van Cutsem E 2002−A1	16	593	0.0270	[0.0155; 0.0434]	1.3%
Van Cutsem E 2002−A2	14	596	0.0235	[0.0129; 0.0391]	1.3%
Van Cutsem E 2002−A3	7	236	0.0297	[0.0120; 0.0602]	1.2%
Hartung G 2001	0	51	0.0000	[0.0000; 0.0698]	0.9%
Dencausse Y 2001	0	21	0.0000	[0.0000; 0.1611]	0.6%
Recchia F 2001	3	28	0.1071	[0.0227; 0.2823]	0.7%
Riccardi A 2001	2	69	0.0290	[0.0035; 0.1008]	1.0%
Peiffert D 2001	2	80	0.0250	[0.0030; 0.0874]	1.0%
Blum JL 2001	0	74	0.0000	[0.0000; 0.0486]	1.0%
Lin JK 2000	0	40	0.0000	[0.0000; 0.0881]	0.8%
Zambelli A 2000	0	48	0.0000	[0.0000; 0.0740]	0.9%
Balloni L 2000	0	25	0.0000	[0.0000; 0.1372]	0.7%
Susvnjar S 1999	1	19	0.0526	[0.0013; 0.2603]	0.6%
Valero V 1999	2	41	0.0488	[0.0060; 0.1653]	0.8%
Hasbini A 1999	1	44	0.0227	[0.0006; 0.1202]	0.9%
Blum JL 1999	0	162	0.0000	[0.0000; 0.0225]	1.1%
Blajman C 1999	7	85	0.0824	[0.0338; 0.1623]	1.0%
Birkenhake S 1999	0	25	0.0000	[0.0000; 0.1372]	0.7%
Milano G 1999	0	19	0.0000	[0.0000; 0.1765]	0.6%
Mantovani G 1998	1	33	0.0303	[0.0008; 0.1576]	0.8%
Papadimitriou CA 1998	0	24	0.0000	[0.0000; 0.1425]	0.7%
Fetting JH 1998−A1	14	307	0.0456	[0.0252; 0.0753]	1.2%
Fetting JH 1998−A2	26	309	0.0841	[0.0557; 0.1209]	1.2%
Seitz JF 1998	0	24	0.0000	[0.0000; 0.1425]	0.7%
Tominaga T 1998	0	20	0.0000	[0.0000; 0.1684]	0.6%
Wang WS 1998	0	41	0.0000	[0.0000; 0.0860]	0.8%
Taylor SG 1997	0	78	0.0000	[0.0000; 0.0462]	1.0%
Yen CC 1997	5	13	0.3846	[0.1386; 0.6842]	0.5%
Kroning KC 1997	0	27	0.0000	[0.0000; 0.1277]	0.7%
Ychou M 1996	0	28	0.0000	[0.0000; 0.1234]	0.7%
Kok TC 1996	1	29	0.0345	[0.0009; 0.1776]	0.7%
Gebbia V 1996	2	33	0.0606	[0.0074; 0.2023]	0.8%
Hainsworth JD 1996	0	46	0.0000	[0.0000; 0.0771]	0.9%
Colozza M 1996	0	28	0.0000	[0.0000; 0.1234]	0.7%
Mammoliti S 1996	0	24	0.0000	[0.0000; 0.1425]	0.7%
Becouarn YH 1996	1	86	0.0116	[0.0003; 0.0631]	1.0%
Macdonald JS 1995	0	90	0.0000	[0.0000; 0.0402]	1.0%
Leonardi V 1995	0	34	0.0000	[0.0000; 0.1028]	0.8%
Carmo−Pereira J 1995	0	38	0.0000	[0.0000; 0.0925]	0.8%
Haas NB 1995	0	38	0.0000	[0.0000; 0.0925]	0.8%
Klastersky J 1994	5	182	0.0275	[0.0090; 0.0629]	1.2%
Morere JF 1994	3	109	0.0275	[0.0057; 0.0783]	1.1%
Langer CJ 1993	0	40	0.0000	[0.0000; 0.0881]	0.8%
Carmo−Pereira J 1993	0	36	0.0000	[0.0000; 0.0974]	0.8%
Neri B 1993	5	35	0.1429	[0.0481; 0.3026]	0.8%
Jassem J 1993	13	189	0.0688	[0.0371; 0.1147]	1.2%
Kuzel TM 1993	0	22	0.0000	[0.0000; 0.1544]	0.6%
Citron ML 1992	1	86	0.0116	[0.0003; 0.0631]	1.0%
Perit P 1991−A1	7	31	0.2258	[0.0959; 0.4110]	0.8%
Perit P 1991−A2	6	29	0.2069	[0.0799; 0.3972]	0.7%
Zamagni C 1991	3	30	0.1000	[0.0211; 0.2653]	0.7%
Samonigg H 1990	0	21	0.0000	[0.0000; 0.1611]	0.6%
**Random effects model**		**18351**	**0.0233**	**[0.0157; 0.0321]**	**100.0%**

Heterogeneity: I^2 = 84%, τ^2 = 0.0109, p < 0.01

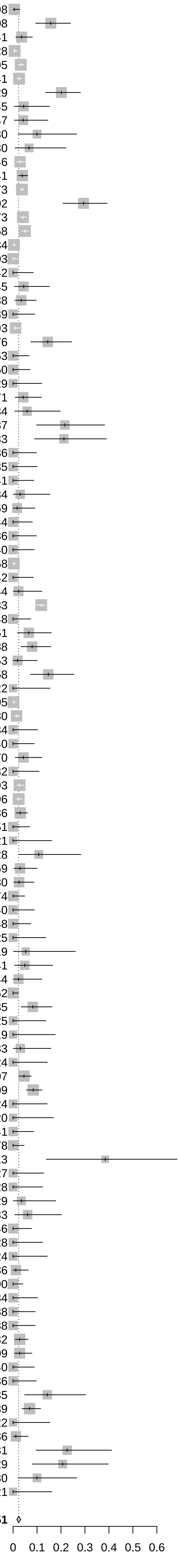

Supplement: Supplementary file 1 [file pharmaceuticals-16-00510-s001.zip › Figure S4 Forest plot of grade 1 or grade 2.pdf]
