# Supplementary material for: Redefining the Incidence and Profile of Fluoropyrimidine-Associated Cardiotoxicity in Cancer Patients: A Systematic Review and Meta-Analysis
Source: Pharmaceuticals (Basel). 2023 Mar 30;16(4):510. doi: 10.3390/ph16040510 (PMC10146083; doi:10.3390/ph16040510)
Supplement: Supplementary file 1 [file pharmaceuticals-16-00510-s001.zip › Figure S9A-F Sensitivity analysis of main outcomes.pdf]

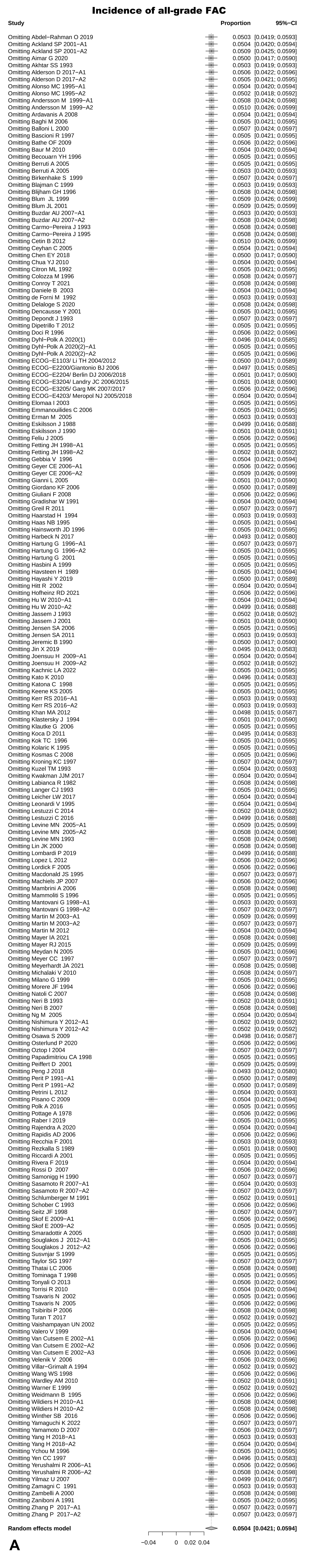

Incidence of all-grade FAC
Study
Proportion
95%−CI
Random effects model
0.0504 [0.0421; 0.0594]
−0.04
0
0.02
0.04
A

Incidence of grade 3 or higher FAC

Study	Proportion	95%−CI
Omitting Mayer IA 2021	0.0152	[0.0110; 0.0198]
Omitting Chakravarthy AB 2020−A1	0.0151	[0.0110; 0.0198]
Omitting Chakravarthy AB 2020−A2	0.0150	[0.0109; 0.0197]
Omitting Dyhl−Polk A 2020(1)	0.0144	[0.0104; 0.0189]
Omitting Osterlund P 2020	0.0152	[0.0111; 0.0199]
Omitting Delaloge S 2020	0.0152	[0.0110; 0.0199]
Omitting Dyhl−Polk A 2020(2)−A1	0.0150	[0.0109; 0.0197]
Omitting Dyhl−Polk A 2020(2)−A2	0.0151	[0.0109; 0.0197]
Omitting Gibson MK 2019	0.0145	[0.0105; 0.0190]
Omitting Jin X 2019	0.0145	[0.0104; 0.0189]
Omitting Rivera F 2019	0.0150	[0.0108; 0.0196]
Omitting Primrose JN 2019	0.0150	[0.0108; 0.0196]
Omitting Chen EY 2018	0.0145	[0.0105; 0.0190]
Omitting Yang H 2018−A1	0.0151	[0.0110; 0.0197]
Omitting Yang H 2018−A2	0.0151	[0.0110; 0.0197]
Omitting Alderson D 2017−A1	0.0152	[0.0110; 0.0198]
Omitting Alderson D 2017−A2	0.0152	[0.0110; 0.0198]
Omitting Kwakman JJM 2017	0.0150	[0.0108; 0.0196]
Omitting Harbeck N 2017	0.0147	[0.0106; 0.0192]
Omitting Kerr RS 2016−A1	0.0149	[0.0107; 0.0195]
Omitting Kerr RS 2016−A2	0.0149	[0.0108; 0.0196]
Omitting Winther SB 2016	0.0149	[0.0108; 0.0196]
Omitting Mayer RJ 2015	0.0152	[0.0111; 0.0199]
Omitting van der Sluis PC 2015−A1	0.0146	[0.0106; 0.0192]
Omitting van der Sluis PC 2015−A2	0.0151	[0.0110; 0.0198]
Omitting Tonyali O 2013	0.0150	[0.0108; 0.0196]
Omitting Okines AFC 2013−A1	0.0151	[0.0109; 0.0197]
Omitting Okines AFC 2013−A2	0.0148	[0.0107; 0.0194]
Omitting Uygun K 2013	0.0152	[0.0111; 0.0199]
Omitting Mackey JR 2013	0.0150	[0.0108; 0.0196]
Omitting Cetin B 2012	0.0153	[0.0111; 0.0199]
Omitting Lang I 2012	0.0153	[0.0111; 0.0199]
Omitting Nishimura Y 2012−A1	0.0146	[0.0106; 0.0191]
Omitting Nishimura Y 2012−A2	0.0148	[0.0107; 0.0194]
Omitting Martin M 2012	0.0150	[0.0108; 0.0196]
Omitting Petrini L 2012	0.0148	[0.0107; 0.0193]
Omitting Dipetrillo T 2012	0.0149	[0.0108; 0.0195]
Omitting Greil R 2011	0.0151	[0.0109; 0.0198]
Omitting Robert NJ 2011−A1	0.0152	[0.0110; 0.0198]
Omitting Robert NJ 2011−A2	0.0151	[0.0109; 0.0197]
Omitting Kato K 2010	0.0142	[0.0103; 0.0186]
Omitting Wildiers H 2010−A1	0.0152	[0.0110; 0.0198]
Omitting Wildiers H 2010−A2	0.0151	[0.0110; 0.0198]
Omitting Masi G 2010	0.0149	[0.0108; 0.0195]
Omitting Michalaki V 2010	0.0151	[0.0110; 0.0197]
Omitting Baur M 2010	0.0150	[0.0109; 0.0197]
Omitting Hu W 2010−A1	0.0151	[0.0110; 0.0198]
Omitting Hu W 2010−A2	0.0150	[0.0108; 0.0196]
Omitting Osawa S 2009	0.0148	[0.0107; 0.0194]
Omitting Pisano C 2009	0.0149	[0.0107; 0.0194]
Omitting Bathe OF 2009	0.0150	[0.0108; 0.0196]
Omitting Skof E 2009−A1	0.0150	[0.0108; 0.0196]
Omitting Skof E 2009−A2	0.0148	[0.0107; 0.0194]
Omitting Giuliani F 2008	0.0150	[0.0108; 0.0196]
Omitting Ardavanis A 2008	0.0150	[0.0108; 0.0196]
Omitting Rothenberg ML 2008−A1	0.0152	[0.0110; 0.0198]
Omitting Rothenberg ML 2008−A2	0.0152	[0.0110; 0.0198]
Omitting Yamamoto D 2007	0.0152	[0.0110; 0.0198]
Omitting Natoli C 2007	0.0151	[0.0110; 0.0198]
Omitting Neri B 2007	0.0151	[0.0110; 0.0198]
Omitting Machiels JP 2007	0.0150	[0.0108; 0.0196]
Omitting Sasamoto R 2007−A1	0.0148	[0.0107; 0.0193]
Omitting Sasamoto R 2007−A2	0.0150	[0.0109; 0.0197]
Omitting ECOG−E3205/ Garg MK 2007/2017	0.0149	[0.0108; 0.0195]
Omitting Emmanouilides C 2006	0.0149	[0.0108; 0.0195]
Omitting Thatai LC 2006	0.0151	[0.0110; 0.0198]
Omitting Jensen SA 2006−A1	0.0150	[0.0109; 0.0197]
Omitting Jensen SA 2006−A2	0.0149	[0.0107; 0.0195]
Omitting Jensen SA 2006−A3	0.0147	[0.0106; 0.0193]
Omitting Jensen SA 2006−A4	0.0151	[0.0109; 0.0196]
Omitting Jensen SA 2006−A5	0.0149	[0.0108; 0.0195]
Omitting Velenik V 2006	0.0150	[0.0109; 0.0196]
Omitting Rapidis AD 2006	0.0150	[0.0108; 0.0196]
Omitting Klautke G 2006	0.0149	[0.0108; 0.0196]
Omitting Giordano KF 2006	0.0144	[0.0105; 0.0189]
Omitting Jatoi A 2006	0.0147	[0.0106; 0.0192]
Omitting Baghi M 2006	0.0149	[0.0108; 0.0195]
Omitting Gianni L 2005	0.0152	[0.0110; 0.0199]
Omitting Bontenbal M 2005	0.0147	[0.0106; 0.0193]
Omitting Lordick F 2005	0.0150	[0.0108; 0.0196]
Omitting Berruti A 2005	0.0150	[0.0109; 0.0196]
Omitting Erman M 2005	0.0152	[0.0110; 0.0198]
Omitting Berruti A 2005	0.0150	[0.0108; 0.0196]
Omitting ECOG−E1103/ Li TH 2004/2012	0.0150	[0.0109; 0.0197]
Omitting Oztop I 2004	0.0151	[0.0110; 0.0197]
Omitting Martin M 2003−A1	0.0153	[0.0112; 0.0199]
Omitting Martin M 2003−A2	0.0152	[0.0110; 0.0198]
Omitting Daniele B 2003	0.0148	[0.0107; 0.0194]
Omitting Elomaa I 2003	0.0149	[0.0107; 0.0195]
Omitting ECOG−E3201 2003/2018−A1	0.0149	[0.0108; 0.0195]
Omitting ECOG−E3201 2003/2018−A2	0.0152	[0.0110; 0.0198]
Omitting ECOG−E3201 2003/2018−A3	0.0150	[0.0109; 0.0196]
Omitting Hitt R 2002	0.0150	[0.0109; 0.0197]
Omitting Vaishampayan UN 2002	0.0150	[0.0108; 0.0196]
Omitting Van Cutsem E 2002−A1	0.0152	[0.0110; 0.0198]
Omitting Van Cutsem E 2002−A2	0.0152	[0.0110; 0.0198]
Omitting Van Cutsem E 2002−A3	0.0153	[0.0111; 0.0199]
Omitting ECOG−E3200/ Giantonio BJ 2001/2007	0.0149	[0.0108; 0.0196]
Omitting Hartung G 2001	0.0149	[0.0108; 0.0195]
Omitting Dencausse Y 2001	0.0150	[0.0108; 0.0195]
Omitting Recchia F 2001	0.0151	[0.0110; 0.0197]
Omitting Riccardi A 2001	0.0150	[0.0109; 0.0197]
Omitting Piccart MJ 2001	0.0153	[0.0111; 0.0199]
Omitting Peiffert D 2001	0.0149	[0.0108; 0.0196]
Omitting Pienkowski T 2001	0.0148	[0.0107; 0.0194]
Omitting Blum JL 2001	0.0152	[0.0110; 0.0198]
Omitting Hoff PM 2001−A1	0.0152	[0.0110; 0.0198]
Omitting Hoff PM 2001−A2	0.0151	[0.0109; 0.0198]
Omitting Lin JK 2000	0.0151	[0.0110; 0.0198]
Omitting Zambelli A 2000	0.0151	[0.0110; 0.0198]
Omitting Balloni L 2000	0.0151	[0.0110; 0.0197]
Omitting Susvnjar S 1999	0.0151	[0.0110; 0.0197]
Omitting Valero V 1999	0.0150	[0.0108; 0.0196]
Omitting Hasbini A 1999	0.0150	[0.0108; 0.0196]
Omitting Blum JL 1999	0.0152	[0.0111; 0.0199]
Omitting Blajman C 1999	0.0152	[0.0110; 0.0198]
Omitting Birkenhake S 1999	0.0151	[0.0110; 0.0197]
Omitting Milano G 1999	0.0150	[0.0108; 0.0196]
Omitting Mantovani G 1998−A1	0.0150	[0.0109; 0.0196]
Omitting Mantovani G 1998−A2	0.0151	[0.0110; 0.0197]
Omitting Papadimitriou CA 1998	0.0149	[0.0108; 0.0195]
Omitting Fetting JH 1998−A1	0.0152	[0.0110; 0.0198]
Omitting Fetting JH 1998−A2	0.0150	[0.0109; 0.0197]
Omitting Seitz JF 1998	0.0151	[0.0110; 0.0197]
Omitting Wang WS 1998	0.0150	[0.0108; 0.0196]
Omitting Taylor SG 1997	0.0150	[0.0109; 0.0197]
Omitting Yen CC 1997	0.0147	[0.0107; 0.0193]
Omitting Kroning KC 1997	0.0151	[0.0110; 0.0197]
Omitting Ychou M 1996	0.0149	[0.0108; 0.0196]
Omitting Kok TC 1996	0.0151	[0.0110; 0.0197]
Omitting Gebbia V 1996	0.0151	[0.0110; 0.0198]
Omitting Hainsworth JD 1996	0.0149	[0.0108; 0.0195]
Omitting Colozza M 1996	0.0151	[0.0110; 0.0197]
Omitting Mammoliti S 1996	0.0149	[0.0108; 0.0195]
Omitting Becouarn YH 1996	0.0149	[0.0108; 0.0195]
Omitting Macdonald JS 1995	0.0151	[0.0109; 0.0197]
Omitting Leonardi V 1995	0.0148	[0.0107; 0.0194]
Omitting Carmo−Pereira J 1995	0.0151	[0.0110; 0.0198]
Omitting Haas NB 1995	0.0149	[0.0107; 0.0194]
Omitting Klastersky J 1994	0.0143	[0.0103; 0.0187]
Omitting Morere JF 1994	0.0152	[0.0111; 0.0198]
Omitting Langer CJ 1993	0.0149	[0.0107; 0.0195]
Omitting Carmo−Pereira J 1993	0.0151	[0.0110; 0.0198]
Omitting Neri B 1993	0.0148	[0.0107; 0.0194]
Omitting Jassem J 1993	0.0148	[0.0107; 0.0195]
Omitting Keefe DL 1993	0.0152	[0.0110; 0.0199]
Omitting Kuzel TM 1993	0.0148	[0.0108; 0.0194]
Omitting Citron ML 1992	0.0149	[0.0108; 0.0195]
Omitting Perit P 1991−A1	0.0151	[0.0110; 0.0198]
Omitting Perit P 1991−A2	0.0151	[0.0110; 0.0197]
Omitting Zamagni C 1991	0.0151	[0.0110; 0.0197]
Omitting Samonigg H 1990	0.0151	[0.0110; 0.0197]
**Random effects model**	**0.0150**	**[0.0109; 0.0196]**

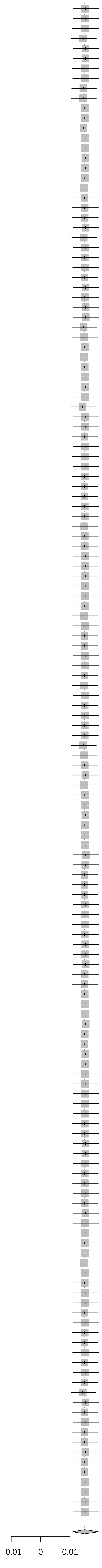

B

Incidence of grade 1-2 FAC

Study	Proportion	95%−CI
Omitting Mayer IA 2021	0.0237	[0.0159; 0.0326]
Omitting Dyhl−Polk A 2020(1)	0.0224	[0.0149; 0.0309]
Omitting Osterlund P 2020	0.0232	[0.0155; 0.0321]
Omitting Delaloge S 2020	0.0237	[0.0159; 0.0326]
Omitting Dyhl−Polk A 2020(2)−A1	0.0232	[0.0155; 0.0322]
Omitting Dyhl−Polk A 2020(2)−A2	0.0233	[0.0156; 0.0323]
Omitting Jin X 2019	0.0221	[0.0148; 0.0305]
Omitting Rivera F 2019	0.0232	[0.0155; 0.0320]
Omitting Chen EY 2018	0.0232	[0.0155; 0.0320]
Omitting Yang H 2018−A1	0.0229	[0.0153; 0.0317]
Omitting Yang H 2018−A2	0.0231	[0.0154; 0.0319]
Omitting Alderson D 2017−A1	0.0233	[0.0155; 0.0322]
Omitting Alderson D 2017−A2	0.0232	[0.0154; 0.0321]
Omitting Kwakman JJM 2017	0.0232	[0.0154; 0.0321]
Omitting Harbeck N 2017	0.0218	[0.0147; 0.0298]
Omitting Kerr RS 2016−A1	0.0231	[0.0154; 0.0320]
Omitting Kerr RS 2016−A2	0.0230	[0.0153; 0.0319]
Omitting Mayer RJ 2015	0.0238	[0.0160; 0.0327]
Omitting Cetin B 2012	0.0239	[0.0161; 0.0328]
Omitting Nishimura Y 2012−A1	0.0236	[0.0159; 0.0325]
Omitting Nishimura Y 2012−A2	0.0232	[0.0155; 0.0320]
Omitting Martin M 2012	0.0232	[0.0155; 0.0321]
Omitting Petrini L 2012	0.0236	[0.0159; 0.0325]
Omitting Greil R 2011	0.0236	[0.0158; 0.0325]
Omitting Kato K 2010	0.0225	[0.0150; 0.0311]
Omitting Wildiers H 2010−A1	0.0237	[0.0159; 0.0326]
Omitting Wildiers H 2010−A2	0.0237	[0.0159; 0.0326]
Omitting Michalaki V 2010	0.0236	[0.0159; 0.0325]
Omitting Baur M 2010	0.0232	[0.0154; 0.0320]
Omitting Hu W 2010−A1	0.0231	[0.0154; 0.0319]
Omitting Hu W 2010−A2	0.0225	[0.0150; 0.0310]
Omitting Osawa S 2009	0.0225	[0.0151; 0.0311]
Omitting Pisano C 2009	0.0236	[0.0159; 0.0325]
Omitting Bathe OF 2009	0.0236	[0.0159; 0.0325]
Omitting Giuliani F 2008	0.0236	[0.0159; 0.0325]
Omitting Ardavanis A 2008	0.0233	[0.0156; 0.0321]
Omitting Yamamoto D 2007	0.0234	[0.0157; 0.0323]
Omitting Natoli C 2007	0.0237	[0.0159; 0.0325]
Omitting Neri B 2007	0.0236	[0.0159; 0.0325]
Omitting Machiels JP 2007	0.0236	[0.0159; 0.0325]
Omitting Jensen SA 2006	0.0238	[0.0160; 0.0327]
Omitting Rapidis AD 2006	0.0236	[0.0159; 0.0325]
Omitting Giordano KF 2006	0.0233	[0.0156; 0.0322]
Omitting Gianni L 2005	0.0225	[0.0150; 0.0311]
Omitting Lordick F 2005	0.0237	[0.0159; 0.0326]
Omitting Berruti A 2005	0.0230	[0.0153; 0.0318]
Omitting Erman M 2005	0.0229	[0.0152; 0.0317]
Omitting Berruti A 2005	0.0234	[0.0156; 0.0323]
Omitting ECOG−E1103/ Li TH 2004/2012	0.0225	[0.0150; 0.0312]
Omitting Oztop I 2004	0.0236	[0.0159; 0.0325]
Omitting Martin M 2003−A1	0.0238	[0.0161; 0.0327]
Omitting Martin M 2003−A2	0.0235	[0.0157; 0.0324]
Omitting Daniele B 2003	0.0236	[0.0159; 0.0325]
Omitting Elomaa I 2003	0.0236	[0.0159; 0.0325]
Omitting Hitt R 2002	0.0232	[0.0154; 0.0320]
Omitting Vaishampayan UN 2002	0.0236	[0.0159; 0.0325]
Omitting Van Cutsem E 2002−A1	0.0233	[0.0156; 0.0322]
Omitting Van Cutsem E 2002−A2	0.0234	[0.0156; 0.0323]
Omitting Van Cutsem E 2002−A3	0.0233	[0.0155; 0.0322]
Omitting Hartung G 2001	0.0237	[0.0159; 0.0326]
Omitting Dencausse Y 2001	0.0236	[0.0159; 0.0325]
Omitting Recchia F 2001	0.0229	[0.0153; 0.0317]
Omitting Riccardi A 2001	0.0233	[0.0155; 0.0321]
Omitting Peiffert D 2001	0.0233	[0.0156; 0.0322]
Omitting Blum JL 2001	0.0237	[0.0160; 0.0326]
Omitting Lin JK 2000	0.0236	[0.0159; 0.0325]
Omitting Zambelli A 2000	0.0237	[0.0159; 0.0326]
Omitting Balloni L 2000	0.0236	[0.0159; 0.0325]
Omitting Susvnjar S 1999	0.0232	[0.0156; 0.0321]
Omitting Valero V 1999	0.0231	[0.0155; 0.0320]
Omitting Hasbini A 1999	0.0233	[0.0156; 0.0322]
Omitting Blum JL 1999	0.0238	[0.0161; 0.0327]
Omitting Blajman C 1999	0.0229	[0.0152; 0.0316]
Omitting Birkenhake S 1999	0.0236	[0.0159; 0.0325]
Omitting Milano G 1999	0.0236	[0.0159; 0.0324]
Omitting Mantovani G 1998	0.0233	[0.0156; 0.0321]
Omitting Papadimitriou CA 1998	0.0236	[0.0159; 0.0325]
Omitting Fetting JH 1998−A1	0.0231	[0.0154; 0.0320]
Omitting Fetting JH 1998−A2	0.0228	[0.0151; 0.0315]
Omitting Seitz JF 1998	0.0236	[0.0159; 0.0325]
Omitting Tominaga T 1998	0.0236	[0.0159; 0.0324]
Omitting Wang WS 1998	0.0236	[0.0159; 0.0325]
Omitting Taylor SG 1997	0.0237	[0.0160; 0.0326]
Omitting Yen CC 1997	0.0227	[0.0153; 0.0312]
Omitting Kroning KC 1997	0.0236	[0.0159; 0.0325]
Omitting Ychou M 1996	0.0236	[0.0159; 0.0325]
Omitting Kok TC 1996	0.0233	[0.0156; 0.0321]
Omitting Gebbia V 1996	0.0231	[0.0154; 0.0319]
Omitting Hainsworth JD 1996	0.0237	[0.0159; 0.0325]
Omitting Colozza M 1996	0.0236	[0.0159; 0.0325]
Omitting Mammoliti S 1996	0.0236	[0.0159; 0.0325]
Omitting Becouarn YH 1996	0.0235	[0.0157; 0.0324]
Omitting Macdonald JS 1995	0.0238	[0.0160; 0.0327]
Omitting Leonardi V 1995	0.0236	[0.0159; 0.0325]
Omitting Carmo−Pereira J 1995	0.0236	[0.0159; 0.0325]
Omitting Haas NB 1995	0.0236	[0.0159; 0.0325]
Omitting Klastersky J 1994	0.0233	[0.0156; 0.0322]
Omitting Morere JF 1994	0.0233	[0.0156; 0.0322]
Omitting Langer CJ 1993	0.0236	[0.0159; 0.0325]
Omitting Carmo−Pereira J 1993	0.0236	[0.0159; 0.0325]
Omitting Neri B 1993	0.0227	[0.0152; 0.0314]
Omitting Jassem J 1993	0.0229	[0.0152; 0.0317]
Omitting Kuzel TM 1993	0.0236	[0.0159; 0.0325]
Omitting Citron ML 1992	0.0235	[0.0157; 0.0324]
Omitting Perit P 1991−A1	0.0225	[0.0151; 0.0310]
Omitting Perit P 1991−A2	0.0226	[0.0151; 0.0312]
Omitting Zamagni C 1991	0.0229	[0.0153; 0.0317]
Omitting Samonigg H 1990	0.0236	[0.0159; 0.0325]
**Random effects model**	**0.0233**	**[0.0157; 0.0321]**

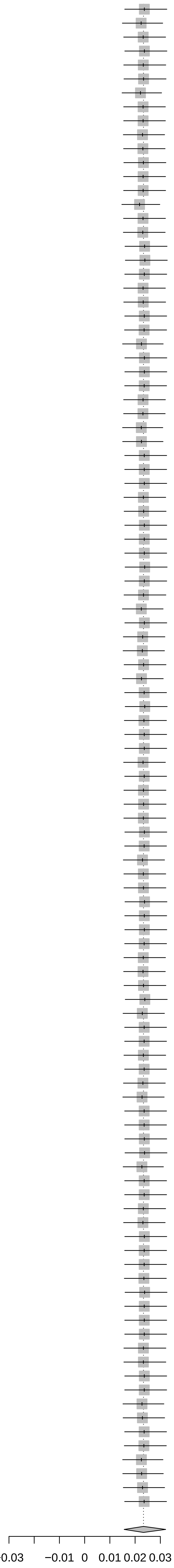

C

Incidence of cardiac ischemia

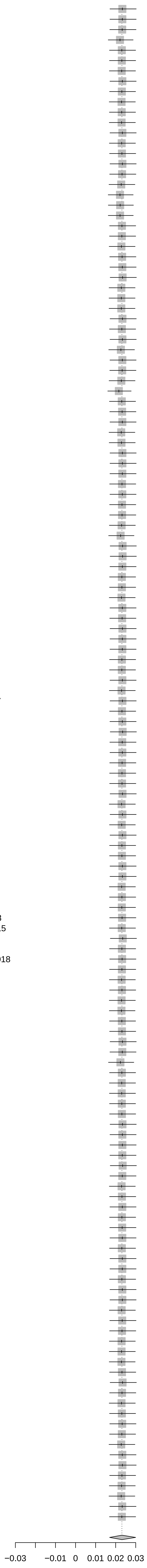

Study	Proportion	95%−CI
Omitting Mayer IA 2021	0.0233	[0.0172; 0.0302]
Omitting Chakravarthy AB 2020−A1	0.0233	[0.0171; 0.0302]
Omitting Chakravarthy AB 2020−A2	0.0232	[0.0171; 0.0302]
Omitting Dyhl−Polk A 2020(1)	0.0222	[0.0163; 0.0287]
Omitting Aimar G 2020	0.0231	[0.0169; 0.0300]
Omitting Osterlund P 2020	0.0231	[0.0169; 0.0300]
Omitting Rajendra A 2020	0.0230	[0.0169; 0.0299]
Omitting Delaloge S 2020	0.0233	[0.0171; 0.0302]
Omitting Grierson P 2020	0.0231	[0.0170; 0.0299]
Omitting Dyhl−Polk A 2020(2)−A1	0.0229	[0.0168; 0.0298]
Omitting Dyhl−Polk A 2020(2)−A2	0.0230	[0.0168; 0.0299]
Omitting Raber I 2019	0.0230	[0.0168; 0.0298]
Omitting Shanmuganathan JWD 2019	0.0233	[0.0172; 0.0303]
Omitting Lombardi P 2019	0.0230	[0.0168; 0.0299]
Omitting Jin X 2019	0.0231	[0.0169; 0.0300]
Omitting Primrose JN 2019	0.0233	[0.0172; 0.0303]
Omitting Abdel−Rahman O 2019	0.0232	[0.0170; 0.0301]
Omitting Hayashi Y 2019	0.0228	[0.0167; 0.0296]
Omitting Peng J 2018	0.0222	[0.0163; 0.0287]
Omitting Peng J 2018−A1	0.0222	[0.0163; 0.0289]
Omitting Peng J 2018−A2	0.0222	[0.0163; 0.0288]
Omitting Chen EY 2018	0.0231	[0.0170; 0.0300]
Omitting Kwakman JJM 2017	0.0231	[0.0169; 0.0300]
Omitting Turan T 2017	0.0229	[0.0168; 0.0297]
Omitting Leicher LW 2017	0.0232	[0.0170; 0.0301]
Omitting Zhang P 2017−A1	0.0233	[0.0171; 0.0302]
Omitting Zhang P 2017−A2	0.0234	[0.0173; 0.0303]
Omitting Kerr RS 2016−A1	0.0228	[0.0167; 0.0297]
Omitting Kerr RS 2016−A2	0.0228	[0.0167; 0.0297]
Omitting Lestuzzi C 2016	0.0228	[0.0167; 0.0297]
Omitting Winther SB 2016	0.0234	[0.0172; 0.0303]
Omitting Polk A 2016	0.0231	[0.0169; 0.0300]
Omitting Mayer RJ 2015	0.0234	[0.0172; 0.0303]
Omitting Lestuzzi C 2014	0.0226	[0.0165; 0.0294]
Omitting Tonyali O 2013	0.0233	[0.0171; 0.0302]
Omitting Okines AFC 2013−A1	0.0232	[0.0171; 0.0301]
Omitting Okines AFC 2013−A2	0.0228	[0.0167; 0.0297]
Omitting Cen P 2012	0.0216	[0.0161; 0.0277]
Omitting Khan MA 2012	0.0230	[0.0169; 0.0299]
Omitting Martin M 2012	0.0232	[0.0170; 0.0301]
Omitting Petrini L 2012	0.0233	[0.0172; 0.0302]
Omitting Koca D 2011	0.0228	[0.0167; 0.0296]
Omitting Jensen SA 2011	0.0229	[0.0168; 0.0298]
Omitting Wildiers H 2010−A1	0.0234	[0.0172; 0.0302]
Omitting Wildiers H 2010−A2	0.0233	[0.0172; 0.0302]
Omitting Torrisi R 2010	0.0233	[0.0171; 0.0302]
Omitting Masi G 2010	0.0232	[0.0170; 0.0300]
Omitting Michalaki V 2010	0.0233	[0.0171; 0.0302]
Omitting Chua YJ 2010	0.0232	[0.0170; 0.0301]
Omitting Baur M 2010	0.0232	[0.0170; 0.0301]
Omitting Hu W 2010−A1	0.0230	[0.0168; 0.0298]
Omitting Hu W 2010−A2	0.0225	[0.0165; 0.0292]
Omitting Joensuu H 2009−A1	0.0234	[0.0173; 0.0303]
Omitting Joensuu H 2009−A2	0.0234	[0.0173; 0.0303]
Omitting Pisano C 2009	0.0233	[0.0171; 0.0302]
Omitting Bathe OF 2009	0.0231	[0.0169; 0.0300]
Omitting Skof E 2009−A1	0.0231	[0.0170; 0.0300]
Omitting Skof E 2009−A2	0.0229	[0.0168; 0.0298]
Omitting Ardavanis A 2008	0.0233	[0.0171; 0.0302]
Omitting Kosmas C 2008	0.0232	[0.0170; 0.0301]
Omitting Yamamoto D 2007	0.0234	[0.0172; 0.0303]
Omitting Natoli C 2007	0.0233	[0.0172; 0.0302]
Omitting Neri B 2007	0.0233	[0.0171; 0.0302]
Omitting Machiels JP 2007	0.0231	[0.0170; 0.0300]
Omitting Buzdar AU 2007−A1	0.0231	[0.0169; 0.0299]
Omitting Buzdar AU 2007−A2	0.0233	[0.0172; 0.0302]
Omitting Yilmaz U 2007	0.0230	[0.0168; 0.0298]
Omitting ECOG−E3205/ Garg MK 2007/2017	0.0234	[0.0172; 0.0303]
Omitting Emmanouilides C 2006	0.0231	[0.0170; 0.0300]
Omitting Geyer CE 2006−A1	0.0233	[0.0171; 0.0302]
Omitting Geyer CE 2006−A2	0.0235	[0.0173; 0.0304]
Omitting Mambrini A 2006	0.0233	[0.0171; 0.0302]
Omitting Thatai LC 2006	0.0233	[0.0172; 0.0302]
Omitting Koopman M 2006−A1	0.0232	[0.0170; 0.0301]
Omitting Koopman M 2006−A2	0.0231	[0.0170; 0.0301]
Omitting Jensen SA 2006−A1	0.0232	[0.0170; 0.0301]
Omitting Jensen SA 2006−A2	0.0234	[0.0172; 0.0303]
Omitting Jensen SA 2006−A3	0.0229	[0.0168; 0.0298]
Omitting Jensen SA 2006−A4	0.0234	[0.0172; 0.0302]
Omitting Jensen SA 2006−A5	0.0230	[0.0169; 0.0298]
Omitting Velenik V 2006	0.0234	[0.0172; 0.0303]
Omitting Rapidis AD 2006	0.0231	[0.0170; 0.0300]
Omitting Yerushalmi R 2006−A1	0.0231	[0.0170; 0.0300]
Omitting Yerushalmi R 2006−A2	0.0233	[0.0172; 0.0302]
Omitting Tsibiribi P 2006	0.0233	[0.0172; 0.0303]
Omitting Giordano KF 2006	0.0230	[0.0169; 0.0299]
Omitting ECOG−E2200/Giantonio BJ 2006	0.0231	[0.0169; 0.0300]
Omitting Baghi M 2006	0.0231	[0.0169; 0.0299]
Omitting ECOG−E2204/ Berlin DJ 2006/2018	0.0232	[0.0170; 0.0301]
Omitting ECOG−E3204/ Landry JC 2006/2015	0.0230	[0.0169; 0.0299]
Omitting Gianni L 2005	0.0235	[0.0173; 0.0304]
Omitting Meydan N 2005	0.0231	[0.0169; 0.0300]
Omitting ECOG−E4203/ Meropol NJ 2005/2018	0.0232	[0.0170; 0.0301]
Omitting Tsavaris N 2005	0.0231	[0.0169; 0.0300]
Omitting Ceyhan C 2005	0.0230	[0.0168; 0.0298]
Omitting Lordick F 2005	0.0231	[0.0170; 0.0300]
Omitting Ng M 2005	0.0230	[0.0168; 0.0298]
Omitting Smaradottir A 2005	0.0229	[0.0168; 0.0297]
Omitting Keene KS 2005	0.0231	[0.0169; 0.0299]
Omitting Feliu J 2005	0.0231	[0.0170; 0.0300]
Omitting ECOG−E1103/ Li TH 2004/2012	0.0234	[0.0172; 0.0303]
Omitting Oztop I 2004	0.0233	[0.0171; 0.0302]
Omitting Wacker A 2003	0.0224	[0.0164; 0.0290]
Omitting Vaishampayan UN 2002	0.0231	[0.0169; 0.0300]
Omitting Tsavaris N 2002	0.0230	[0.0169; 0.0299]
Omitting Hartung G 2001	0.0230	[0.0169; 0.0299]
Omitting Dencausse Y 2001	0.0231	[0.0169; 0.0299]
Omitting Peiffert D 2001	0.0231	[0.0169; 0.0300]
Omitting Blum JL 2001	0.0234	[0.0172; 0.0303]
Omitting Lin JK 2000	0.0233	[0.0172; 0.0302]
Omitting Zambelli A 2000	0.0233	[0.0172; 0.0302]
Omitting Balloni L 2000	0.0233	[0.0171; 0.0302]
Omitting Blum JL 1999	0.0235	[0.0173; 0.0304]
Omitting Birkenhake S 1999	0.0233	[0.0171; 0.0302]
Omitting Warner E 1999	0.0230	[0.0168; 0.0298]
Omitting Katona C 1998	0.0231	[0.0170; 0.0300]
Omitting Seitz JF 1998	0.0233	[0.0171; 0.0302]
Omitting Wang WS 1998	0.0231	[0.0170; 0.0300]
Omitting Meyer CC 1997	0.0232	[0.0170; 0.0301]
Omitting Kroning KC 1997	0.0233	[0.0171; 0.0302]
Omitting Kok TC 1996	0.0231	[0.0169; 0.0299]
Omitting Gebbia V 1996	0.0233	[0.0171; 0.0302]
Omitting Colozza M 1996	0.0233	[0.0171; 0.0302]
Omitting Weidmann B 1995	0.0231	[0.0169; 0.0300]
Omitting Leonardi V 1995	0.0233	[0.0171; 0.0302]
Omitting Carmo−Pereira J 1995	0.0233	[0.0172; 0.0302]
Omitting Haas NB 1995	0.0230	[0.0168; 0.0298]
Omitting Klastersky J 1994	0.0232	[0.0171; 0.0302]
Omitting Morere JF 1994	0.0232	[0.0170; 0.0301]
Omitting Haarstad H 1994	0.0230	[0.0168; 0.0298]
Omitting Akhtar SS 1993	0.0229	[0.0168; 0.0298]
Omitting Langer CJ 1993	0.0229	[0.0168; 0.0297]
Omitting Jassem J 1993	0.0231	[0.0170; 0.0300]
Omitting Keefe DL 1993	0.0234	[0.0172; 0.0303]
Omitting Schober C 1993	0.0232	[0.0170; 0.0301]
Omitting de Forni M 1992	0.0229	[0.0168; 0.0298]
Omitting Zaniboni A 1991	0.0231	[0.0169; 0.0300]
Omitting Gradishar W 1991	0.0232	[0.0171; 0.0302]
Omitting Zamagni C 1991	0.0231	[0.0169; 0.0300]
Omitting Jeremic B 1990	0.0228	[0.0167; 0.0296]
Omitting Samonigg H 1990	0.0233	[0.0171; 0.0302]
Omitting Eskilsson J 1990	0.0234	[0.0172; 0.0303]
Omitting Havsteen H 1989	0.0231	[0.0170; 0.0300]
Omitting Rezkalla S 1989	0.0230	[0.0168; 0.0298]
Omitting Eskilsson J 1988	0.0228	[0.0167; 0.0296]
Omitting Labianca R 1982	0.0232	[0.0171; 0.0302]
Omitting Pottage A 1978	0.0231	[0.0169; 0.0300]
**Random effects model**	**0.0231**	**[0.0170; 0.0300]**

D

Incidence of arrhythmia

Study	Proportion	95%−CI
Omitting Blum JL 1999	0.0173	[0.0111; 0.0247]
Omitting Abdel−Rahman O 2019	0.0166	[0.0106; 0.0239]
Omitting Aimar G 2020	0.0169	[0.0108; 0.0243]
Omitting Akhtar SS 1993	0.0167	[0.0107; 0.0241]
Omitting Andersson M 1999−A2	0.0173	[0.0111; 0.0247]
Omitting Balloni L 2000	0.0171	[0.0110; 0.0245]
Omitting Bathe OF 2009	0.0168	[0.0107; 0.0242]
Omitting Birkenhake S 1999	0.0171	[0.0110; 0.0245]
Omitting Blum JL 2001	0.0172	[0.0111; 0.0247]
Omitting Buzdar AU 2007−A2	0.0172	[0.0111; 0.0246]
Omitting Carmo−Pereira J 1993	0.0172	[0.0110; 0.0246]
Omitting Carmo−Pereira J 1995	0.0172	[0.0110; 0.0246]
Omitting Cetin B 2012	0.0173	[0.0111; 0.0247]
Omitting Chakravarthy AB 2020−A1	0.0173	[0.0111; 0.0247]
Omitting Chakravarthy AB 2020−A2	0.0173	[0.0111; 0.0247]
Omitting Chen EY 2018	0.0165	[0.0105; 0.0238]
Omitting Chua YJ 2010	0.0168	[0.0108; 0.0243]
Omitting Colozza M 1996	0.0171	[0.0110; 0.0246]
Omitting de Forni M 1992	0.0171	[0.0110; 0.0246]
Omitting Dipetrillo T 2012	0.0167	[0.0107; 0.0241]
Omitting Dyhl−Polk A 2020(1)	0.0167	[0.0106; 0.0241]
Omitting ECOG−E2200/Giantonio BJ 2006	0.0168	[0.0107; 0.0242]
Omitting ECOG−E2204/ Berlin DJ 2006/2018	0.0169	[0.0108; 0.0243]
Omitting ECOG−E3200/ Giantonio BJ 2001/2007	0.0170	[0.0109; 0.0245]
Omitting ECOG−E3201 2003/2018−A1	0.0169	[0.0108; 0.0243]
Omitting ECOG−E3204/ Landry JC 2006/2015	0.0172	[0.0111; 0.0246]
Omitting ECOG−E3205/ Garg MK 2007/2017	0.0172	[0.0111; 0.0247]
Omitting ECOG−E4203/ Meropol NJ 2005/2018	0.0167	[0.0106; 0.0241]
Omitting Emmanouilides C 2006	0.0169	[0.0108; 0.0243]
Omitting Eskilsson J 1988	0.0166	[0.0106; 0.0239]
Omitting Eskilsson J 1990	0.0164	[0.0105; 0.0236]
Omitting Gebbia V 1996	0.0166	[0.0106; 0.0240]
Omitting Gibson MK 2019	0.0166	[0.0106; 0.0239]
Omitting Giordano KF 2006	0.0166	[0.0106; 0.0239]
Omitting Hartung G 1996−A1	0.0171	[0.0110; 0.0245]
Omitting Hayashi Y 2019	0.0169	[0.0108; 0.0243]
Omitting Jassem J 1993	0.0165	[0.0105; 0.0238]
Omitting Jensen SA 2006−A2	0.0169	[0.0108; 0.0244]
Omitting Jensen SA 2006−A3	0.0171	[0.0110; 0.0246]
Omitting Jensen SA 2006−A5	0.0167	[0.0106; 0.0240]
Omitting Jeremic B 1990	0.0165	[0.0105; 0.0238]
Omitting Jin X 2019	0.0164	[0.0105; 0.0237]
Omitting Keefe DL 1993	0.0171	[0.0110; 0.0246]
Omitting Kerr RS 2016−A1	0.0170	[0.0109; 0.0245]
Omitting Kerr RS 2016−A2	0.0171	[0.0110; 0.0246]
Omitting Khan MA 2012	0.0160	[0.0103; 0.0229]
Omitting Koca D 2011	0.0158	[0.0102; 0.0226]
Omitting Kosmas C 2008	0.0168	[0.0107; 0.0242]
Omitting Kroning KC 1997	0.0171	[0.0110; 0.0246]
Omitting Kuzel TM 1993	0.0167	[0.0107; 0.0241]
Omitting Kwakman JJM 2017	0.0169	[0.0108; 0.0243]
Omitting Kwakman JJM 2017−A1	0.0170	[0.0108; 0.0244]
Omitting Kwakman JJM 2017−A2	0.0169	[0.0108; 0.0244]
Omitting Kwakman JJM 2017−A3	0.0167	[0.0106; 0.0240]
Omitting Kwakman JJM 2017−A4	0.0168	[0.0107; 0.0243]
Omitting Kwakman JJM 2017−A5	0.0171	[0.0110; 0.0245]
Omitting Kwakman JJM 2017−A7	0.0170	[0.0109; 0.0245]
Omitting Labianca R 1982	0.0172	[0.0110; 0.0246]
Omitting Lang I 2012	0.0173	[0.0111; 0.0247]
Omitting Leicher LW 2017	0.0167	[0.0107; 0.0241]
Omitting Lestuzzi C 2016	0.0165	[0.0105; 0.0239]
Omitting Levine MN 1993	0.0171	[0.0110; 0.0246]
Omitting Lin JK 2000	0.0172	[0.0110; 0.0246]
Omitting Lombardi P 2019	0.0168	[0.0108; 0.0243]
Omitting Mantovani G 1998−A1	0.0167	[0.0107; 0.0240]
Omitting Mantovani G 1998−A2	0.0171	[0.0110; 0.0245]
Omitting Meydan N 2005	0.0169	[0.0108; 0.0244]
Omitting Meyer CC 1997	0.0170	[0.0109; 0.0245]
Omitting Michalaki V 2010	0.0171	[0.0110; 0.0246]
Omitting Morere JF 1994	0.0170	[0.0109; 0.0244]
Omitting Natoli C 2007	0.0172	[0.0111; 0.0246]
Omitting Neri B 2007	0.0172	[0.0110; 0.0246]
Omitting Ng M 2005	0.0169	[0.0108; 0.0244]
Omitting Osterlund P 2020	0.0165	[0.0105; 0.0238]
Omitting Oztop I 2004	0.0171	[0.0110; 0.0245]
Omitting Peng J 2018	0.0158	[0.0102; 0.0226]
Omitting Petrini L 2012	0.0167	[0.0106; 0.0240]
Omitting Polk A 2016	0.0169	[0.0108; 0.0244]
Omitting Rajendra A 2020	0.0167	[0.0107; 0.0242]
Omitting Samonigg H 1990	0.0171	[0.0110; 0.0245]
Omitting Schober C 1993	0.0169	[0.0108; 0.0244]
Omitting Seitz JF 1998	0.0171	[0.0110; 0.0245]
Omitting Smaradottir A 2005	0.0167	[0.0106; 0.0240]
Omitting Thatai LC 2006	0.0172	[0.0110; 0.0246]
Omitting Tsavaris N 2002	0.0165	[0.0105; 0.0238]
Omitting Van Cutsem E 2002−A1	0.0172	[0.0110; 0.0246]
Omitting Van Cutsem E 2002−A2	0.0172	[0.0110; 0.0246]
Omitting Weidmann B 1995	0.0168	[0.0107; 0.0242]
Omitting Wildiers H 2010−A1	0.0172	[0.0111; 0.0246]
Omitting Wildiers H 2010−A2	0.0172	[0.0111; 0.0246]
Omitting Yerushalmi R 2006−A2	0.0172	[0.0111; 0.0246]
Omitting Yilmaz U 2007	0.0162	[0.0104; 0.0233]
Omitting Zambelli A 2000	0.0172	[0.0111; 0.0246]
Omitting Zhang P 2017−A1	0.0168	[0.0107; 0.0242]
Omitting Dyhl−Polk A 2020(2)−A1	0.0172	[0.0111; 0.0247]
Omitting Tsavaris N 2005	0.0170	[0.0109; 0.0245]
Omitting Peng J 2018−A1	0.0160	[0.0103; 0.0230]
Omitting Peng J 2018−A2	0.0157	[0.0102; 0.0225]
**Random effects model**	**0.0169**	**[0.0108; 0.0242]**

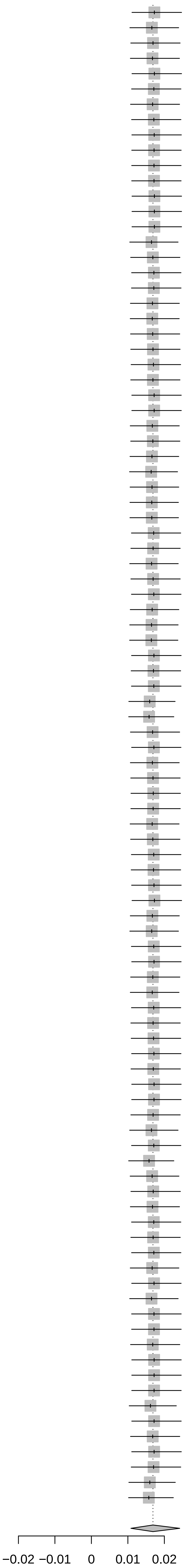

E

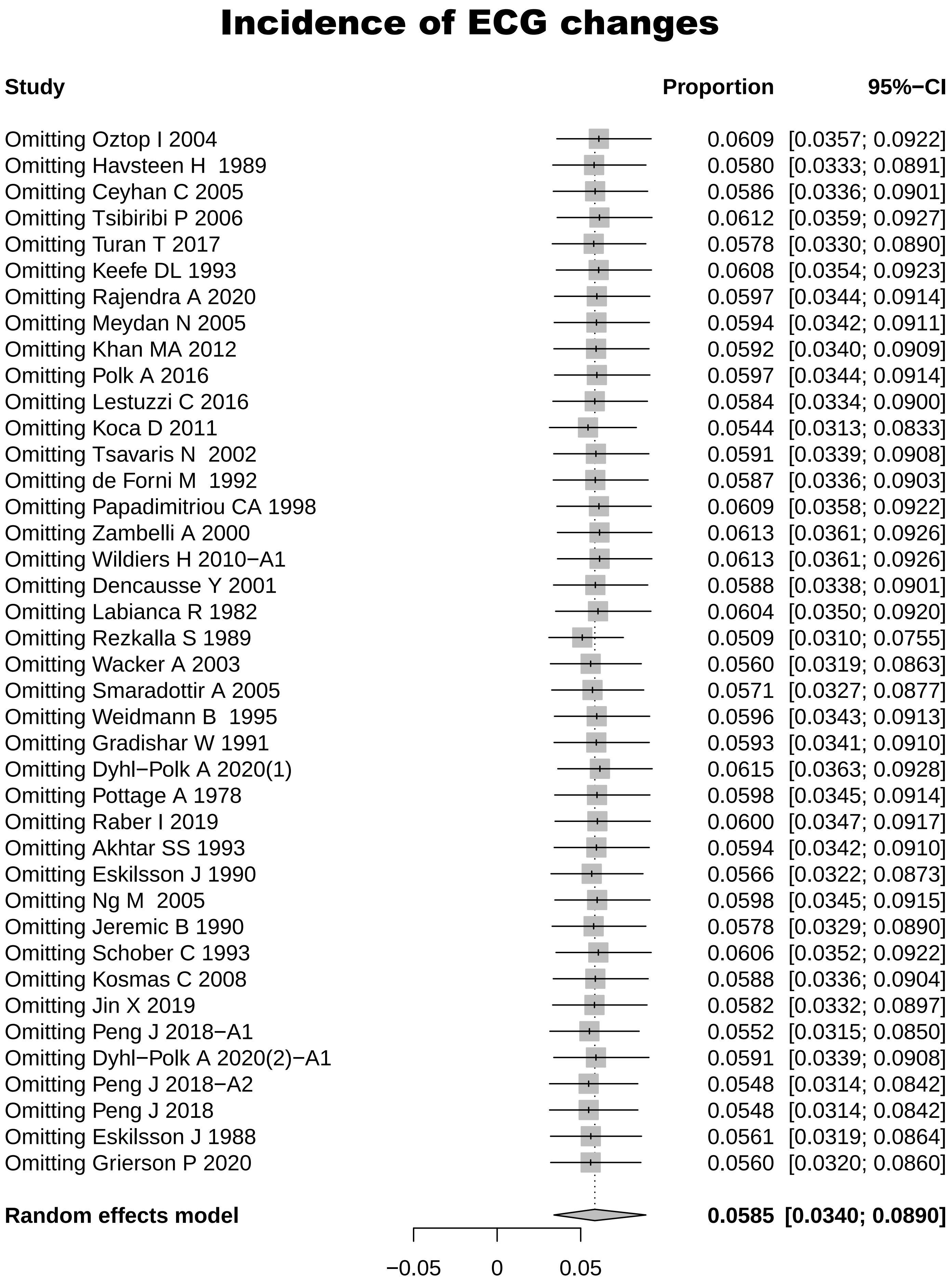
Incidence of ECG changes
Study
Proportion
95%−CI
Omitting Oztop I 2004 0.0609 [0.0357; 0.0922]
Omitting Havsteen H 1989 0.0580 [0.0333; 0.0891]
Omitting Ceyhan C 2005 0.0586 [0.0336; 0.0901]
Omitting Tsibiribi P 2006 0.0612 [0.0359; 0.0927]
Omitting Turan T 2017 0.0578 [0.0330; 0.0890]
Omitting Keefe DL 1993 0.0608 [0.0354; 0.0923]
Omitting Rajendra A 2020 0.0597 [0.0344; 0.0914]
Omitting Meydan N 2005 0.0594 [0.0342; 0.0911]
Omitting Khan MA 2012 0.0592 [0.0340; 0.0909]
Omitting Polk A 2016 0.0597 [0.0344; 0.0914]
Omitting Lestuzzi C 2016 0.0584 [0.0334; 0.0900]
Omitting Koca D 2011 0.0544 [0.0313; 0.0833]
Omitting Tsavaris N 2002 0.0591 [0.0339; 0.0908]
Omitting de Forni M 1992 0.0587 [0.0336; 0.0903]
Omitting Papadimitriou CA 1998 0.0609 [0.0358; 0.0922]
Omitting Zambelli A 2000 0.0613 [0.0361; 0.0926]
Omitting Wildiers H 2010−A1 0.0613 [0.0361; 0.0926]
Omitting Dencausse Y 2001 0.0588 [0.0338; 0.0901]
Omitting Labianca R 1982 0.0604 [0.0350; 0.0920]
Omitting Rezkalla S 1989 0.0509 [0.0310; 0.0755]
Omitting Wacker A 2003 0.0560 [0.0319; 0.0863]
Omitting Smaradottir A 2005 0.0571 [0.0327; 0.0877]
Omitting Weidmann B 1995 0.0596 [0.0343; 0.0913]
Omitting Gradishar W 1991 0.0593 [0.0341; 0.0910]
Omitting Dyhl−Polk A 2020(1) 0.0615 [0.0363; 0.0928]
Omitting Pottage A 1978 0.0598 [0.0345; 0.0914]
Omitting Raber I 2019 0.0600 [0.0347; 0.0917]
Omitting Akhtar SS 1993 0.0594 [0.0342; 0.0910]
Omitting Eskilsson J 1990 0.0566 [0.0322; 0.0873]
Omitting Ng M 2005 0.0598 [0.0345; 0.0915]
Omitting Jeremic B 1990 0.0578 [0.0329; 0.0890]
Omitting Schober C 1993 0.0606 [0.0352; 0.0922]
Omitting Kosmas C 2008 0.0588 [0.0336; 0.0904]
Omitting Jin X 2019 0.0582 [0.0332; 0.0897]
Omitting Peng J 2018−A1 0.0552 [0.0315; 0.0850]
Omitting Dyhl−Polk A 2020(2)−A1 0.0591 [0.0339; 0.0908]
Omitting Peng J 2018−A2 0.0548 [0.0314; 0.0842]
Omitting Peng J 2018 0.0548 [0.0314; 0.0842]
Omitting Eskilsson J 1988 0.0561 [0.0319; 0.0864]
Omitting Grierson P 2020 0.0560 [0.0320; 0.0860]
Random effects model 0.0585 [0.0340; 0.0890]
−0.05
0
0.05

F
