# Supplementary material for: Redefining the Incidence and Profile of Fluoropyrimidine-Associated Cardiotoxicity in Cancer Patients: A Systematic Review and Meta-Analysis
Source: Pharmaceuticals (Basel). 2023 Mar 30;16(4):510. doi: 10.3390/ph16040510 (PMC10146083; doi:10.3390/ph16040510)
Supplement: Supplementary file 1 [file pharmaceuticals-16-00510-s001.zip › Supplementary Figure legends.pdf]

Supplementary Figure Legends

Supplementary Fig. S1

Number of articles published by year. Articles on the anthracyclines associated cardiotoxicity have increased dramatically in the past two decades, and the cardiotoxicity of immune checkpoint inhibitors (ICIs) has also attracted much attention in the nearly 5 years. However, fluoropyrimidine associated cardiotoxicity (FAC) has not received universal attention, and there is no obvious tendency of increase in the number of published articles. Literature retrieval scheme in Web of Science database as following: 1) fluoropyrimidines: "AB=(fluoropyridine or fluoropyrimidine or 5-fluorouracil or 5-FU or fluorouracil or capecitabine or Xeloda or tegafur or S-1 or UFT or TAS-102 or lonsurf or FTD TPI or trifluridine or tipiracil)" AND "AB=(heart or cardiac or cardiovascular or cardiotoxicity)"; 2) anthracyclines: "AB=(anthracycline or doxorubicin or epirubicin or daunorubicin or arubicin or idarubicin or valrubicin or mitoxantrone or aclarubicin)" AND "AB=(heart or cardiac or cardiovascular or cardiotoxicity)"; 3) ICIs: "AB=(PD-1or PD-L1 or CTLA-4 or immune checkpoint)" AND "AB=(heart or cardiac or cardiovascular or cardiotoxicity)".

Supplementary Fig. S2

Forest plot of the pooled incidence of all-grade FAC using a random-effects model (the pooled incidence=5.04%, 95%CI 4.21%-5.94%).

Supplementary Fig. S3

Forest plot for the analysis of incidence of the grade 3 or higher cardiac AEs using a random-effects model (the pooled incidence=1.5%, 95%CI 1.09%-1.96%).

Supplementary Fig. S4

Forest plot for the analysis of incidence of grade 1 or grade 2 cardiac AEs using a random-effects model (the pooled incidence=2.33%, 95%CI 1.57%-3.21%).

Supplementary Fig. S5

Forest plot for the subgroup analysis by 5-FU administration pattern (24-h continuous infusion, bolus infusion for non-consecutive days, bolus infusion followed by continuous infusion, bolus infusion for 3-5 consecutive days, continuous infusion d1,8 or d1, and continuous infusion for 3-5 consecutive days) using a random-effects model. A significant difference was detected between different 5-FU administration patterns ($\chi 2=12.29$, $p=0.03$).

Supplementary Fig. S6

Forest plot for the subgroup analysis by cumulative 5-FU dose (≤1000, 1000-2000, 2000-3000, and >3000) using a random-effects model. A significant difference was detected between different cumulative 5-FU dosage ($\chi 2=8.41$, $p=0.04$).

Supplementary Fig. S7

The bubble plots showed the results of univariable meta-regression of continuous data (female proportion, 5-FU dosage schedule, publication year, and median age). A. significant negative relationship between female proportion and the incidence of cardiotoxicities ($Q=8.59$, $p<0.01$); B. significant positive

relationship between 5-FU dosage schedule and the incidence of cardiotoxicities ($Q=9.57$, $p<0.01$); C. marginally significant positive relationship between publication year and the incidence of cardiotoxicities ($Q=3.69$, $p=0.058$); D. marginally significant positive relationship between median age and the incidence of cardiotoxicities ($Q=3.33$, $p=0.071$).

Supplementary Fig. S8

Egger's funnel plot for publication bias test of the main outcomes, no evidence of significant publication bias was detected. A. incidence of all-grade FACs; B. incidence of grade 3 or higher FACs; C. incidence of grade 1-2 FACs; D. incidence of cardiac ischemia; E. incidence of arrhythmia; F. incidence of ECG changes.

Supplementary Fig. S9

Forest plot for the sensitivity analysis of main outcomes. A. incidence of all-grade FACs; B. incidence of grade 3 or higher FACs; C. incidence of grade 1-2 FACs; D. incidence of cardiac ischemia; E. incidence of arrhythmia; F. incidence of ECG changes.
