# Supplementary material for: Redefining the Incidence and Profile of Fluoropyrimidine-Associated Cardiotoxicity in Cancer Patients: A Systematic Review and Meta-Analysis
Source: Pharmaceuticals (Basel). 2023 Mar 30;16(4):510. doi: 10.3390/ph16040510 (PMC10146083; doi:10.3390/ph16040510)
Supplement: Supplementary file 1 [file pharmaceuticals-16-00510-s001.zip › Supplementary Table S1-S3 .pdf]

Table S1 The retrieval scheme used for each database [a,b]

**PubMed**	
#1	heart[Title/Abstract] OR cardiac[Title/Abstract] OR cardiovascular[Title/Abstract] OR cardiotoxicity[Title/Abstract]
#2	myocardial infarction[Title/Abstract] OR ischemia[Title/Abstract] OR coronary artery[Title/Abstract] OR acute coronary syndrome[Title/Abstract] OR coronary vasospasm[Title/Abstract] OR chest pain[Title/Abstract] OR angina[Title/Abstract] OR syncope[Title/Abstract] OR dyspnea[Title/Abstract] OR myocardium[Title/Abstract]OR palpitation[Title/Abstract] OR ventricular dysfunction[Title/Abstract] OR systolic dysfunction[Title/Abstract] OR heart failure[Title/Abstract] OR LVEF[Title/Abstract] OR atrioventricular block[Title/Abstract] OR conduction disorder[Title/Abstract] OR bundle branch block[Title/Abstract] OR arrhythmia[Title/Abstract] OR tachycardia[Title/Abstract] OR bradycardia[Title/Abstract] OR atrial fibrillation[Title/Abstract] OR ventricular fibrillation[Title/Abstract] OR repolarization[Title/Abstract] OR atrial flutter[Title/Abstract] OR ECG[Title/Abstract] OR pericardial effusion[Title/Abstract] OR pericarditis[Title/Abstract] OR pericardial tamponade[Title/Abstract] OR cardiac myositis[Title/Abstract] OR myocarditis[Title/Abstract] OR cardiomyopathy[Title/Abstract] OR heart-sac[Title/Abstract] OR cardiac arrest[Title/Abstract] OR sudden death[Title/Abstract] OR cardiogenic shock[Title/Abstract] OR regurgitation[Title/Abstract] OR echocardiography[Title/Abstract] OR cardiac biomarkers[Title/Abstract] OR troponin[Title/Abstract] OR enzyme[Title/Abstract] OR death[Title/Abstract]
#3	#1 OR #2
#4	fluoropyridine[Title/Abstract] OR fluoropyrimidine[Title/Abstract] OR 5-fluorouracil[Title/Abstract] OR 5-FU[Title/Abstract] OR fluorouracil[Title/Abstract] OR capecitabine[Title/Abstract] OR xeloda[Title/Abstract] OR tegafur[Title/Abstract] OR S-1[Title/Abstract] OR teysuno[Title/Abstract] OR UFT[Title/Abstract] OR carmofur[Title/Abstract] OR TAS-102[Title/Abstract] OR lonsurf[Title/Abstract] OR FTD/TPI[Title/Abstract] OR trifluridine[Title/Abstract] OR tipiracil[Title/Abstract]
#5	#3 AND #4

**Medline**	
#1	(heart or cardiac or cardiovascular or cardiotoxicity).ab.
#2	(myocardial infarction or ischemia or coronary artery or acute coronary syndrome or coronary vasospasm or chest pain or angina or syncope or dyspnea or myocardiumor palpitation or ventricular dysfunction or systolic dysfunction or heart failure or LVEF or atrioventricular block or conduction disorder or bundle branch block or arrhythmia or tachycardia or bradycardia or atrial fibrillation or ventricular fibrillation or repolarization or atrial flutter or ECG or pericardial effusion or pericarditis or

	pericardial tamponade or cardiac myositis or myocarditis or cardiomyopathy or heart-sac or cardiac arrest or sudden death or cardiogenic shock or regurgitation or echocardiography or cardiac biomarkers or troponin or enzyme or death).ab.
#3	#1 or #2
#4	(fluoropyridine or fluoropyrimidine or 5-fluorouracil or 5-FU or fluorouracil or capecitabine or xeloda or tegafur or S-1 or teysuno or UFT or carmofur or TAS-102 or lonsurf or FTD TPI or trifluridine or tipiracil).ab.
#5	#3 and #4

**Embase**	
#1	heart:ab OR cardiac:ab OR cardiovascular:ab OR cardiotoxicity:ab
#2	(((((((((((((((((((((myocardial:ab AND infarction:ab OR ischemia:ab OR coronary:ab) AND artery:ab OR acute:ab) AND coronary:ab AND syndrome:ab OR coronary:ab) AND vasospasm:ab OR chest:ab) AND pain:ab OR angina:ab OR syncope:ab OR dyspnea:ab OR myocardiumor:ab) AND palpitation:ab OR ventricular:ab) AND dysfunction:ab OR systolic:ab) AND dysfunction:ab OR heart:ab) AND failure:ab OR lvef:ab OR atrioventricular:ab) AND block:ab OR conduction:ab) AND disorder:ab OR bundle:ab) AND branch:ab AND block:ab OR arrhythmia:ab OR tachycardia:ab OR bradycardia:ab OR atrial:ab) AND fibrillation:ab OR ventricular:ab) AND fibrillation:ab OR repolarization:ab OR atrial:ab) AND flutter:ab OR ecg:ab OR pericardial:ab) AND effusion:ab OR pericarditis:ab OR pericardial:ab) AND tamponade:ab OR cardiac:ab) AND myositis:ab OR myocarditis:ab OR cardiomyopathy:ab OR 'heart sac':ab OR cardiac:ab) AND arrest:ab OR sudden:ab) AND death:ab OR cardiogenic:ab) AND shock:ab OR regurgitation:ab OR echocardiography:ab OR cardiac:ab) AND biomarkers:ab OR troponin:ab OR enzyme:ab OR death:ab
#3	#1 OR #2
#4	fluoropyridine:ab OR fluoropyrimidine:ab OR '5 fluorouracil':ab OR '5 fu':ab OR fluorouracil:ab OR capecitabine:ab OR xeloda:ab OR tegafur:ab OR 's 1':ab OR teysuno:ab OR uft:ab OR carmofur:ab OR 'tas 102':ab OR lonsurf:ab OR ftd:ab OR tpi:ab OR trifluridine:ab OR tipiracil:ab
#5	#3 AND #4

**Web of Science**	
#1	AB=(heart or cardiac or cardiovascular or cardiotoxicity)
#2	AB=(myocardial infarction or ischemia or coronary artery or acute coronary syndrome or coronary vasospasm or chest pain or angina or syncope or dyspnea or myocardiumor palpitation or ventricular dysfunction or systolic dysfunction or heart failure or LVEF or atrioventricular block or conduction disorder or bundle branch block or arrhythmia or tachycardia or bradycardia or atrial fibrillation or ventricular fibrillation or

	repolarization or atrial flutter or ECG or pericardial effusion or pericarditis or pericardial tamponade or cardiac myositis or myocarditis or cardiomyopathy or heart-sac or cardiac arrest or sudden death or cardiogenic shock or regurgitation or echocardiography or cardiac biomarkers or troponin or enzyme or death)
#3	(#1) OR #2
#4	AB=(fluoropyridine or fluoropyrimidine or 5-fluorouracil or 5-FU or fluorouracil or capecitabine or xeloda or tegafur or S-1 or teysuno or UFT or carmofur or TAS-102 or lonsurf or FTD TPI or trifluridine or tipiracil)
#5	(#3) AND #4

Cochrane database

#1	(heart or cardiac or cardiovascular or cardiotoxicity):ab
#2	(myocardial infarction or ischemia or coronary artery or acute coronary syndrome or coronary vasospasm or chest pain or angina or syncope or dyspnea or myocardiumor palpitation or ventricular dysfunction or systolic dysfunction or heart failure or LVEF or atrioventricular block or conduction disorder or bundle branch block or arrhythmia or tachycardia or bradycardia or atrial fibrillation or ventricular fibrillation or repolarization or atrial flutter or ECG or pericardial effusion or pericarditis or pericardial tamponade or cardiac myositis or myocarditis or cardiomyopathy or heart-sac or cardiac arrest or sudden death or cardiogenic shock or regurgitation or echocardiography or cardiac biomarkers or troponin or enzyme or death):ab
#3	#1 OR #2
#4	(fluoropyridine or fluoropyrimidine or fluorouracil or capecitabine or xeloda or tegafur or S-1 or teysuno or UFT or carmofur or TAS-102 or lonsurf or FTD TPI or trifluridine or tipiracil):ab
#5	#3 AND #4

Clnical trials (https://clinicaltrials.gov/)

- fluoropyrimidine
- 5-FU OR 5-fluorouracil OR fluorouracil
- capecitabine
- tegafur
- S-1
- TAS-102 OR FTD/TPI OR lonsurf

Notes：a, The search algorithms used in this study are presented below. All databases were searched from their inception until 31 March 2021; b,Searching strategy combined two sorts of terms using the logic operator “AND”: (1) fluoropyrimidine agents, including 5-FU, capecitabine, S-1, and tipiracil hydrochloride (TAS-102); (2) treatment-related cardiac toxicities, including myocardial ischemia, acute coronary syndrome, arrhythmia, angina, heart failure, ECG changes, and so on.

Table S2 The National Institutes of Health (NIH) quality assessment tools

Website: https://www.nhlbi.nih.gov/health-topics/study-quality-assessment-tools

1. The National Institutes of Health (NIH) quality assessment tool for case-series study

**Major Components**	**Response**	**Options**	
1. Was the study question or objective clearly stated?	Yes	No	Cannot Determine/ Not Applicable/ Not Reported
2. Was the study population clearly and fully described, including a case definition?	Yes	No	Cannot Determine/ Not Applicable/ Not Reported
3. Were the cases consecutive?	Yes	No	Cannot Determine/ Not Applicable/ Not Reported
4. Were the subjects comparable?	Yes	No	Cannot Determine/ Not Applicable/ Not Reported
5. Was the intervention clearly described?	Yes	No	Cannot Determine/ Not Applicable/ Not Reported
6. Were the outcome measures clearly defined, valid, reliable, and implemented consistently across all study participants?	Yes	No	Cannot Determine/ Not Applicable/ Not Reported
7. Was the length of follow-up adequate?	Yes	No	Cannot Determine/ Not Applicable/ Not Reported
8. Were the statistical methods well-described?	Yes	No	Cannot Determine/ Not Applicable/ Not Reported
9. Were the results well-described?	Yes	No	Cannot Determine/ Not Applicable/ Not Reported
**Quality Rating**	**Good**	**Fair**	**Poor**

Additional Comments (If Poor, please state why):

2. The National Institutes of Health (NIH) quality assessment tool of controlled intervention study

**Major Components**	**Response options**

1. Was the study described as randomized, a randomized trial, a randomized clinical trial, or an RCT?	Yes	No	Cannot Determine/ Not Applicable/ Not Reported
2. Was the method of randomization adequate (i.e., use of randomly generated assignment)?	Yes	No	Cannot Determine/ Not Applicable/ Not Reported
3. Was the treatment allocation concealed (so that assignments could not be predicted)?	Yes	No	Cannot Determine/ Not Applicable/ Not Reported
4. Were study participants and providers blinded to treatment group assignment?	Yes	No	Cannot Determine/ Not Applicable/ Not Reported
5. Were the people assessing the outcomes blinded to the participants' group assignments?	Yes	No	Cannot Determine/ Not Applicable/ Not Reported
6. Were the groups similar at baseline on important characteristics that could affect outcomes (e.g., demographics, risk factors, co-morbid conditions)?	Yes	No	Cannot Determine/ Not Applicable/ Not Reported
7. Was the overall drop-out rate from the study at endpoint 20% or lower of the number allocated to treatment?	Yes	No	Cannot Determine/ Not Applicable/ Not Reported
8. Was the differential drop-out rate (between treatment groups) at endpoint 15 percentage points or lower?	Yes	No	Cannot Determine/ Not Applicable/ Not Reported
9. Was there high adherence to the intervention protocols for each treatment group?	Yes	No	Cannot Determine/ Not Applicable/ Not Reported
10. Were other interventions avoided or similar in the groups (e.g., similar background treatments)?	Yes	No	Cannot Determine/ Not Applicable/ Not Reported
11. Were outcomes assessed using valid and reliable measures, implemented consistently across all study participants?	Yes	No	Cannot Determine/ Not Applicable/ Not Reported
12. Did the authors report that the sample size was sufficiently large to be able to detect a difference in the main outcome between groups with at least 80% power?	Yes	No	Cannot Determine/ Not Applicable/ Not Reported
13. Were outcomes reported or subgroups analyzed prespecified (i.e., identified before analyses were conducted)?	Yes	No	Cannot Determine/ Not Applicable/ Not Reported
14. Were all randomized participants analyzed in the group to which they were originally assigned, i.e., did they use an intention-to-treat analysis?	Yes	No	Cannot Determine/ Not Applicable/ Not Reported

**Quality Rating**	Good Fair Poor

Additional Comments (If Poor, please state why):

Table S3 List of the included 211 studies

No.	Author	Year	Country/Region	Size	Age	Gender (female × 100%)	Tumor type	Regimen	Quality
1	Kachnic LA	2022	USA	52	58(34-82)	0.808	Annal cancer	5-FU/mitomycin-C+ RT	Fair
2	Yamaguchi K	2022	Japan	54<br>46	66(32-75)<br>65(30-75)	0.204<br>0.391	Gastric/gastroesophageal junction cancer	SOX+ Pembrolizumab<br>SP	Good
3	Conroy T	2021	France	163	61(53-66)	0.35	Rectal cancer	FOLFIRINOX+ RT	Good
4	Hofheinz RD	2021	Germany	56	62(32-86)	0.268	Esophagogastric adenocarcinoma	FLOT+ Tra	Fair
5	Meyerhardt JA	2021	USA	1261<br>1263	61(19.3-86.5)<br>61.7(21.8-88.7)	0.445<br>0.454	Colon cancer	FOLFOX<br>FOLFOX+ Celecoxib	Good
6	Mayer IA	2021	USA	198	52(26-76)	1	Breast cancer	Cap	Good
7	Chakravarthy AB	2020	USA	176<br>179	54.3±11.7<br>53.9±9.9	0.348<br>0.376	Rectal cancer	mFOLFOX<br>mFOLFOX+Bev	Fair
8	Dyhl-Polk A	2020	Denmark	108	66(35-81)	0.454	Colorectal or anal cancer	Coloretal cancer: 5-Fu or FOLFOX;<br>Metastatic: FOLFOX or FOLFIRI± Cet or Pan<br>Anal cancer: FP+ RT	Fair
9	Aimar G	2020	Italy	135	66	0.393	Colorectal cancer	Fluoropyrimidine	Good
10	Osterlund P	2020	Multi-country:	141	NA	NA	Mixed malignancies[a]	S1	Fair

			Finland; Netherland; Denmark; Sweden; Ireland; Norway						
11	Rajendra A	2020	Indian	246	NA	NA	Head and neck cancer	TPF	Fair
12	Delaloge S	2020	Multi-country: France; Netherlands; Belgium; Spain; USA; Italy; Germany; Portugal	628	18-70	1	Breast cancer	TX	Good
13	Grierson P	2020	USA	16	66(42-73)	0.563	Pancreatic ductal adenocarcinoma	Cap+ Tosedostat	Fair
14	Dyhl-Polk A	2020	Denmark	995<br>1241	65(21-85)<br>70(22-93)	0.447<br>0.471	Colorectal cancer	5-FU based<br>Cap based	Good
15	Gibson MK	2019	USA	22	62(45-79)	0.091	Esophagus adenocarcinoma	Oxaliplatin/ 5-Fu+ Cet+ RT	Fair
16	Raber I	2019	USA	177	54-77	0.452	Mixed malignancies	5-FU or Cap based	Fair
17	Shanmuganathan JWD	2019	Denmark	9012	NA	NA	Mixed malignancies	5-FU	Fair
18	Lombardi P	2019	Italy	101	NA	0.346	Colorectal cancer	Fluoropyrimidine[b]	Good
19	Jin X	2019	China	129	>18	0.217	Gastric cancer	5-FU or Cape or S-1 based	Fair

20	Rivera F	2019	Spain	45	65(45-80)	0.177	Gastric or gastro-oesophageal junction (egj) cancer	XELOX+ Tra	Fair
21	Primrose JN	2019	UK	213	62(55-68)	0.5	Biliary tract cancer	Cap	Good
22	Abdel-Rahman O	2019	Canada	3223	60.7(11.4)	0.403	Colorectal cancer	FOLFOX or Flu based+ Bev and/or Pan	Good
23	Hayashi Y	2019	Japan	80	66.5(62-73)	0.113	Esophageal cancer	5-FU/ cisplatin+ RT	Fair
24	Peng J	2018	China	527	57(23-87)	0.339	Mixed malignancies	5-FU or Cap based	Good
25	Chen EY	2018	China	47	59.7(21.4-80.1)	0.276	Colorectal cancer	FOLFIRI+ Celecoxib	Good
26	Yang H	2018	China	30<br>30	20-80	NA	Gastric cancer (liver metastasis)	SOX+ Endostar<br>SOX	Fair
27	Alderson D	2017	UK	446<br>441	62(57-67)<br>62(56-67)	0.108<br>0.086	Esophageal adenocarcinoma	FP<br>ECX	Good
28	Kwakman JJM	2017	Netherlands	1973	NA	NA	Colorectal cancer	Cap mono or based +/- Bev	Good
29	Turan T	2017	turkey	32	57	0.303	Mixed malignancies	5-FU based	Good
30	Leicher LW	2017	Netherland	86	69(45-83)	0.523	Colorectal cancer	Cap	Fair
31	Zhang P	2017	China	267<br>130	25–70	1	Breast cancer	Cap+ Utidelone<br>Cap	Good
32	Harbeck N	2017	Germany	102	63(22–85)	1	Breast cancer	Cap	Good
33	Kerr RS	2016	Multi-country: Australia, Austria, Czech Republic, New Zealand,	973<br>968	65(58-71)	0.4270.429	Colorectal cancer	Cap+Bev<br>Cap	Good

			Serbia, Slovenia, UK						
34	Lestuzzi C	2016	Italy	192	62(37-85)	0.401	Mixed malignancies	Cap based	Fair
35	Winther SB	2016	Norway	71	67-87	0.408	Colorectal cancer	SOX or S-1	Fair
36	Polk A	2016	Denmark	452	63(28-88)	1	Breast cancer	Cap+ Tra	Fair
37	Mayer RJ	2015	USA	534	63(27-82)	0.389	Colorectal cancer	TAS102	Good
38	van der Sluis PC	2015	Netherland	93<br>38	63(36-78)	0.14	Esophageal or gastro-esophageal junction adenocarcinoma	ECX<br>ECX	Fair
39	Lestuzzi C	2014	Germany	358	57.5(23-80)	NA	Mixed malignancies	5-FU or 5-FU based	Fair
40	Tonyali O	2013	Turkey	37	46(30-75)	1	Breast cancer	TX+ Tra	Fair
41	Okines AFC	2013	UK	101<br>99	62(56-67)<br>64(56-69)	0.321<br>0.182	Gastro-esophageal adenocarcinoma	ECX<br>ECX+ Bev	Good
42	Uygun K	2013	turkey	68<br>64	53.6(21-79)<br>59.7(37-79)	0.414	Colorectal cancer	XELIRI+ Bev<br>FOLFIRI+ Bev	Fair
43	Mackey JR	2013	Multi-country: Canada, Spain, Poland, France, Hungary, UK, Sweden , USA, Brazil	736	49(43–56)	1	Breast cancer	FAC	Good
44	Souglakos J	2012	Greece	166<br>167	67(26-80)<br>66(33-80)	0.343<br>0.377	Colorectal cancer	CapIRI+ Bev<br>FOLFIRI+ Bev	Good
45	Cetin B	2012	Turkey	203	47(25-72)	1	Breast cancer	Cap+ Lap	Fair
46	Lang I	2012	Multi-country: Hungary, Israel,	277	59(27-86)	0.986	Breast cancer	Cap+ Bev	good

			Austria, Bosnia and Herzegovina, Latvia I, Germany						
47	Nishimura Y	2012	Japan	42<br>45	63(45-74)<br>63(48-74)	0.108<br>0.088	Esophageal cancer	FP+RT<br>FP+RT	Good
48	Cen P	2012	USA, SEER data	5472	74.2(65–99)	0.504	Colorectal cancer	5-FU	Fair
49	Khan MA	2012	Pakistani	301	47(18-81)	0.249	Mixed malignancies	5-FU or 5-FU/Cap based	Fair
50	Martin M	2012	Multi-country: Spain, Russia, France, Switzerland	88	53(32-82)	0.988	Breast cancer	Cap+ Bev+ Tra	Fair
51	Petrini L	2012	Italy	39	67(41-83)	0.154	Hepatocellular carcinoma	5-FU+ Sorafenib	Good
52	Lopez L	2012	Brazil	240	NA	NA	Gastrointestinal cancer	5-FU or 5-FU based	Fair
53	Dipetrillo T	2012	USA	26	50(39-75)	NA	Rectal adenocarcinoma	mFOLFOX6+Bev	Fiar
54	Greil R	2011	Multi-country: Slovak republic, Czech republic, Austria, Slovenia, Poland, Ukraine, Romania, Bulgaria, Cyprus, Malta, Estonia, Russia	293	52(27-77)	1	Breast cancer	Cap+ Lap	Fair
55	Robert NJ	2011	Multi-country: USA, France, Ukraine, Russia,	201<br>404	57(23-88)<br>56(28-91)	1	Breast cancer	Cap<br>Cap+ Bev	Good

			UK						
56	Koca D	2011	Turkey	52	59	0.75	Mixed malignancies	Cap or Cap based+ Lap	Fair
57	Jensen SA	2011	Denmark	106	64(37-81)	0.556	Colorectal cancer	FOLFOX4	Good
58	Kato K	2010	Japan	76	61(39-70)	0.105	Esophageal squamous cell carcinoma	FP+RT	Fair
59	Wildiers H	2010	Belgium	53<br>50	51(25-74)<br>50.5(25-76)	1	Breast cancer	TX<br>TX+ Tra	Fair
60	Torrisi R	2010	Italy	32	47(28-69)	1	Breast cancer	CCF+ Tra	Fair
61	Masi G	2010	Italy	57	61(34-75)	0.4	Colorectal cancer	FOLFOXIRI+ Bev	Good
62	Michalaki V	2010	Greece	29	52(34-70)	1	Breast cancer	TX+ Tra	Good
63	Chua YJ	2010	Australia	105	64(54–70)	0.46	Rectal cancer	XELOX	Good
64	Wardley AM	2010	Multi-country: Mexico, Brazil, Costa Rica, Poland, Switzerland, Australia, Spain	112	53(24-82)	1	Breast cancer	TX+ Tra or Taxane+ Tra	Good
65	Baur M	2010	Austria	71	62(39-84)	0.394	Rectal cancer	5-FU based	Fair
66	Hu W	2010	China	34<br>37	62(55-69)	1	Breast cancer	Cap<br>CEF	Fair
67	Martin M	2010	Multi-country: Spain, Germany, and Poland	521	49(23-73)	1	Breast cancer	FAC	Good
68	Joensuu H	2009	Multi-country: Helsinki, Tampere,	115<br>116	≤65	1	Breast cancer	5-FU based+ Tra<br>5-FU based	Good

			Hameenlinna, Pori, Oulu, Turku, Kotka, Vaasa, Finland						
69	Osawa S	2009	Japan	33	66(55-82)	0.242	Esophageal squamous cell carcinoma	Nedaplatin/5-FU+ RT	Fair
70	Pisano C	2009	Italy	36	60(42-76)	NA	Ovarian cancer	Cap	Fair
71	Bathe OF	2009	Canada	35	59±8	0.314	Colorectal cancer	FOLFIRI	Fair
72	Skof E	2009	Slovenia	41<br>46	63(47-75)<br>62(34-75)	0.366	Colorectal cancer	XELIRI<br>FOLFIRI	Good
73	Giuliani F	2008	Italy	41	63(44-73)	0.366	Gastric cancer	EDF	Fair
74	Ardavanis A	2008	Greece	34	69.5(37-83)	0.47	Colorectal cancer	CapIRI+ Bev	Fair
75	Kosmas C	2008	Greece	644	66(56-70)	NA	Mixed malignancies	5-FU based or Cap based	Good
76	Rothenberg ML	2008	Multi-country: USA, Canada, Spain, Republic of Korea, Switzerland, UK	311<br>308	59.7(26-83)<br>60.7(26-81)	0.38	Colorectal cancer	XELOX<br>FOLFOX-4	Good
77	Yamamoto D	2007	Japan	59	55(42-70)	1	Breast cancer	Cap+ Tra	Good
78	Natoli C	2007	Italy	44	18-65	1	Breast cancer	Epirubicin/ Cyclophosphamide/ Docetaxel/ Cap	Fair
79	Neri B	2007	Italy	36	58(39-72)	0.305	Gastric carcinoma	5-FU/ Epirubicin	Fair

80	Machiels JP	2007	Belgium	40	61(34-78)	0.33	Rectal cancer	Cap+ Cet+ RT	Fair
81	Sasamoto R	2007	Japan	68<br>68	40–79(65)	0.103	Esophageal cancer	FP+RT	Fair
82	Buzdar AU	2007	USA	19<br>45	48(25-75)<br>52(29-71)	1	Breast cancer	Paclitaxel+ FEC<br>Paclitaxe+ FEC+ Tra	Fair
83	Yilmaz U	2007	Turkey	27	54(19–70)	0.444	Gastrointestinal cancer	LV5FU2	Fair
84	ECOG-E3205/ Garg MK[c]	2007/2017	USA	62	56(37-48)	0.803	Anal carcinoma	FP+ Cet+ RT	Good
85	Rossi D	2007	Italy	37	62(38-87)	1	Breast cancer	Cap	Fair
86	Emmanouilides C	2007	Greece	53	65(18–78)	0.434	Colorectal cancer	FOLFOX+ Bev	Fair
87	Geyer CE	2006	USA	163<br>161	54(26-80)<br>51(28-83)	1	Breast cancer	Cap+ Lap<br>Cape	Good
88	Mambrini A	2006	Italy	211	61(21-79)	NA	Pancreatic cancer	FEC	Good
89	Thatai LC	2006	USA	39	49(29–66)	1	Breast cancer	TAF	Fair
90	Koopman M	2006	Netherland	199<br>194	64(27-84)<br>63(35-79)	0.373<br>0.396	Colorectal cancer	Cap<br>CapIRI	Good
91	Jensen SA	2006	Denmark	214<br>81<br>352<br>10<br>11	NA	NA	Colorectal or gastric cancers	Cap<br>Cap/ Capatin/ Docetaxel<br>5-FU<br>LV5FU2<br>FOLFOX-4	Fair
92	Velenik V	2006	Slovenia	57	67(34-81)	0.245	Rectal adenocarcinoma	Cap+ RT	Fair

93	Rapidis AD	2006	Greece	42	58(31-70)	0.166	Head and neck carcinoma	TPF+ RT	Fair
94	Yerushalmi R	2006	Israel	43<br>46	66(25-82)<br>62(23-81)	0.418<br>0.5	Rectal cancer	Cap+ RT<br>5-FU+RT	Fair
95	Tsibiribi P	2006	France	1350	NA	NA	Mixed malignancies	5-FU	Fair
96	Klautke G	2006	Germany	28	64(48-75)	0.571	Rectal cancer	CapIRI+RT	Fair
97	Giordano KF	2006	USA	44	57(32–77)	0.114	Gastric or gastro-esophageal junction adenocarcinoma	TX	Fair
98	ECOG-E2200/Giantonio BJ[c]	2006	USA	87	59(29.7-84.9)	0.395	Colorectal cancer	FOLFIRI	Good
99	Jatoi A	2006	USA	46	61(32–80)	0.116	Esophageal or gastro-esophageal junction adenocarcinoma	XELOX	Fair
100	Baghi M	2006	Germany	24	60(23-79)	0.042	Head and neck squamous cell carcinoma	TPF	Fair
101	ECOG-E2204/ Berlin DJ[c]	2006/2018	USA	130	59(29.2-92.9)	0.5	Pancreatic cancer	Cap/ Gemcitabine/+ Cet or Bev +- RT	Good
102	ECOG-E3204/ Landry JC[c]	2006/2015	USA	55	NA	NA	Rectal cancer	XELOX+ Bev+ RT	Fair
103	Gianni L	2005	Multi-country: Italy, Spain, Germany, Russia, Poland, Hungary, Czech Republic	1233	NA	1	Breast cancer	Doxorubicin/ Docetaxel/ CMF	Fair

104	Meydan N	2005	Turkey	231	59(23-87)	0.402	Mixed malignancies	LV5FU2	Fair
105	ECOG-E4203/ Meropol NJ[c]	2005/2018	USA	205	60(29-85)	0.403	Colorectal cancer	mFOLFOX6+Bev	Fair
106	Tsavaris N	2005	Greece	522	NA	NA	Mixed malignancies	5-FU based	Fair
107	Ceyhan C	2005	Turkey	37	62(22-83)	0.297	Mixed malignancies	HDLV5-FU	Fair
108	Bontenbal M	2005	Netherlands	107	54(31-70)	1	Breast cancer	FAC	Good
109	Levine MN	2005	Canada	359<br>351	40-49	1	Breast cancer:	CMF+ RT<br>CEF+ RT	Good
110	Lordick F	2005	Germany	48	62(41-75)	0.187	Gastric cancer	FOLFOX	Fair
111	Ng M	2005	UK	153	33–81	0.412	Colorectal cancer	CapeOx	Good
112	Smaradottir A	2005	USA	12	64	0.083	Head and neck squamous cell carcinoma	TPF	Fair
113	Keene KS	2005	USA	28	64(41–82)	0.571	Pancreatic adenocarcinoma	5-FU+RT	Fair
114	Feliu J	2005	Spain	51	76(71-89)	0.392	Colorectal cancer	Cap	Fair
115	Berruti A	2005	Italy	61	54.3(30.6–70.7)	1	Breast cancer	Vinorelbine/ Paclitaxel/ 5-FU	Fair
116	Erman M	2005	Turkey	88	47±10	1	Breast cancer	FEC+T	Fair
117	Berruti A	2005	Italy	53	58.9(30.4-75.8)	1	Breast cancer	Vinorelbine/ Paclitaxel/ 5-FU	Fair
118	ECOG-E1103/ Li TH[c]	2004/2012	USA	68	52(35-79)	1	Breast cancer	Cap+ Tipifarnib	Good
119	Oztop I	2004	Turkey	22	63(44-76)	0.364	Gastrointestinal cancer	LV5FU2	Fair

120	Martin M	2003	Spain	505<br>480	18-72	1	Breast cancer	CMF<br>FAC	Good
121	Daniele B	2003	Italy	34	76(70–85)	0.25	Colorectal cancer	LV5FU2	Fair
122	Elomaa I	2003	Finland	40	53(32-69)	1	Breast cancer	FNE	Fair
123	Wacker A	2003	Germany	102	61.7(39-78)	0.311	Mixed malignancies	5-FU or 5-FU based	Fair
124	ECOG-E3201	2003/2018	USA	59<br>58<br>62	NA	0.322<br>0.379<br>0.306	Rectal cancer	FOLFIRI<br>mFOLFOX6<br>LV5FU2	Good
125	Hitt R	2002	Spain	70	61(37-75)	0.014	Head and neck squamous cell carcinoma	TPF	Fair
126	Vaishampayan UN	2002	USA	32	67.5(45–84)	0.375	Gastrointestinal cancer	Cap+ RT	Fair
127	Tsavaris N	2002	Greece	427	NA	NA	Mixed malignancies	5-Fu based	Fair
128	Van Cutsem E	2002	Switzerland	593<br>596<br>236	NA<br>NA<br>NA	NA	Colorectal cancer<br>Colorectal cancer<br>Breast cancer	LV5FU2<br>Cap<br>Cap	Fair
129	ECOG-E3200/ Giantonio BJ[c]	2001/2007	USA	808<br>286<br>291	21-85<br>62(21-85)<br>60.8(25-84)	0.39<br>0.395<br>0.392	Colorectal cancer	mFOLFOX6+Bev<br>FOLFOX4 +Bev<br>FOLFOX4	Good
130	Hartung G	2001	Germany	51	60(24-77)	0.25	Colorectal cancer	LV5FU2	Fair
131	Dencausse Y	2001	Germany	21	30-80	0.333	Rectal cancer	LV5FU2+RT	Fair
132	Recchia F	2001	Italy	28	62(28-75)	1	Breast cancer	FE	Fair
133	Riccardi A	2001	Italy	69	54(34-70)	1	Breast cancer	FEC+T	Fair
134	Piccart MJ	2001	Belgium	255	≤70	1	Breast cancer	CMF	Fair

135	Jassem J	2001	Multi-country: Poland, Yugoslavia, Russia, Croatia, Latvia, Hungary, Israel, Belgium	123	50(24-74)	1	Breast cancer	FAC	Good
136	Peiffert D	2001	France	80	≤75	0.837	Anal cancer	FP+RT	Fair
137	Ackland SP	2001	Multi-country: Australia, Bulgaria, Roma, Spain, Italy, Germany, Russia, Uruguay, Hungary	218<br>236	56(22-71)<br>55(26-71)	1	Breast cancer	CEF<br>CMF	Good
138	Pienkowski T	2001	Poland	65	48(31-70)	1	Breast cancer	FN	Fair
139	Blum JL	2001	USA	74	52(29-77)	1	Breast cancer	Cape	Fair
140	Hoff PM	2001	Multi-country: USA, Canada, UK, Switzerland	302<br>303	64(23-86)<br>63(24-87)	0.40<br>0.35	Colorectal cancer	Cape<br>LV5FU2	Good
141	Lin JK	2000	China Taiwan	40	66(27-78)	0.425	Colorectal cancer	S-1	Fair
142	Zambelli A	2000	Italy	48	52(29-72)	1	Breast cancer	FN	Fair
143	Balloni L	2000	Italy	25	NA	NA	Colon cancer	5-FU	Fair
144	Susvnjar S	1999	Yugoslavia	19	47(32-64)	1	Breast cancer	LV5FU2/ Mitoxantrone	Fair
145	Valero V	1999	USA	41	≥18	1	Breast cancer	FAC	Fair
146	Hasbini A	1999	Spain	44	45(20-72)	0.174	Nasopharyngeal carcinoma	FMEP	Fair
147	Blum JL	1999	Multi-country:	162	55.8(26-78)	1	Breast cancer	Cap	Fair

			USA, Canada						
148	Blajman C	1999	Argentina	85	54(30–71)	1	Breast carcinoma	FAC	Fair
149	Birkenhake S	1999	Germany	25	62(31-79)	0.25	Bladder cancer	FP+RT	Fair
150	Milano G	1999	French	19	58(36-73)	0.75	Mixed malignancies	5-FU based	Fair
151	Warner E	1999	Canada	30	60(41–81)	0.1	Esophageal carcinoma	FP+LV	Fair
152	Andersson M	1999	Denmark	161	58(45-65)	1	Breast cancer	CAF	Good
				181	58(43-65)			CMF	
153	Mantovani G	1998	Italy	16	54.4(38–72)	0.125	Head and neck squamous-cell carcinoma	FP+ IL-2	Good
				17	56.75(48-66)	0.117		FP	
154	Papadimitriou CA	1998	Greece	24	56(35-71)	1	Breast cancer	CMF+ Tra	Fair
155	Katona C	1998	Hungary	96	63(43-77)	0.312	Colorectal cancer	LV5FU2	Fair
156	Levine MN	1998	Canada	256	40-49	1	Breast cancer	CEF	Good
				360				CMF	
157	Fetting JH	1998	USA	307	48(25-77)	1	Breast cancer:	CAF	Good
				309	47(25-78)			CMF	
158	Seitz JF	1998	France	24	59.7(41-73)	0.542	Colorectal carcinoma	LV5FU2+ Mitomycin C	Fair
159	Tominaga T	1998	Japan	20	40-70	1	Breast cancer	FAC	Fair
160	Wang WS	1998	China Taiwan	41	62(35-68)	0.195	Colorectal carcinoma	LV5FU2	Good
161	Taylor SG	1997	USA	78	61(30–83)	0.269	Head and neck cancer	Cisplatin/5-FU+RT	Fair
162	Yen CC	1997	China Taiwan	13	55	1	Breast cancer	MFL	Fair
163	Meyer CC	1997	USA	483	60.9±11.9	0.407	Mixed malignancies	5-FU based	Fair
164	Bascioni R	1997	Italy	46	63(34–78)	0.018	Breast cancer	MFL	Fair
165	Kroning	1997	Japan	27	NA	NA	Gastric carcinoma	LV5FU2/ Epirubicin	Fair

	KC								
166	Doci R	1996	Italy	35	59(31-80)	0.857	Anal cancer	FP+RT	Fair
167	Ychou M	1996	France	28	64(40-78)	0.143	Gastric cancer	FP/ LV	Fair
168	Kok TC	1996	Netherlands	29	60(41-74)	0.138	Esophageal or esophago-gastric junction adenocarcinoma	LV5FU2	Fair
169	Gebbia V	1996	Italy	33	62(46-75)	0.242	Colorectal cancer	5-FU/ LV/ IL-2	Fair
170	Hainsworth JD	1996	USA	46	52,32-74	1	Breast cancer	Paclitaxel/ Mitoxantrone/ 5-FU/ LV	Fair
171	Hartung G	1996	Germany	16<br>29	54.4<br>59.7	0.5<br>0.379	Colorectal cancer	5-FU/ Levamisole<br>5-FU/ LV	Fair
172	Colozza M	1996	Italy	28	57(42-76)	1	Breast cancer	LV5FU2/ Mitoxantrone	Fair
173	Mammoliti S	1996	Italy	24	65(65-75)	1	Breast cancer	LV5FU2/ Mitoxantrone	Fair
174	Becouarn YH	1996	France	86	62(39-82)	0.512	Colorectal cancer	LV5FU2	Fair
175	Blijham GH	1996	Europe	312	NA	NA	Colorectal cancer	5-FU/ Methotrexate	Fair
176	Macdonald JS	1995	USA	90	59(27-75)	0.366	Gastric carcinoma.	FAM	Fair
177	Weidmann B	1995	Germany	231	NA	NA	Mixed malignancies	5-FU based	Fair
178	Kolaric K	1995	Austria	237	50.5(33-71)	1	Breast cancer	FAC/ Cardioxane(ICRF-187)	Fair
179	Alonso MC	1995	Spain	50<br>50	55(30-72)<br>55(31-74)	1	Breast cancer	CNF<br>CAF	Good
180	Leonardi V	1995	Italy	34	56(33-74)	1	Breast cancer	CNF+LV	Fair

181	Carmo-Pereira J	1995	Portugal	38	50.5(34-65)	1	Breast carcinoma	CEF	Fair
182	Haas NB	1995	USA	38	62.5(38-76)	0.368	Colorectal cancer	LV5FU2	Fair
183	Klastersky J	1994	Multi-country: Belgium, Luxembourg, France	182	61(34-77)	0.121	Lung cancer	Cisplatin/ mitomycin/ Vindesine/ 5-FU	Fair
184	Morere JF	1994	France	109	57(40-75)	0.146	Lung cancer	FP	Fair
185	Villar-Grimalt A	1994	Multi-country: Spain, England, Italy	18	62(46-72)	0.5	Colorectal adenocarcinoma	5-FU/ Adriamycin	Fiar
186	Haarstad H	1994	Norway	30	56(17-71)	0.451	Renal cell carcinoma	5-FU/ IFN-α	Fair
187	Akhtar SS	1993	Jammu and Kashmir	100	49.96(30-70)	0.38	Mixed malignancies	FP or FM or 5-FU	Fair
188	Langer CJ	1993	USA	40	58(38-84)	0.45	Lung cancer	Epirubicin/ Cisplatin/ 5-FU+ RT	Fair
189	Depondt J	1993	France	150	55	0.1	Head and neck cancer	FP	Good
190	Carmo-Pereira J	1993	Portugal	36	54(32-68)	1	Breast carcinoma.	Mitoxantrone/ 5-FU/ LV/ Prednisone	Fair
191	Neri B	1993	Italy	35	58(38-72)	0.2	Gastric cancer	5-FU/ Epirubicin	Fair
192	Jassem J	1993	Multi-country: Hungary, Poland, Czech and Slovak Republic, The Netherland, Yugoslavia,	189	55(31-75)	0.132	Head and neck squamous-cell carcinoma	FP	Fair

			Croatia						
193	Keefe DL	1993	USA	910	47-68	NA	Mixed malignancies	5-FU based	Fair
194	Schober C	1993	Germany	390	27-75	0.75	Gastrointestinal cancer	LV5FU2	Fair
195	Levine MN	1993	Canada	288	≤60	1	Breast cancer	CEF	Fair
196	Kuzel TM	1993	USA	22	69(54-79)	1	Prostate cancer	5-FU	Fair
197	Citron ML	1992	USA	86	62(33-79)	0.24	Lung cancer	5-FU	Fair
198	de Forni M	1992	France	367	57(32-75)	0.373	Mixed malignancies	5-FU based	Fair
199	Zaniboni A	1991	Italy	30	NA	0.4	Gastric cancer	FAM	Fair
200	Gradishar W	1991	USA	244	35-83	NA	Mixed malignancies	5-FU based	Fair
201	Perit P	1991	Italy	31<br>29	≤75	1<br>1	Breast carcinoma	FEC<br>FNC	Good
202	Zamagni C	1991	Italy	30	56(36-71)	1	Breast cancer	FEC	Fair
203	Schlumberger M	1991	France	14	31-61	NA	Adrenal cortical carcinoma	ECF	Fair
204	Jeremic B	1990	Yugoslavia	65	63(40-72)	0.188	Mixed malignancies	FP	Fair
205	Samonigg H	1990	Austria	21	52.5(41-69)	1	Breast cancer	Doxorubicin/ 5-FU/ LV	Fair
206	Eskilsson J	1990	NA	58	61(39-76)	0.276	Mixed malignancies	FP	Fair
207	Havsteen H	1989	Denmark	11	56(46-64)	1	Breast cancer	CMF/ Doxorubicin	Fair
208	Rezkalla S	1989	Turi	25	60(41-79)	0.28	Head and neck squamous cell carcinomas	5-FU+FP	Fair
209	Eskilsson J	1988	Sweden	76	64(28-80)	0.118	Mixed malignancies	FP	Fair
210	Labianca R	1982	Milano	1083	<55:38.7%;>55:61%	NA	Mixed malignancies	5-FU or 5-FU based	Fair
211	Pottage A	1978	UK	140	NA	NA	Mixed malignancies	5-FU	Fair

Notes: a, Mixed malignancies: including two or more tumor types, such as breast cancer, colorectal cancer, gastric cancer, head and neck cancer, and so on; b, fluoropyrimidine: the article only stated that fluoropyrimidine agent was used, but did not specify which drug it was, or contained more than two types of fluoropyrimidines (e.g. 5-FU and capecitabine); c, data from both clinical trials (https://clinicaltrials.gov/) and published articles; NA: not available; RT: Radiotherapy; Cap: Capecitabine; Bev: Bevacizumab; Cet: Cetuximab; Pan: Panitumumab; Tra, Trastuzumab; Lap, Lapatinib.

Chemotherapy regimens as follows: CAF: Cyclophosphamide/ Adriamycin/ 5-FU; CapeOx: Oxaliplatin/ Cap; vinorelbine/ paclitaxel/5-FU; CapIRI: Cap/ Irinotecan; CCF: Doxorubicin/ Cisplatin/ 5-FU; CEF: 5-FU/ Epirubicin/ Cyclophosphamide; CMF: Cyclophosphamide/ Methotrexate/ 5-FU; CNF: Cyclophosphamide/ Mitoxantrone / 5-FU; ECX: Epirubicin/ cisplatin/ Cap; EDF: Epirubicin/ Docetaxel/ 5-FU; FAC: 5-FU/ Doxorubicin/ Cyclophosphamide; FAM: 5-FU/ Adriamycin/ Mitomycin-C; FE: 5-FU/ Doxorubicin/; FM: 5-FU/ Mitomycin-C; FMEP: 5-FU/ Epirubicin/ Mitomycin/ Cisplatin; FOLFOX: FN: 5-FU/ Vinorelbine; FOLFOXIRI: Irinotecan/ Oxaliplatin/ LV/ 5-FU; FOLFIRI: Irinotecan/ 5-FU/ LV; FP: 5-FU/ cisplatin; HDLV5-FU: High-dose/ LV; MFL: Mitoxantrone/ 5-FU/ LV; NEF: Vinorelbine/ Epirubicin/ 5-FU; Oxaliplatin/ LV(Leucovorin)/ 5-FU; SOX: Oxaliplatin/ S-1; SP: S-1/Cisplatin; TAF: Docetaxel/ Doxorubicin/ 5-FU; TPF: Docetaxel/ Cisplatin/ 5-FU; TX: Docetaxel/ Cap; XELOX: Oxaliplatin/ Cap; XELIRI: Irinotecan/ Cap; LV5FU2: LV/ 5-FU
