# Supplementary figures and images for: Redefining the Incidence and Profile of Fluoropyrimidine-Associated Cardiotoxicity in Cancer Patients: A Systematic Review and Meta-Analysis
Source: Pharmaceuticals (Basel). 2023 Mar 30;16(4):510. doi: 10.3390/ph16040510 (PMC10146083; doi:10.3390/ph16040510)

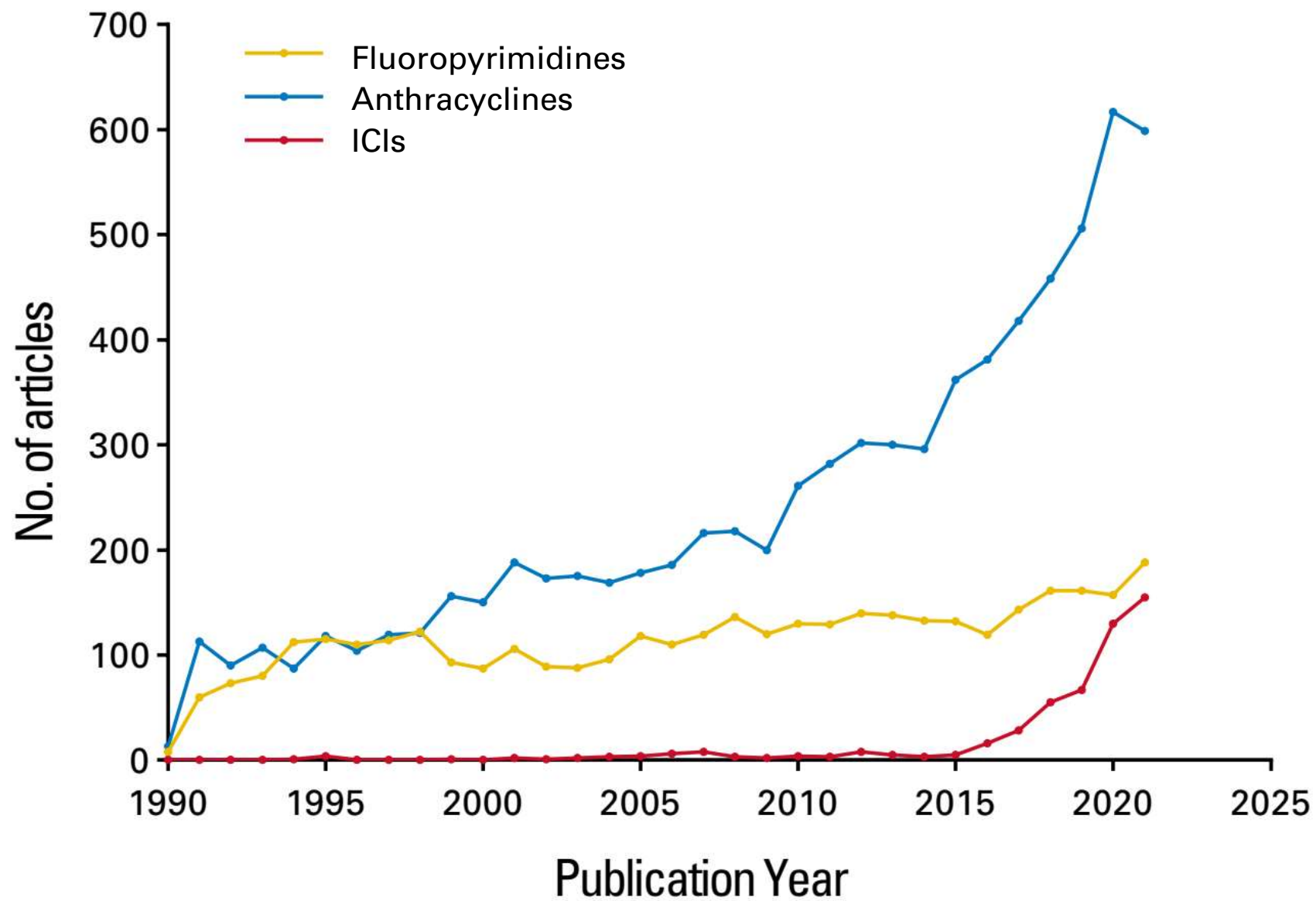

Supplement: Supplementary file 1 [file pharmaceuticals-16-00510-s001.zip › Figure S1 Number of articles published by year.pdf]

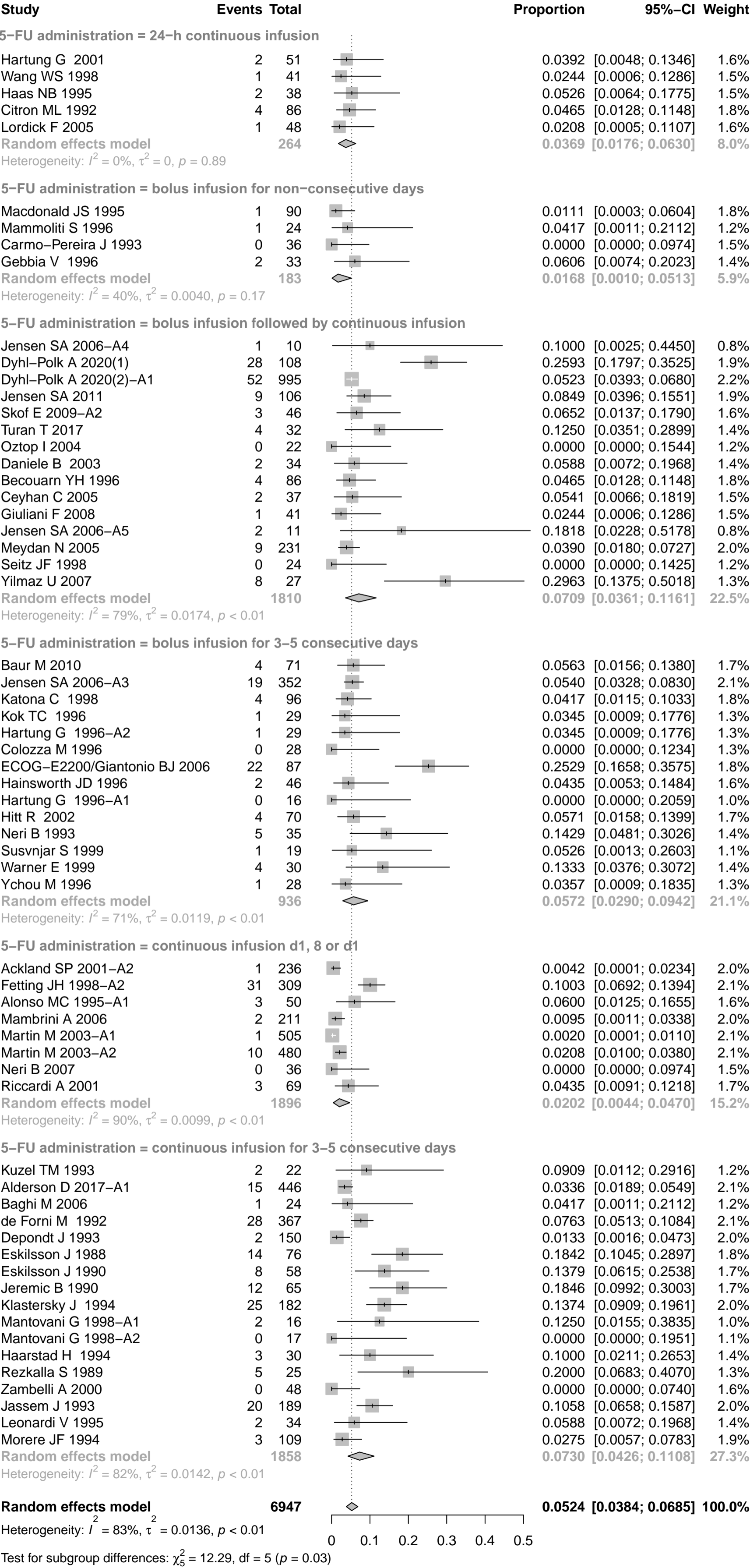

Supplement: Supplementary file 1 [file pharmaceuticals-16-00510-s001.zip › Figure S5 Forest plot for the subgroup analysis of 5-FU dosages.pdf]

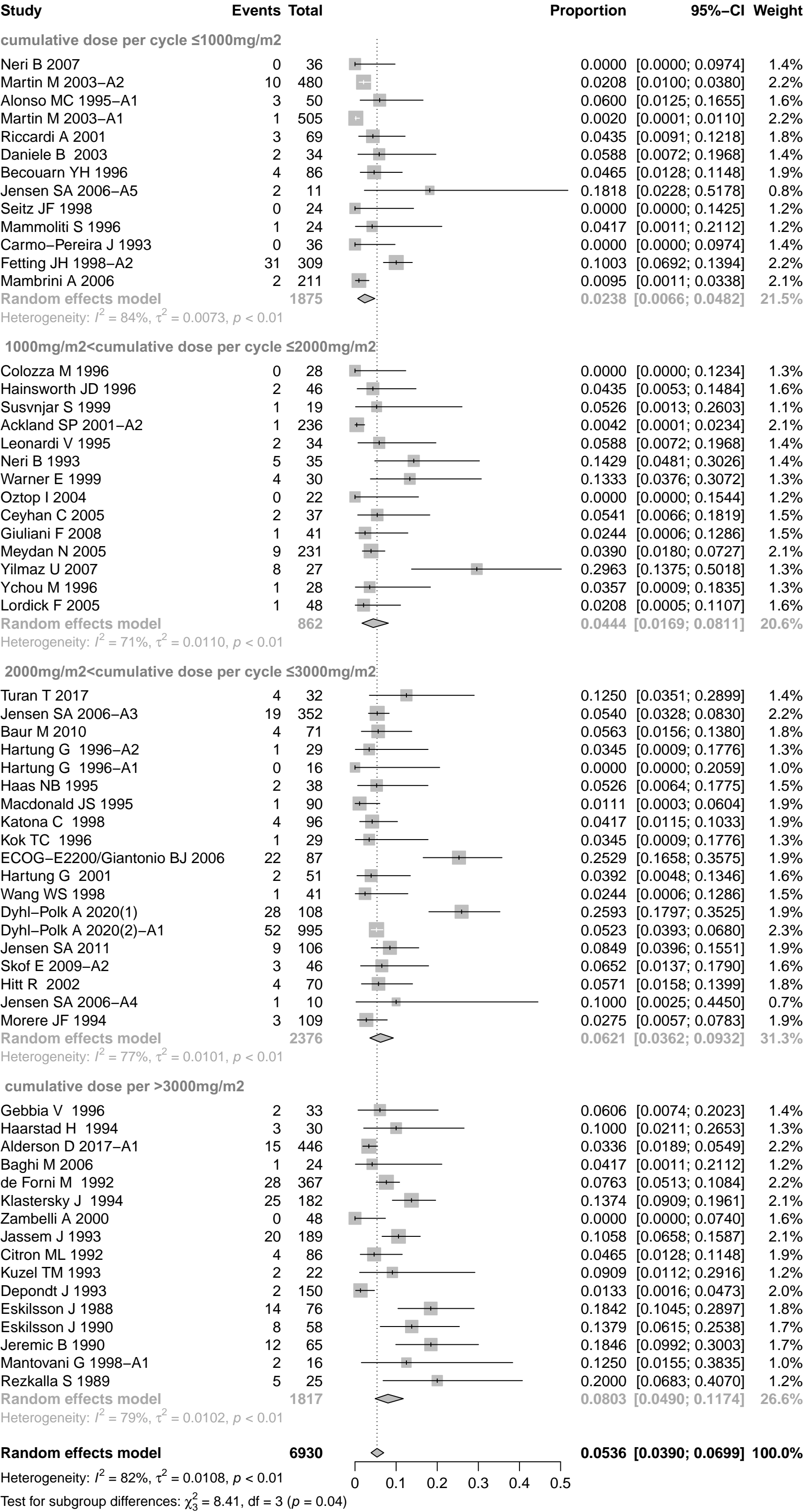

Supplement: Supplementary file 1 [file pharmaceuticals-16-00510-s001.zip › Figure S6 Subgroup analysis by 5-FU dose.pdf]

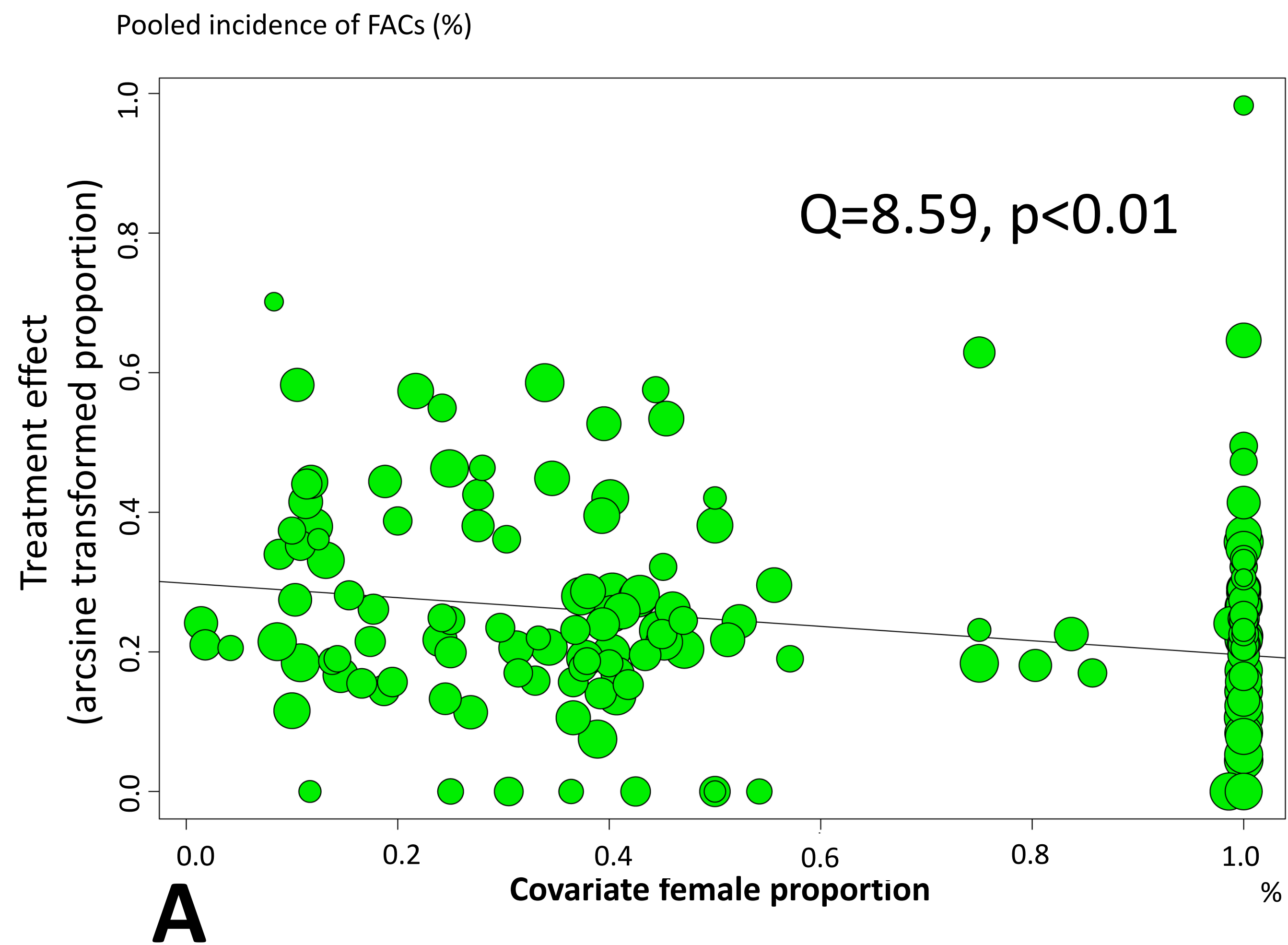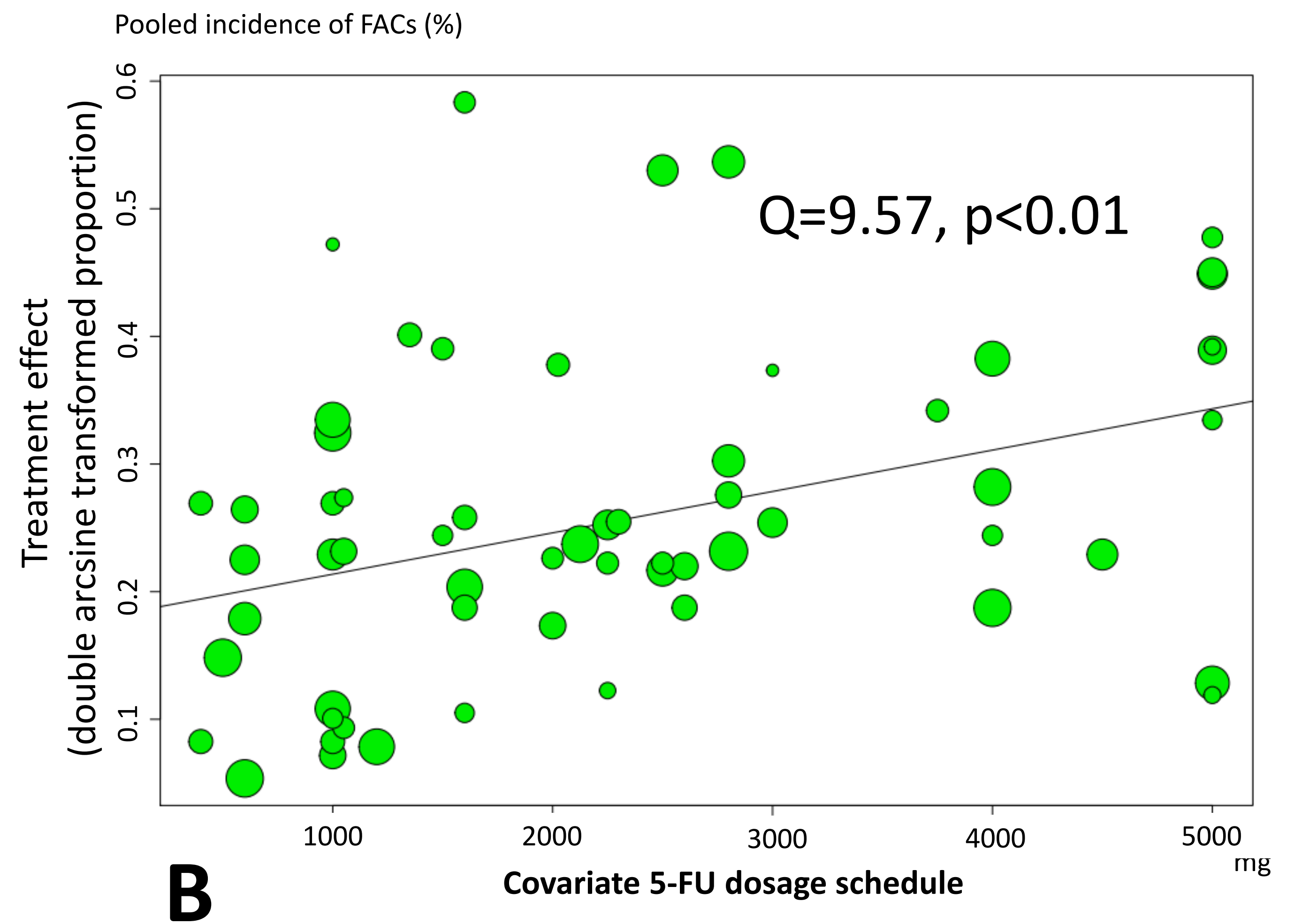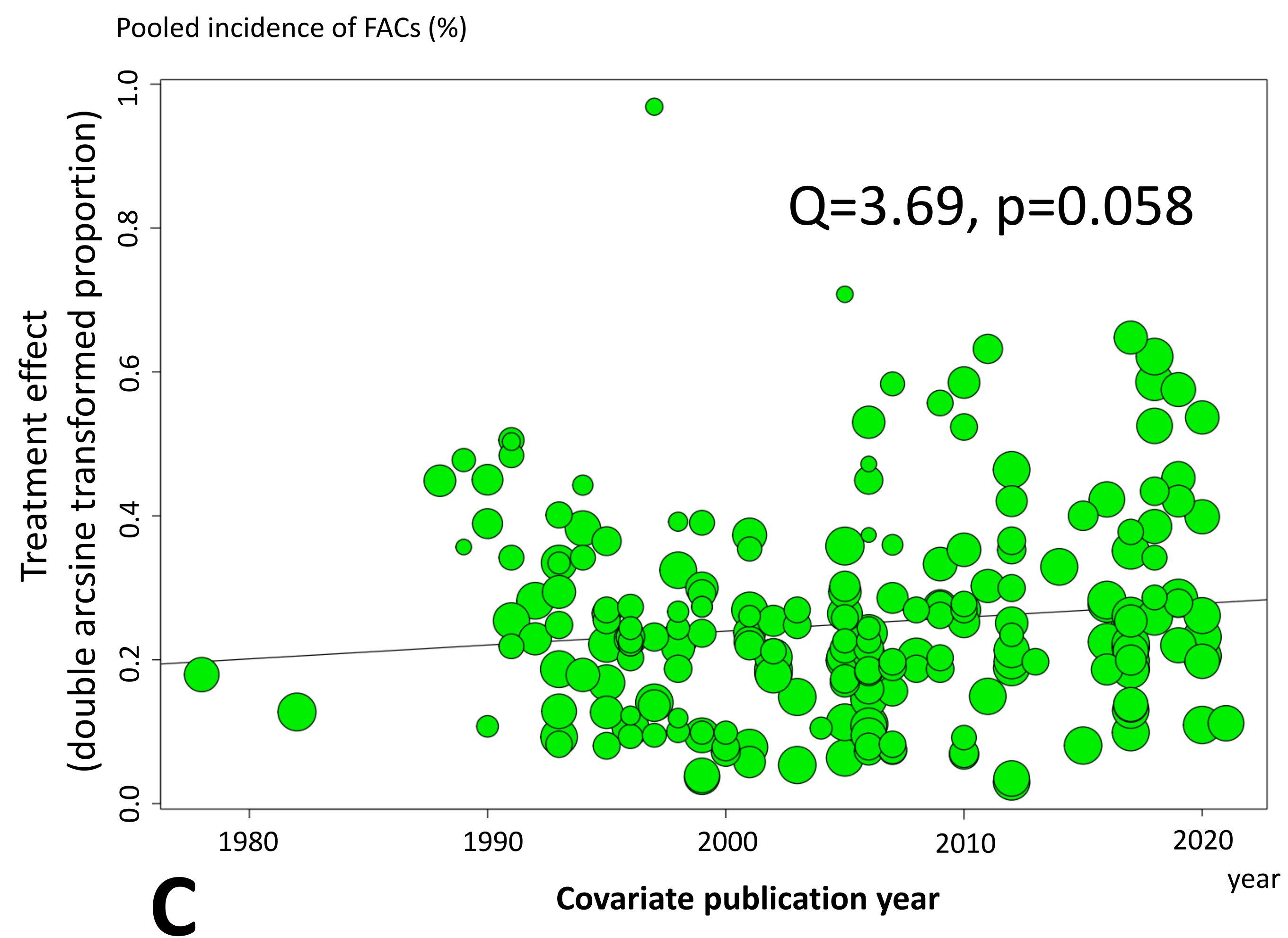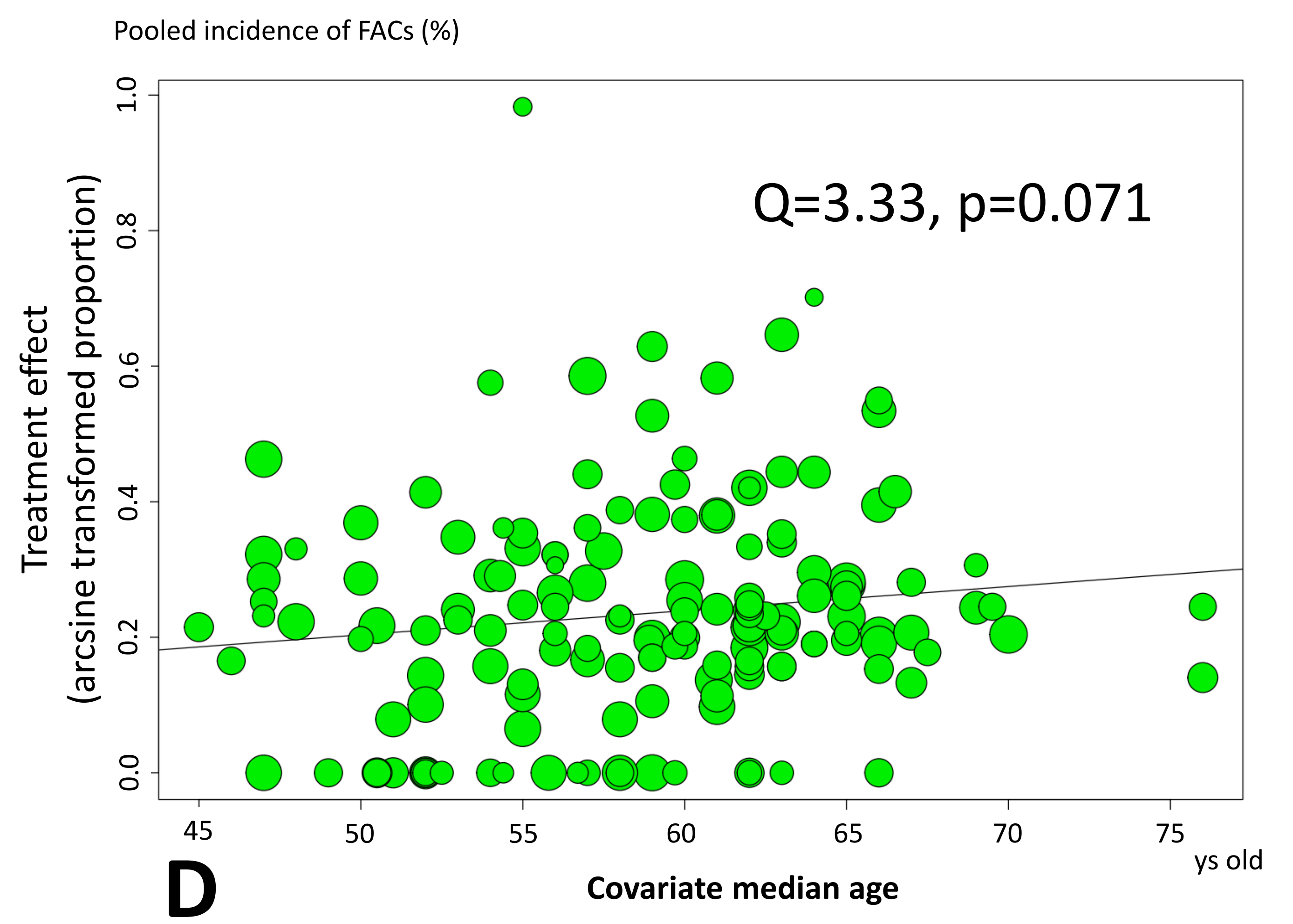

Supplement: Supplementary file 1 [file pharmaceuticals-16-00510-s001.zip › Figure S7 Bubble plot of the univariable meta-regression of continuous data .pdf]

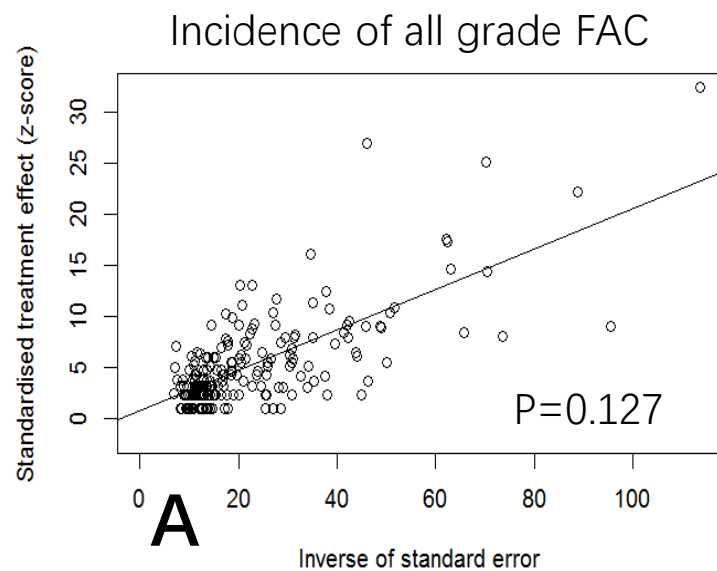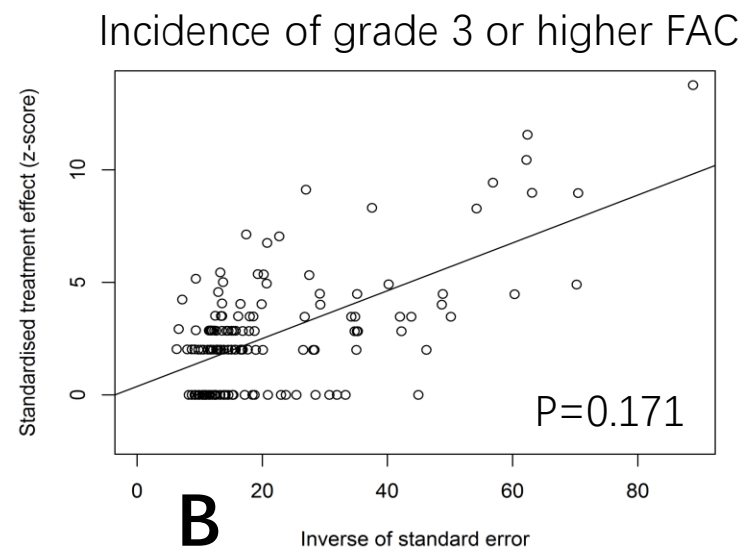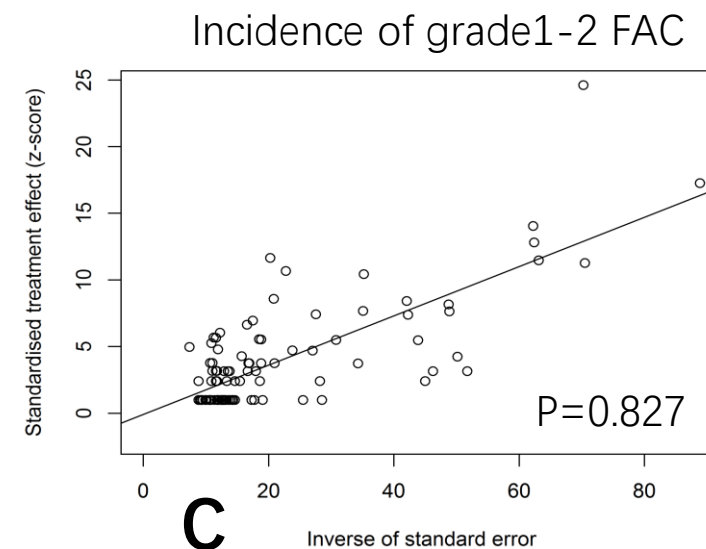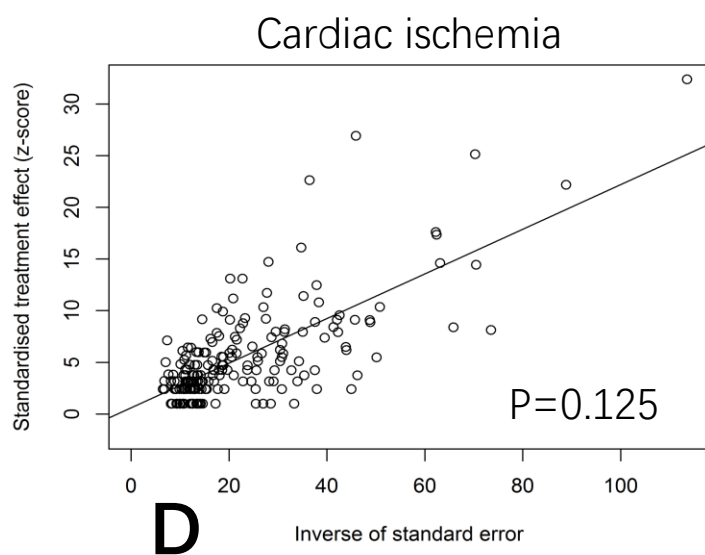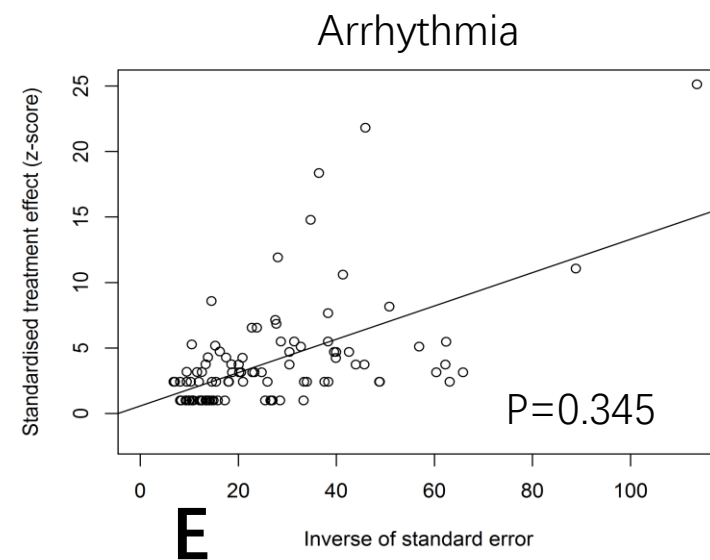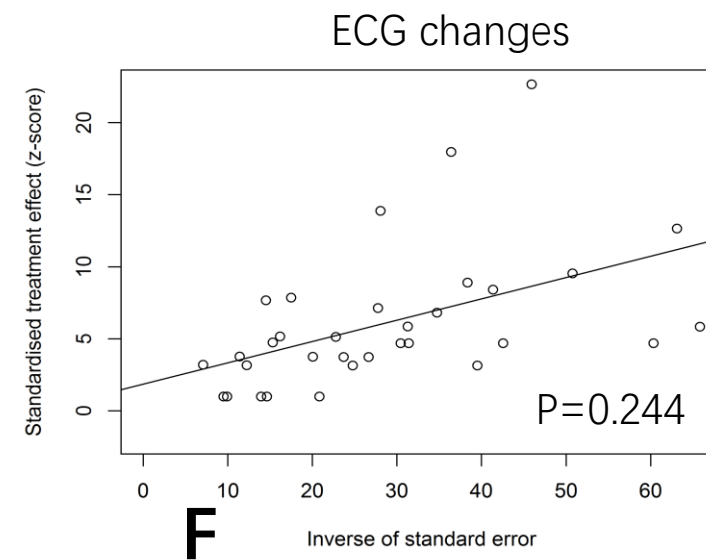

Supplement: Supplementary file 1 [file pharmaceuticals-16-00510-s001.zip › Figure S8A-F Eggers funnel plot.pdf]
